# Multi-omics profiling of cachexia-targeted tissues reveals a spatio-temporally coordinated response to cancer

**DOI:** 10.1038/s42255-025-01434-3

**Published:** 2026-01-15

**Authors:** Pauline Morigny, Michaela Vondrackova, Honglei Ji, Kristyna Brejchova, Monika Krakovkova, Konstantinos Makris, Radka Trubacova, Tuna F. Samanci, Doris Kaltenecker, Su-Ping Ng, Vignesh Karthikaisamy, Sophia E. Chrysostomou, Anna Bidovec, Mariana Ponce-de-Leon, Tanja Krauss, Claudine Seeliger, Olga Prokopchuk, Marc E. Martignoni, Melina Claussnitzer, Hans Hauner, Martina Schweiger, Laure B. Bindels, Mauricio Berriel Diaz, Stephan Herzig, Dominik Lutter, Ondrej Kuda, Maria Rohm

**Affiliations:** 1 Institute for Diabetes and Cancer, Helmholtz Center Munich, Neuherberg, Germany; 2 Joint Heidelberg-IDC Translational Diabetes Program, Inner Medicine 1, University Hospital, Heidelberg, Germany; 3 German Center for Diabetes Research, Munich, Germany; 4 Metabolism of Bioactive Lipids, Institute of Physiology of the Czech Academy of Sciences, Prague, Czech Republic; 5 Computational Discovery Research, Institute for Diabetes and Obesity, Helmholtz Center Munich, Neuherberg, Germany; 6 Institute of Molecular Biosciences, University of Graz, Graz, Austria; 7 Else Kröner Fresenius Center for Nutritional Medicine, School of Life Sciences, Technical University of Munich, Freising-Weihenstephan, Germany; 8 ZIEL Institute for Food and Health, Technical University of Munich, Freising-Weihenstephan, Germany; 9 Department of Surgery, Klinikum rechts der Isar, School of Medicine, Technical University of Munich, Munich, Germany; 10 The Novo Nordisk Foundation Center for Genomic Mechanisms of Disease, Broad Institute of MIT and Harvard, Cambridge, MA USA; 11 Diabetes Unit and Center for Genomic Medicine, Massachusetts General Hospital, Boston, MA USA; 12 Department of Medicine, Harvard Medical School, Boston, MA USA; 13 Institute of Nutritional Medicine, School of Medicine, Technical University of Munich, Munich, Germany; 14 BioTechMed-Graz, Graz, Austria; 15 Metabolism and Nutrition Research Group, Louvain Drug Research Institute, UCLouvain, Université catholique de Louvain, Brussels, Belgium; 16 Welbio Department, WEL Research Institute, Wavre, Belgium; 17 Chair Molecular Metabolic Control, Technical University Munich, Munich, Germany; 18 German Center for Cardiovascular Research, partner site Munich Heart Alliance, Munich, Germany

**Keywords:** Cancer metabolism, Metabolic disorders, Metabolism

## Abstract

Cachexia is a wasting disorder associated with high morbidity and mortality in patients with cancer. Tumour–host interaction and maladaptive metabolic reprogramming are substantial, yet poorly understood, contributors to cachexia. Here we present a comprehensive overview of the spatio-temporal metabolic reprogramming during cachexia, using integrated metabolomics, RNA sequencing and ^13^C-glucose tracing data from multiple tissues and tumours of C26 tumour-bearing male mice at different disease stages. We identified one-carbon metabolism as a tissue-overarching pathway characteristic for metabolic wasting in mice and patients and linked to inflammation, glucose hypermetabolism and atrophy in muscle. The same metabolic rewiring also occurred in five additional mouse models, namely Panc02, 8025, Apc^Min^, LLC and KPP, and a humanised cachexia mouse model. Together, our study provides a molecular framework for understanding metabolic reprogramming and the multi-tissue metabolite-coordinated response during cancer cachexia progression, with one-carbon metabolism as a tissue-overarching mechanism linked to wasting.

## Abstract (web summary)

Multi-omics profiling of diverse cancer cachexia models uncovers a multi-tissue metabolite-coordinated response associated with disease progression and links multi-tissue one-carbon metabolism to wasting.

## Main

Cachexia is a multifactorial wasting disorder affecting most patients with cancer and represents one of the strongest predictors of mortality in cancer. It leads to involuntary loss of body weight and muscle mass, as well as progressive functional impairment, ultimately causing poor quality of life, treatment compliance and survival in patients with cancer^1^. Reduced food intake (anorexia) is one component of cachexia. However, by definition, cancer cachexia is a metabolic syndrome that cannot be counteracted by conventional nutritional support^1^, indicating that maladaptive tumour–host metabolic reprogramming substantially contributes to the condition—a process that remains poorly understood.

Recent studies highlight the existence of a coordinated response of multiple tissues including tumour, liver, muscle or adipose tissue in cachexia, underpinning the multi-level nature of the syndrome^2–4^. It is assumed that tumour-secreted factors (for example, interleukin (IL)6) initiate a vicious cycle via the metabolic reprogramming of target tissues from the host, leading to body wasting. Subsequent alteration in circulating metabolites including amino acids^5^, amino acid derivatives^6^ or specific lipids^7,8^ can therefore distinguish between weight-stable and weight-losing cancer, highlighting the potential of metabolites as surrogate diagnostic and predictive biomarkers for defining disease pathology^9^. Nevertheless, few studies have incorporated metabolomics data from multiple tissues^10–17^, and most have focused on circulating and/or muscle metabolomes to define cachexia fingerprints. Integrating data from metabolomics and metabolite tracing across various tissues has the potential to elevate metabolite profiling beyond basic diagnostics towards actionable insights for preventative medicine^18,19^. This approach could enhance predictions on how metabolite profiles can be modulated to achieve desired metabolic outcomes.

**Extended Data Fig. 1:**
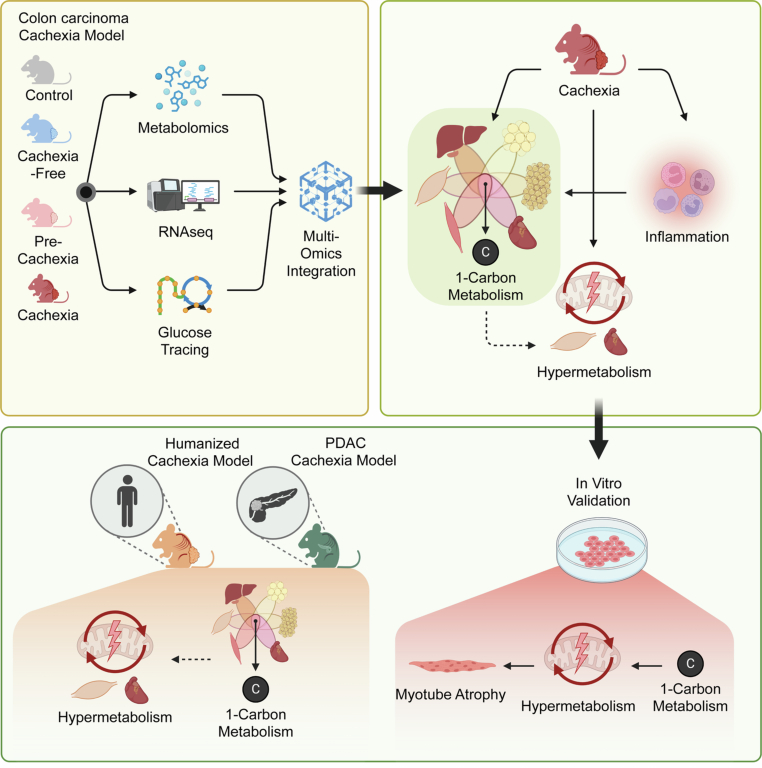
Schematic overview of the study. Figure created with BioRender.com.

Here, we present a metabolomics study of mice with cachexia-free cancer, pre-cachexia, and cachexia to track alterations in metabolic pathways across eight tissues during wasting progression. We identify one-carbon metabolism as a central dysregulated pathway in all cachexia target tissues and link it with hypermetabolism and atrophy of cachectic muscle and myotubes via ^13^C-glucose tracing. Multi-omics profiling further identifies key regulatory nodes linked to metabolic reprogramming, which are uniformly regulated across different types of cancer, and under the control of systemic inflammation. Our findings are substantiated by observations in patients with cancer with muscle wasting and a humanised cachexia mouse model. Overall, our study provides a molecular framework of tumour–host metabolic reprogramming in cancer cachexia and identifies a promising pathway altered systemically to pave the way for future research and interventional studies (Extended Data Fig. 1).

## Results

### Multi-tissue metabolomics in the C26 mouse model of cachexia reveals large cachexia-specific alterations in the metabolite profiles

**Fig. 1:**
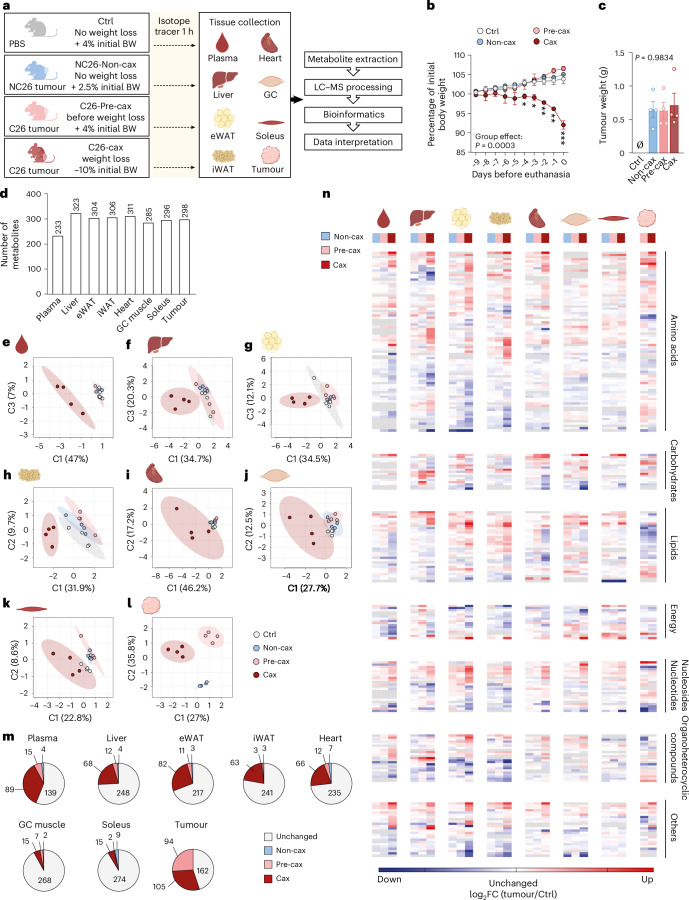
Multi-tissue metabolomics in the C26 mouse model of cachexia reveals large cachexia-specific alterations in the metabolite profiles. **a**, A schematic overview of the experimental workflow. Mice were divided into four groups: mice injected with PBS, healthy controls, no tumour (Ctrl, grey, no weight loss); mice injected with NC26 cancer cells, non-cachectic tumour controls (Non-cax, blue, no weight loss); mice injected with C26 cancer cells and killed before onset of weight loss, pre-cachectic tumour mice (Pre-cax, light red, no weight loss); mice injected with C26 cancer cells and killed once they developed cachexia, cachectic tumour mice (Cax, dark red, mean body weight (BW) loss of 10%). On the day of euthanasia, mice were fasted for 6 h and injected with an isotopic tracer ([^13^C_6_]-glucose). Tissues (plasma, liver, eWAT, iWAT, heart, GC muscles, soleus and tumour) were collected exactly 1 h later. Tissues were then processed for tracer metabolomics and results submitted to bioinformatics. *n* = 4 animals per group. See also Extended Data Figs. 2 and 3. **b**, Kinetics of body weight loss expressed as a percentage of initial body weight. **c**, Final tumour weight. Data are mean ± s.e.m. Statistical analysis: paired two-way ANOVA with Dunnett’s post-hoc tests versus Ctrl (**b**) and unpaired Kruskal–Wallis with Dunn’s post-hoc test (**c**). **d**, Total number of metabolites per tissue included in the analysis after filtering (Methods). See also Supplementary Table 1 (sum of all isotopologues; log-transformed imputed, scaled data) and our WebApp (https://m3cav.metabolomics.fgu.cas.cz/). **e**–**l**, PLSDA score plots of samples based on metabolites log-transformed imputed and scaled data for each organ, tumour and plasma; see icon legend in **a**. Ellipses represent 95% confidence intervals. **m**, Number of metabolites significantly altered in the time course of cachexia development. Grey: unchanged in Non-cax, Pre-cax and Cax versus Ctrl. Blue: significant in Non-cax versus Ctrl. Light red: significant in Pre-cax versus Ctrl. Dark red: significant in Cax versus Ctrl. List of significantly different metabolites per tissue can be found in Supplementary Table 1q–x. **n**, Heatmaps based on hierarchical clustering of all metabolites (Extended Data Fig. 3), which are significantly altered in at least one metabolic tissue of Cax mice, manually organised per metabolite class. Data are represented as log_2_ fold change (FC) (tumour group/controls). Tissues from left to right: plasma, liver, eWAT, iWAT, heart, GC muscle, soleus muscle, tumour. Groups from left to right: blue, Non-cax/Ctrl; light red, Pre-cax/Ctrl; dark red, Cax/Ctrl. Tumour: light red Pre-cax/Non-cax, dark red Cax/Non-cax. A list of metabolites and associated classes can be found in Supplementary Table 1y. **m**,**n**, Statistical analysis of filtered data: one-way ANOVA following post-hoc correction based on Tukey’s honestly significant difference procedure. Panel **a** and icons in **e**–**i**, **k** and **l** created with BioRender.com; icon in **j** reproduced from Servier Medical Art (https://smart.servier.com/) under a Creative Commons license CC BY 4.0. Source data

**Extended Data Fig. 2:**
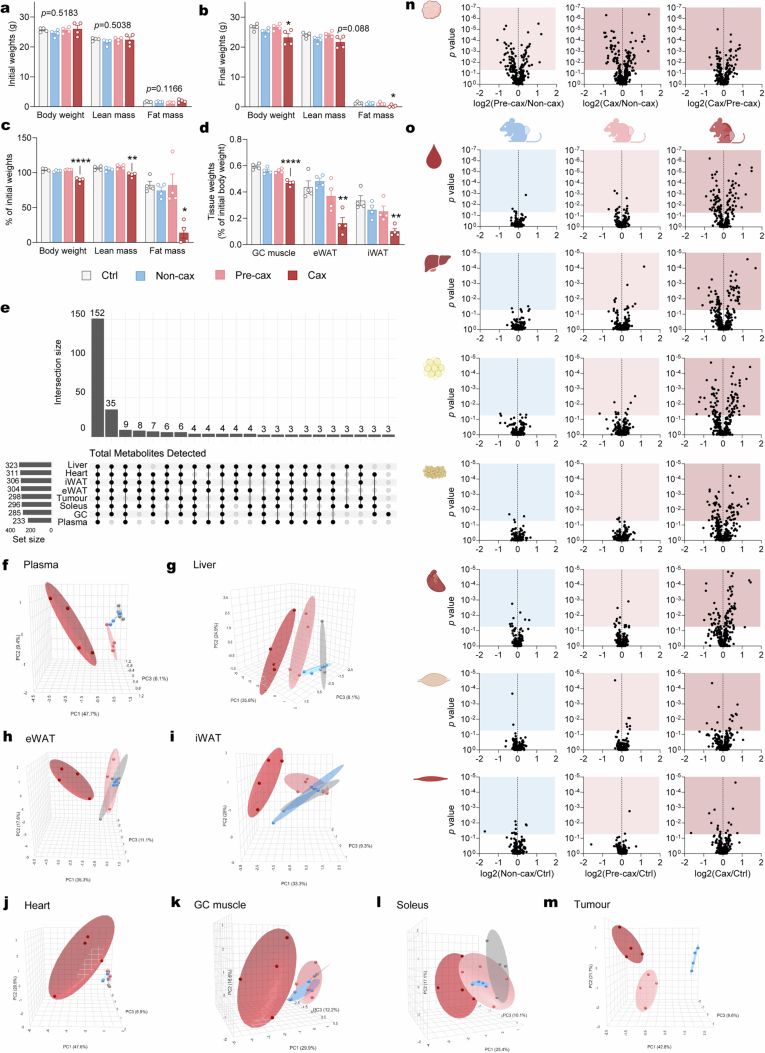
Multi-tissue metabolomics in the C26 mouse model. (**a-o**) See also Fig. 1a for experimental setup. Ctrl: healthy controls, no tumour; Non-cax: non-cachectic tumour mice; Pre-cax: pre-cachectic tumour mice; Cax: cachectic tumour mice. *n* = 4 animals per group. (**a-b**) Initial (**a**) and final (**b**) body, lean and fat mass. (**c**) Loss of body, lean and fat mass expressed as percentage of initial mass. (**d**) Final tissue weights (GC muscle, eWAT, iWAT). Data are mean ± s.e.m. Statistical analysis (a-d): unpaired one-way ANOVA with Dunnett’s post-hoc tests or unpaired Kruskal-Wallis with Dunn’s post-hoc tests *vs*. Ctrl. *p < 0.05, **p < 0.01, ****p < 0.0001 compared to Ctrl animals. (**e**) Upset plot showing all metabolites included in the analysis after filtering (see Methods). (**f-m**) 3D Principal Component Analysis (PCA) score plots of samples based on metabolite log-transformed, imputed and scaled data. Ellipses represent 95% confidence intervals. (**n-o**) Volcano plots showing the positive and negative alteration in the metabolome in the time course of cachexia development in tumour (**n**) and the different cachexia target tissues (**o**). From top to bottom: plasma, liver, eWAT, iWAT, heart, GC and soleus muscles. Data are presented as log2 fold change (Tumour groups/Ctrl). Statistical analysis of filtered data: one way ANOVA following post-hoc correction based on Tukey’s honesty significant difference procedure. Significant metabolites with a *p* value < 0.05 are highlighted in colored fields on each plot. See also Supplemental Data Table 1 for log-transformed imputed data, log2 fold change and *p* values for each tissue. Source data

We performed metabolomics from multiple tissues and the tumour in the well-established Colon 26 (C26) mouse model of cachexia (upon subcutaneous injection of C26 colon carcinoma cells)^20^. We assessed mice in either the pre-cachectic (before onset of weight loss, ‘Pre-cax’) or cachectic state (at ~10% average weight loss, ‘Cax’). As controls, we either injected mice with equal amounts of phosphate-buffered saline (PBS, ‘Ctrl’), or with non-cachexia-inducing NC26 cells (‘Non-cax’, mouse colon carcinoma)^8,21^ (Fig. 1a). Animals were fasted for 6 h to normalise glycaemia before performing metabolic assays and injected intraperitoneally with the stable isotopic uniformly labelled [^13^C_6_]-glucose tracer. Tumour and cachexia target tissues (plasma, liver, epididymal white adipose tissue (eWAT), inguinal white adipose tissue (iWAT), heart, gastrocnemius (GC) and soleus skeletal muscles) were collected 1 h later. Only C26 cachectic mice lost significant amounts of body weight, fat and lean mass, confirmed by tissue weights (Fig. 1b and Extended Data Fig. 2a–d). Tumour size was comparable between all groups (Fig. 1c).

Approximately 200–300 annotated polar metabolites per tissue were included in the analysis after filtering, covering a wide range of metabolite classes (Fig. 1d; for access to raw data, see Supplementary Table 1; for easy data visualization on our WebApp, see https://m3cav.metabolomics.fgu.cas.cz/). A subset of 152 common metabolites was detected in all tissues (Extended Data Fig. 2e). Partial least-squares discriminant analysis (PLSDA) and three-dimensional (3D) principal component analysis (PCA) showed that plasma, liver and adipose tissue metabolomes of C26 cachectic animals clearly clustered apart from all other groups (Fig. 1e–h and Extended Data Fig. 2f–i). Skeletal muscle and heart metabolite profiles showed a higher variability, especially in the cachectic group, and thereby no clear clustering was observed for these tissues (Fig. 1i–k and Extended Data Fig. 2j–l). NC26 and C26 tumours showed marked differences in their profiles despite equal size (Fig. 1l and Extended Data Fig. 2m,n). Non-cachexia-inducing NC26 tumours did not cause any major alterations in the metabolite profiles of host tissues compared with Ctrl (Fig. 1e–k and Extended Data Fig. 2f–l,o), highlighting that most metabolic alterations are associated with the presence of a cachexia-inducing tumour. The impact of cachexia varied across tissues with 5–38% of significantly altered metabolites (Fig. 1m and Extended Data Fig. 2o). The Pre-cax state was characterised by the alteration of a few metabolites, especially in plasma, liver, eWAT and heart, showing the progression towards a cachectic phenotype.

**Extended Data Fig. 3:**
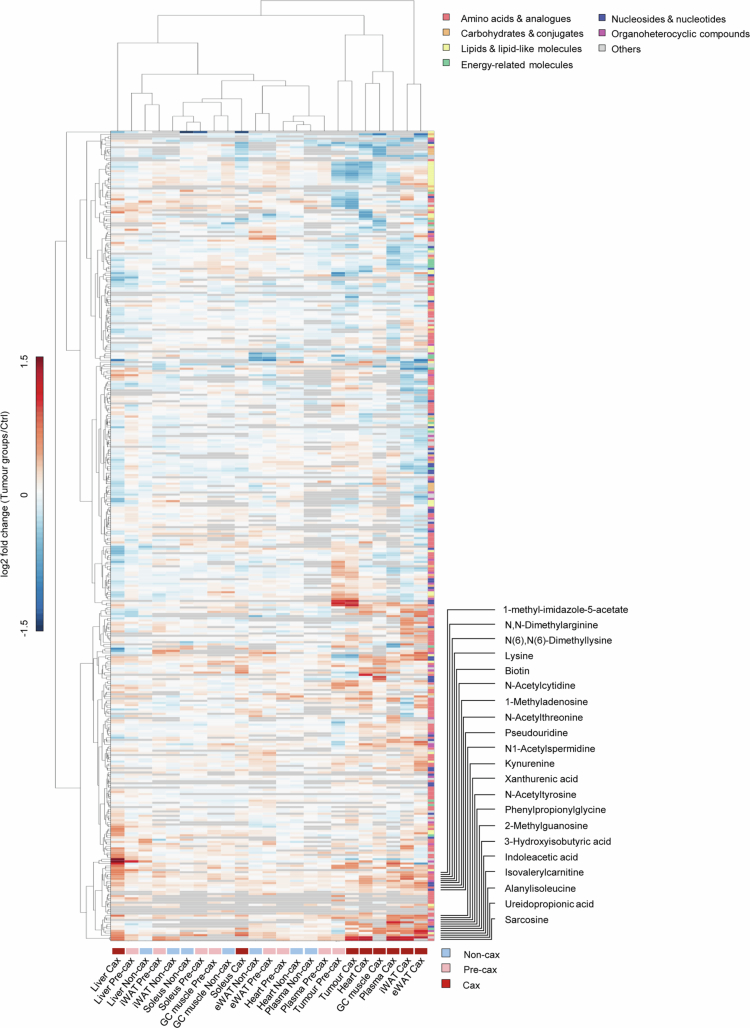
Hierarchical clustering. See also Fig. 1a for experimental setup. Ctrl: healthy controls, no tumour; Non-cax: non-cachectic tumour mice; Pre-cax: pre-cachectic tumour mice; Cax: cachectic tumour mice. *n* = 4 animals per group. Hierarchical clustering across all tissues and detected metabolites based on the log2 fold changes for each tissue sample of tumour mice compared to Ctrl. Key metabolites driving the clustering, and altered in cachexia-target tissues, are highlighted.

Hierarchical clustering across all tissues and detected metabolites revealed that most cachectic target tissues clustered apart from non-cachectic tissues, with the exception of the soleus muscle (Extended Data Fig. 3). The liver displayed a distinct metabolic profile of its own; however, the metabolites driving the clustering in other tissues were also enriched in cachectic liver. The cluster-defining metabolites were primarily associated with amino acid and nucleotide metabolism, including methylated metabolites, such as sarcosine, dimethyllysine, 1-methyladenosine and 2-methylguanosine (Extended Data Fig. 3). To highlight commonly changed metabolites within each class, we performed hierarchical clustering by metabolite class (amino acids, carbohydrates, energy, lipids, nucleosides, organoheterocyclic compounds and other) and tissue. This demonstrated that amino acids, nucleosides and organoheterocyclic compounds were jointly regulated between cachectic tissues (Fig. 1n and Supplementary Table 1q–y), suggesting a coordinated response during cachexia development.

### Multi-tissue metabolomics reveals coordinated increase in one-carbon metabolism in cachectic mice

**Extended Data Fig. 4:**
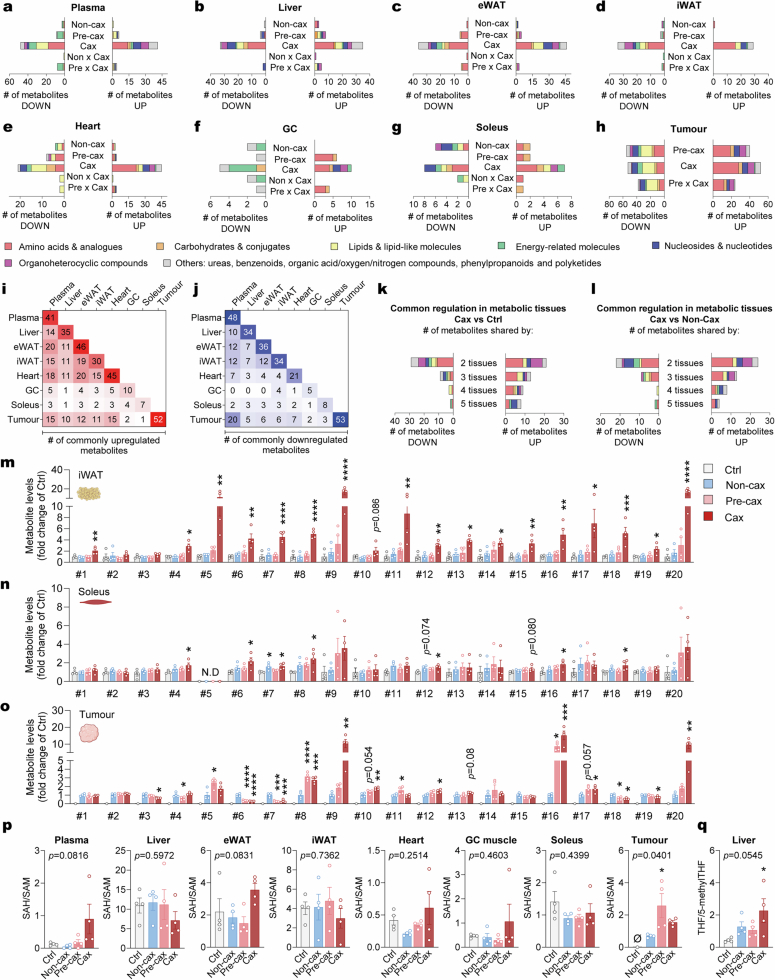
Multi-tissue metabolomics comparison. See also Fig. 1a for experimental setup. Ctrl: healthy controls, no tumour; Non-cax: non-cachectic tumour mice; Pre-cax: pre-cachectic tumour mice; Cax: cachectic tumour mice. *n* = 4 animals per group. (**a-h**) Number of metabolites significantly up- or down-regulated in the different tissues of Non-Cax, Pre-Cax and Cax mice compared to Ctrl, organised by class. Metabolites altered in a similar manner between different groups are depicted as follows: Non-Cax/Ctrl *vs*. Cax/Ctrl (Non x Cax), Pre-cax/Ctrl *vs*. Cax/Ctrl (Pre x Cax). (**i-j**) Tables showing the number of metabolites which are altered in a similar manner between the different tissues of Cax mice analysed: up-regulated metabolites (**i**), down-regulated metabolites (**j**). Comparison of Cax/Ctrl for cachexia-target tissues and Cax/Non-Cax for tumours. (**k-l**) Number of metabolites that are commonly up- or down-regulated in at least 2 cachexia target tissues (plasma, liver, adipose tissue, cardiac and skeletal muscles) of Cax mice compared to Ctrl (**k**) and Non-cax (**l**), excluding tumour tissues, and organised by class. Statistical analysis (a-l): one way ANOVA following post-hoc correction based on Tukey’s honesty significant difference procedure. See also Supplemental Data Table 1 for log-transformed imputed data, log2 fold change and *p* values for each tissue. (**m-o**) Bar graphs showing relative levels of substrates and products of one-carbon metabolism and related pathways in tumour groups *vs*. Ctrl. From top to bottom: iWAT (**m**), soleus (**n**) and tumour (**o**). Metabolites are similar to the list presented in Fig. 3b. (**p**) SAH/SAM ratio in cachexia-target tissues and tumour. (**q**) THF/5-methyl-THF ratio in liver. Data are mean ± s.e.m. Statistical analysis (m-o) based on raw data (MS signal intensities, arbitrary units A.U.). (m-q) One-way ANOVA with Dunnett’s post-hoc tests or Kruskal-Wallis with Dunn’s post-hoc tests *vs*. Ctrl (cachexia-target tissues) or Non-cax (tumours). *p < 0.05, **p < 0.01, ***p < 0.001, ****p < 0.0001 compared to Ctrl group. Source data

We next performed a more detailed analysis of the different classes of metabolites affected by cachexia. We mostly observed a decrease in energy-related metabolites (metabolites related to glycolysis, tricarboxylic acid (TCA) cycle and ketone bodies) in most of the metabolic tissues (Extended Data Fig. 4a–h), while the other classes did not show a unilateral change but rather a remodelling in their composition. Only a few metabolites were affected in the Non-cax and Pre-cax groups, and the Pre-cax group showed higher similarities to the Cax group (Pre × Cax comparison).

**Fig. 2:**
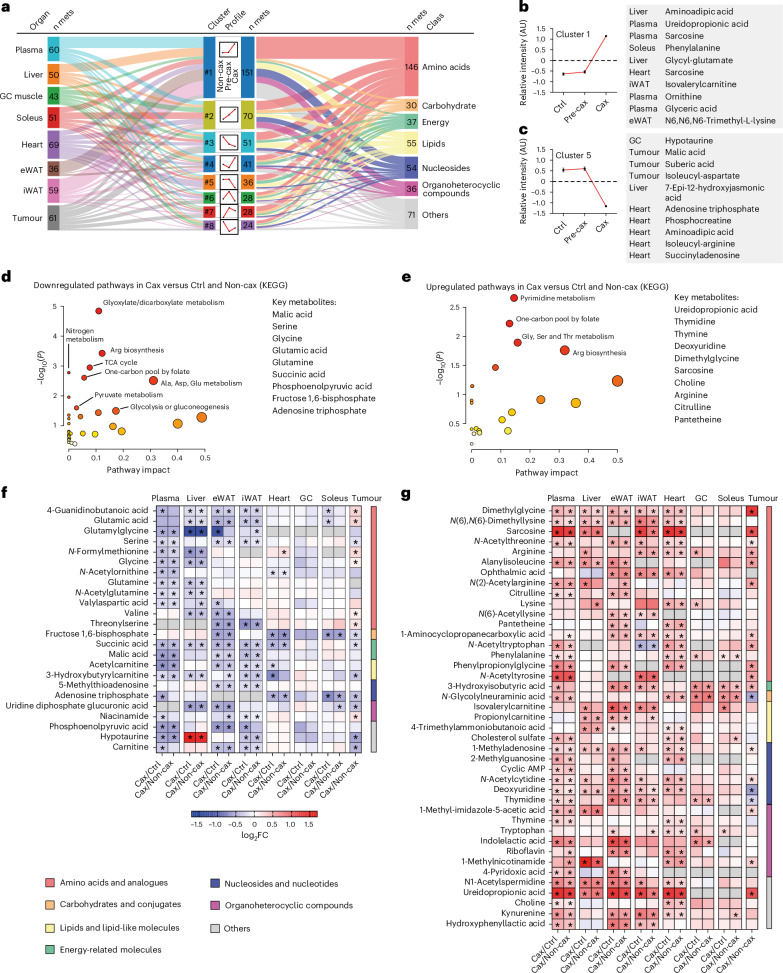
Multi-tissue metabolomics reveal coordinated increase in one-carbon metabolism in cachectic mice. See also Fig. 1a for the experimental set-up. Ctrl: healthy controls, no tumour; Non-cax: non-cachectic tumour mice; Pre-cax: pre-cachectic tumour mice; Cax: cachectic tumour mice. *n* = 4 animals per group. See also Extended Data Fig. 4 and Supplementary Fig. 1. **a**, Cluster analysis of metabolite trajectories in the time course of cachexia progression: Ctrl (cachexia target tissues) or Non-cax (tumours) → Pre-cax → Cax. Triple flow Sankey chart showing the most represented clusters from top to bottom, associated tissues (left) and metabolite classes (right). n mets indicates numbers of metabolites contributing to each cluster. **b**,**c**, Top ten metabolites of cluster 1 (upregulated in Cax) (**b**) and cluster 5 (downregulated in Cax) (**c**). **d**,**e**, Pathway analysis (Kyoto Encyclopedia of Genes and Genomes, KEGG) of metabolites that are commonly downregulated (**d**) or upregulated (**e**) in at least two cachexia target tissues of Cax versus Ctrl mice and Non-cax mice. **f**,**g**, Heatmaps showing metabolites commonly downregulated (**f**) or upregulated (**g**) between the different cachexia target tissues. Analysis based on log_2_ fold change (Cax/Ctrl and Cax/Non-cax) for cachexia target tissues and (Cax/Non-cax) for tumours. Statistical analysis: one-way ANOVA following post-hoc correction based on Tukey’s honestly significant difference procedure. See also Supplementary Table 1 for log-transformed imputed data, log_2_ fold change and *P* values for each tissue. Metabolites are organised by class. *P < 0.05.

To explore the dynamic changes in metabolites between organs, we defined pseudo-time profiles of metabolite levels as a sequence of control, pre-cachectic and cachectic states (Ctrl → Pre-cax → Cax, Non-cax instead of Ctrl for tumour), mimicking the temporal progression of cancer cachexia. We performed clustering of these profiles into eight scenarios: metabolites gradually increased or decreased, at an early or a late disease stage, and so on (Fig. 2a, middle). Each cluster consisted of 24–151 metabolites. The two largest clusters (#1 and #2) were predominantly composed of amino acids, nucleosides and organoheterocyclic compounds, showing an early (#2) or late (#1) increase towards the Cax state (Fig. 2a, right). Looking at the top ten metabolites defining each cluster (Fig. 2b,c and Supplementary Fig. 1), the most prominent cluster #1 (late increase in Cax) was defined by increased levels of several methylated amino acids (for example, sarcosine/methylglycine and trimethyllysine) and derivatives of amino acid metabolism (for example, aminoadipic acid, ureidopropionic acid, glycyl-glutamate and ornithine). Cluster #5 (late decrease in Cax) included metabolites associated with energy homeostasis (for example, malic acid, adenosine triphosphate and phosphocreatine), underlining the energy deficit in cachexia. Because the same metabolite could be present in different organs, the profiles were traced back to the original organ as a flowchart (Fig. 2a, left). Remarkably, all host tissues contributed to a similar degree to the different clusters, highlighting the coordinated tissue response to cachexia, whereas the tumour seemed to have a more distinct profile.

To identify metabolic signatures of cancer-induced metabolic dysfunction shared between host tissues, we next focused on metabolites that showed a significant up- or downregulation in at least two cachexia target tissues in Cax versus Ctrl and Non-cax animals (Fig. 2d–g and Extended Data Fig. 4i–l). Levels of these metabolites were altered in a cachexia-specific manner, as highlighted by the highly similar changes in Cax versus Ctrl and Cax versus Non-cax, respectively (Fig. 2f,g). ‘Amino acids and analogues’ was the most affected class. Next, by performing a pathway analysis of those metabolites (Fig. 2d,e; list in Fig. 2f,g), we confirmed that cachexia was associated with a low energetic status, as pathways related to glycolysis, glucose production or TCA cycle were significantly decreased (Fig. 2d,f). As previously reported^5^, amino acids in the circulation, including substrates of one-carbon metabolism (glycine and serine), were particularly depleted (Fig. 2d,f). Unexpectedly, we found that cachexia was associated with an increase in the products of one-carbon metabolism (for example, sarcosine and dimethylglycine) and related pathways, such as pyrimidine synthesis (products of the folate cycle: thymidine, thymine and ureidopropionic acid), and arginine biosynthesis/metabolism (Fig. 2e,g). Those results suggest a shift towards an increase in the products/substrates ratio of one-carbon metabolism-related metabolites in cachexia.

**Fig. 3:**
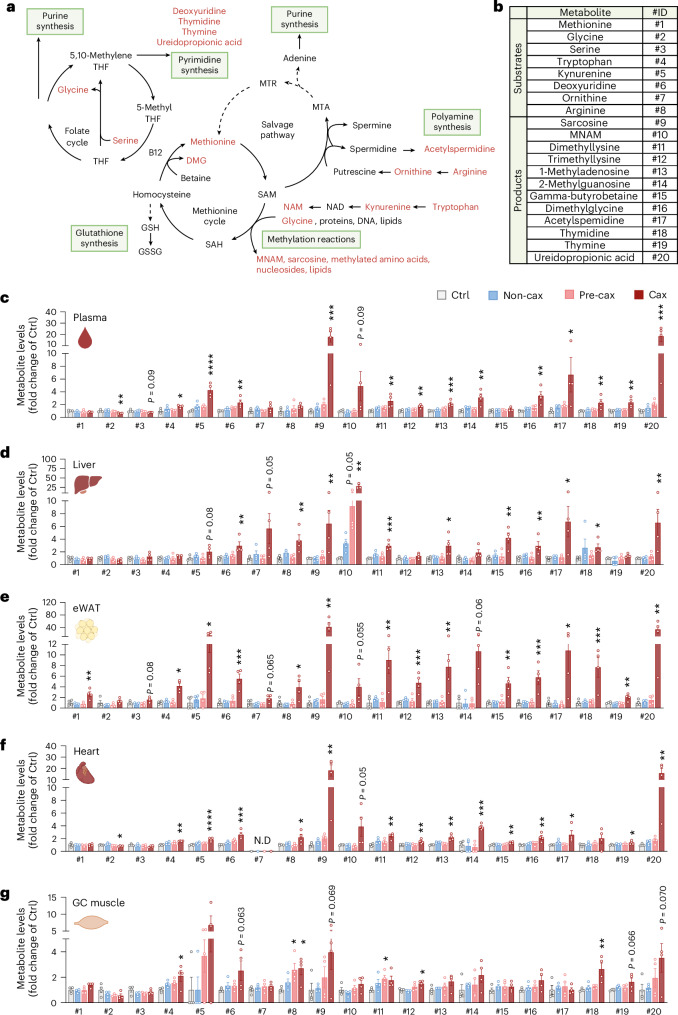
Significant increases in multiple substrates and products of the one-carbon metabolism in all host tissues indicate this pathway may be involved in cachexia aetiology. **a**, A graphical overview of one-carbon metabolism and related metabolic pathways. THF, tetrahydrofolate; DMG, dimethylglycine; GSH, reduced glutathione; GSSG, oxidised glutathione; SAH, *S*-adenosyl homocysteine; MTR, 5-methylthioribose; MTA, 5′-methylthioadenosine. **b**, A table showing substrates and products related to one-carbon metabolism, assigned to a specific identification number (ID). **c**–**g**, Bar graphs showing relative levels of substrates and products of one-carbon metabolism in Non-cax, Pre-cax and Cax tumour mice versus healthy controls (Ctrl), in plasma (**c**), liver (**d**), eWAT (**e**), heart (**f**) and GC muscle (**g**). *n* = 4 animals per group. See also Fig. 1a for the experimental set-up, and Extended Data Fig. 4. Data are the mean ± s.e.m. Statistical analysis based on raw data (MS signal intensities, arbitrary units (AU)). One-way ANOVA with Dunnett’s post-hoc tests versus Ctrl or Kruskal–Wallis with Dunn’s post-hoc tests versus Ctrl. *P < 0.05, **P < 0.01, ***P < 0.001, ****P < 0.0001 compared with the Ctrl group. N.D, not detected. Source data

One-carbon metabolism is central to multiple physiological processes including nucleotide and protein biosynthesis, redox defence and epigenetic regulation of gene expression^22^. It comprises the joint methionine and folate cycles, contributing to essential processes including methylation reactions (DNA, RNA, proteins and lipids), selenoamino acid production, purine, pyrimidine and glutathione synthesis (Fig. 3a). To run, the cycle requires several methyl acceptors (for example, niacinamide, also known as nicotinamide (NAM) derivative of tryptophan metabolism, glycine, lysine, nucleosides and lipids) leading to the production of methylated products (for example, 1-methylnicotinamide (MNAM), sarcosine, methyllysine, methyladenosine or guanosine). One-carbon metabolism is also tightly associated with polyamine metabolism for *S*-adenosylmethionine (SAM) recycling (salvage pathway)^23^. In our dataset, we checked the overall enrichment of a large subset of substrates and (in)direct products of one-carbon metabolism (Fig. 3a,b). We observed a clear increase in the levels of the vast majority of these metabolites in all tissues of Cax animals as well as in the tumour (Fig. 3c–g and Extended Data Fig. 4m–o), especially in products of this pathway (methylated products of the methionine cycle, such as sarcosine, MNAM, methyllysines, dimethylglycine; and products of the folate cycle, such as thymidine). This is also supported by trends towards elevated SAH/SAM ratios in Cax plasma, adipose tissue and tumour (Extended Data Fig. 4p) and THF/5-methylTHF in liver (the only tissue in which such metabolites were detectable; Extended Data Fig. 4q). The effect of cachexia on one-carbon metabolites was more pronounced in blood, liver and adipose tissues; nevertheless, we observed the same enrichment profile in cardiac and skeletal muscles (Fig. 3f,g and Extended Data Fig. 4n). Interestingly, we observed tissue specificity in terms of methylated products, with MNAM being the main methyl acceptor in liver, sarcosine and methyllysine in adipose tissue and muscles. Adjacent metabolic pathways, such as creatine synthesis, were also upregulated, as reflected by a significant enrichment of creatine levels in liver and adipose tissues (Supplementary Table 1). Overall, the enrichment in products of the one-carbon metabolism suggests an activation of this pathway.

The analysis of metabolites jointly altered in cachectic conditions across various tissues identified one-carbon metabolism as a tissue-overarching pathway important in cachexia and indicates that this pathway may be involved in the aetiology of the condition.

### Multi-omics integration identifies key nodes in cachexia-associated metabolic reprogramming under the control of IL6

**Extended Data Fig. 5:**
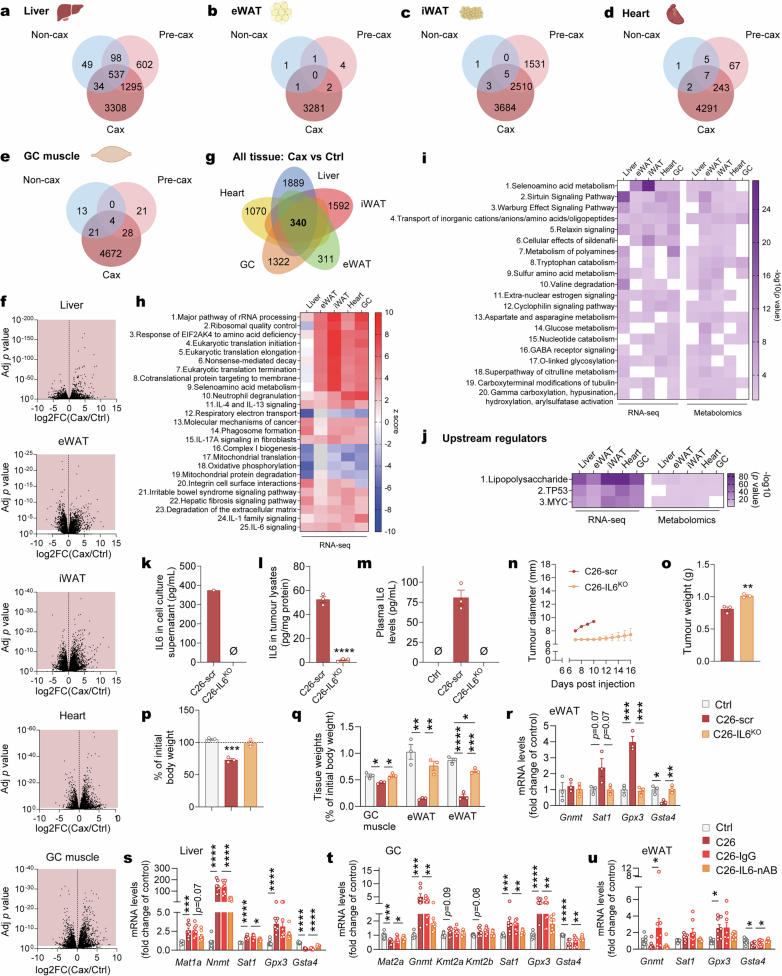
Multi-omics integration leading to interleukin-6. (**a-j**) Transcriptomic (RNA-seq) analysis of metabolic tissues (liver, eWAT, iWAT, heart, GC muscle) from healthy controls (PBS-injected, no tumour, Ctrl), non-cachectic (NC26 tumours, Non-cax), pre-cachectic (C26 tumours, Pre-cax) and cachectic (C26 tumours, Cax) mice (*n* = 4 animals per group, same animals as in Figs. 1–3). See also Fig. 4. Significant genes are defined by an adjusted *p* value < 0.05 and log2 fold change > 0 or < 0. (**a-e**) Venn diagrams showing the number of genes significantly altered in Non-cax, Pre-cax and Cax mice compared to Ctrl in liver (**a**), eWAT (**b**), iWAT (**c**), heart (**d**) and GC muscle (**e**). (**f**) Volcano plots showing the number of genes positively and negatively altered in Cax mice *vs*. Ctrl. Data presented as log2 fold change (Cax/Ctrl) and adjusted *p* values. Significant genes with an adjusted p value < 0.05 are highlighted in colored fields on each plot. (**g**) Venn diagram showing the number of genes significantly altered in Cax mice *vs*. Ctrl in the different metabolic tissues studied. (**h**) Top pathways altered in a similar manner in cachexia target tissues (liver, eWAT, iWAT, heart, GC muscle) in Cax *vs*. Non-cax mice. Data are represented as top z-scores: pathways predicted to be activated in red and inhibited in blue (IPA, Qiagen). (**i**) Top pathways commonly altered in both transcriptomics and metabolomics datasets based on *p* value (IPA, Qiagen) in Cax *vs*. Non-cax mice. (**j**) Top potential upstream regulators of observed changes in transcriptomics and metabolomics common to the different metabolic tissues of Cax mice *vs*. Non-cax mice (IPA, Qiagen). Data are represented as top significant pathways based on *p* value. (**k-r**) Mice were injected either with PBS (healthy controls, Ctrl, grey), control C26 cancer cells (C26-scramble, dark red), or C26 cancer cells with an IL6 knock-out (C26-IL6^KO^, orange). *n* = 3 animals per group. (**k**) Secretion of IL6 from control C26-scr and C26-IL6^KO^ tumour cells (*n* = 1 replicate), (**l**) IL6 concentrations in C26-scr and C26-IL6^KO^ tumour lysates (*n* = 3 animals per group), and (**m**) IL6 levels in plasma of C26-scr and C26-IL6^KO^ tumour bearing mice (*n* = 3 animals per group). (**n-o**) Kinetic of tumour growth (**n**) and final tumour weights (**o**). (**p**) Body weight loss, expressed as percentage of initial body weight. (**q**) Final tissue weights. (**r**) Relative mRNA expression level of key enzymes of one-carbon metabolism and related pathways in eWAT. Data expressed as fold change of Ctrl. (**s-u**) Mice were injected with PBS or C26 cancer cells and treated with a control IgG antibody or a neutralising IL6 antibody. From left to right: healthy controls (PBS-injected, Ctrl, grey), cachectic C26 tumour mice (C26, dark red), cachectic C26 tumour mice treated with control IgG (C26 + IgG, light red), C26 tumour mice treated with an IL6 neutralising antibody (C26 + IL6-nAB, orange). *n* = 8 animals per group. Relative mRNA expression of key enzymes of one-carbon metabolism and related pathways in liver (**s**), GC muscle (**t**) and eWAT (**u**). Data expressed as fold change of Ctrl. Statistical analysis on raw data (2^−ΔCt^ values) (**r**-**u**). Data are mean ± s.e.m. Statistical analysis: unpaired, non-adjusted, Student’s *t* test (**l**, **o**), unpaired one-way ANOVA with Tukey’s post-hoc tests (**p**-**r**), unpaired one-way ANOVA with Dunnett’s or Kruskal Wallis with Dunn’s post-hoc tests (s-t, *vs*. C26). *p < 0.05, **p < 0.01, ***p < 0.001, ****p < 0.0001. Source data

To identify targetable key nodes driving metabolic reprogramming in cachexia, we next performed RNA sequencing of cachexia target tissues of the same animals as used for metabolomics (Fig. 1a; raw data can be accessed under Gene Expression Omnibus (GEO) accession number GSE290937). The differences in the mRNA expression profile of Non-cax versus Ctrl mice were minor, indicating that a tumour per se is insufficient to induce major host tissue reprogramming (Extended Data Fig. 5a–e). By contrast, we observed a huge remodelling of tissue transcriptomes in Cax compared with Ctrl mice, with cachexia explaining 89% to near 100% of gene expression changes (depending on tissues) versus tumour explaining up to 11% changes (Extended Data Fig. 5a–f). Pre-cax mice showed an in-between profile between Non-cax and Cax mice, as expected. Overall, 340 genes were significantly altered in a similar manner between metabolic tissues of cachectic animals (Extended Data Fig. 5g).

**Fig. 4:**
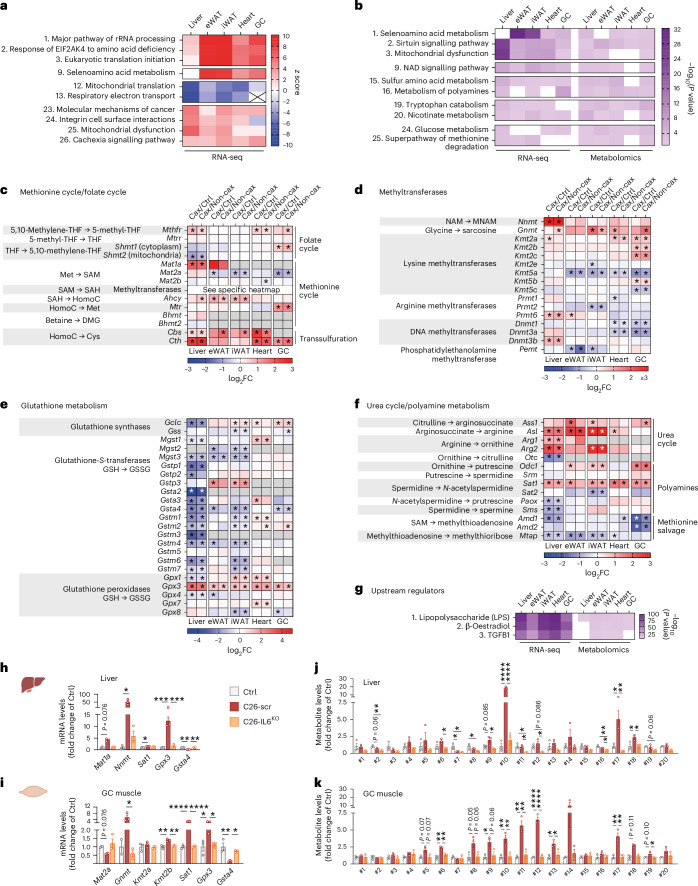
Multi-omics integration identifies key nodes in cachexia-associated metabolic reprogramming under the control of IL6. Transcriptomic analysis of cachexia target tissues from Ctrl, Non-cax and Cax tumour mice. See also Fig. 1a for the experimental set-up, and Extended Data Fig. 5. *n* = 4 animals per group. **a**, Top pathways altered in a similar manner in cachexia target tissues (liver, eWAT, iWAT, heart and GC muscle) from Cax versus Ctrl mice. Data are represented as top *z* scores: pathways predicted to be activated in red and inhibited in blue (IPA, Qiagen). **b**, Top pathways commonly altered in both transcriptomics and metabolomics datasets based on *P* value (IPA, Qiagen) in Cax versus Ctrl mice. Full pathway lists can be found in Supplementary Fig. 2a,b. See also Extended Data Fig. 5h–j for similar analyses in Cax versus Non-cax. **c**–**f**, Heatmaps showing the changes in mRNA expression of enzymes involved in one-carbon metabolism and related metabolic pathways (methionine cycle (**c**), methyltransferases (**d**), glutathione metabolism (**e**) and urea cycle (**f**)). Data from RNA sequencing analysis, presented as log_2_ fold change (Cax/Ctrl and Cax/Non-cax) and adjusted *P* values. *P < 0.05. *Ahcy*, adenosylhomocysteinase; *Amd*, *S*-adenosylmethionine decarboxylase; *Arg*, arginase; *Asl*, arginosuccinate lyase; *Ass*, arginosuccinate synthetase; *Bhmt*, betaine-homocysteine *S*-methyltransferase; *Cbs*, cystathionine beta-synthase; *Cth*, cystathionine gamma-lyase; *Dnmt*, DNA (cytosine-5)-methyltransferase; *Gclc*, glutamate-cysteine ligase catalytic subunit; *Gnmt*, glycine *N*-methyltransferase; *Gpx*, glutathione peroxidase; *Gss*, glutathione synthetase; *Gst*, glutathione *S*-transferase; *Kmt*, lysine (K)-specific methyltransferase; *Mat*, methionine adenosyltransferase; *Mgst*, microsomal glutathione *S*-transferase; *Mtap*, methylthioadenosine phosphorylase; *Mthfr*, methylenetetrahydrofolate reductase; *Mtr*, 5-methyltetrahydrofolate-homocysteine methyltransferase; *Mtrr*, 5-methyltetrahydrofolate-homocysteine methyltransferase reductase; *Nnmt*, NAM *N*-methyltransferase; *Odc*, ornithine decarboxylase; *Otc*, ornithine transcarbamylase; *Paox*, polyamine oxidase; *Pemt*, phosphatidylethanolamine *N*-methyltransferase; *Prmt*, protein arginine *N*-methyltransferase; *Sat*, spermidine/spermine N1-acetyltransferase; *Shmt*, serine hydroxymethyltransferase; *Sms*, spermine synthase; *Srm*, spermidine synthase. See also Supplementary Fig. 3 for visual integrations of transcriptomics and metabolomics data in Cax tissues. **g**, Top potential upstream regulators of observed changes in transcriptomics and metabolomics common to the different metabolic tissues of Cax versus Ctrl mice (IPA, Qiagen). Data are represented as top significant pathways based on *P* value. **h**–**k**, Relative mRNA expression levels of key enzymes (**h** and **i**) and metabolites (**j** and **k**) of one-carbon metabolism and related pathways in liver (**h** and **j**) and GC muscle (**i** and **k**) from healthy controls (PBS-injected, grey), C26-control tumour mice (C26-scramble (scr), dark red) and C26-IL6-knock out tumour mice (C26-IL6 KO, orange). Metabolite IDs as in the list presented in Fig. 3b. *n* = 3 animals per group. Data are the mean ± s.e.m. In **h**–**k**, statistical analysis on raw data (2^−ΔCt^ values and MS signal intensities, arbitrary units (AU)) was performed using one-way ANOVA with Tukey’s post-hoc tests or Kruskal–Wallis with Dunn’s post-hoc tests. *P < 0.05, **P < 0.01, ***P < 0.001, ****P < 0.0001. Source data

We performed a comparative pathway analysis of all genes significantly altered in the different target tissues of Cax versus Ctrl mice (Fig. 4a and Supplementary Fig. 2a) and Cax versus Non-cax mice (Extended Data Fig. 5h) and found that most pathways predicted to be activated in cachexia were related to amino acid metabolism, protein synthesis and post-translational modification (pathways #1–9), highlighting the general protein metabolism remodelling typical for cachexia^2^. These pathways (especially #9) also contain genes encoding for proteins related to one-carbon metabolism, such as the NAM *N*-methyltransferase (*Nnmt*, synthesis of MNAM), suggesting they are important targets for amino acid remodelling in cachexia. Other activated pathways were related to inflammatory processes (#10, #14, #18 and #21) and cancer/cachexia (#23 and #26). Meanwhile, all pathways predicted to be inhibited were related to energy production and mitochondrial function (#11, #12, #13, #15, #17 and #19). Next, seeking for potential mechanisms/pathways linking the aforementioned alterations in Cax transcriptome and metabolome, we performed an integrated pathway analysis of both datasets (Fig. 4b, Extended Data Fig. 5i and Supplementary Fig. 2b). Once again, the most significant pathways were related to amino acid metabolism (#1, #7, #12, #15, #19, #22 and #25), urea cycle and polyamine metabolism (#16 and #21), nucleotide metabolism (#9, #17 and #20), oxidative stress/detoxification signalling (#14 and #23) and energy metabolism (#2, #3, #13, #18, #24 and #26). In line with the enrichment of one-carbon metabolites, the combined analysis revealed selenoamino acid metabolism as a major hit, which contained many of the metabolites detailed in Fig. 3 (for example, MNAM and sarcosine) as well as the genes encoding for enzymes responsible for their production (for example, *Mat1a*, *Nnmt* and *Gnmt*). Overall, this analysis suggests a substantial, coordinated multi-level reprogramming during cachexia, which drives the deep change in tissue metabolism towards amino acid metabolism. Universal activation of one-carbon metabolism in all tissues may therefore support tissue reprogramming in cachexia through various downstream mechanisms involving protein translation and post-translational modifications.

We next performed a targeted analysis of the different enzymes related to one-carbon metabolism in tissues of Cax mice (Figs. 3a and 4c–f). Many of them were significantly modified across multiple tissues, suggesting the upstream reprogramming of amino acid metabolism in cachexia. We observed significantly altered gene expression of many enzymes linked to different arms of one-carbon metabolism, that is, methionine/folate cycles and methyltransferases, glutathione metabolism and polyamine metabolism. We observed several tissue-specific gene expression patterns, in line with metabolite levels (Fig. 3). While *Mat1a* (the gene product responsible for the conversion of methionine to SAM) and *Nnmt* (the gene product responsible for the conversion of NAM into MNAM) were strongly induced in livers of cachectic animals, in line with a previous report^24^, regulation of *Mat2a*, *Kmt2a* and *Kmt2b* (encoding enzymes producing methyllysines) was more specific to cachectic muscle. In addition to tissue-specific gene expression changes, several changes were common across tissues, such as spermidine/spermine N1-acetyltransferase (*Sat1*) (suggesting an interconnection between methionine cycle and polyamine metabolism), glutathione peroxidase (*Gpx3*) and glutathione *S*-transferase (*Gsta4*) (suggesting alterations in detoxification processes). Pathway maps highlighting significantly altered metabolites and genes in the different tissues of Cax mice can be found in Supplementary Fig. 3.

An upstream regulator analysis of the combined transcriptome and metabolome datasets identified lipopolysaccharide and, by extension, inflammation as the first determinant to drive the substantial metabolic reprogramming occurring in cachexia (Fig. 4g and Extended Data Fig. 5j). IL6 has previously been described as a tumour-secreted and key inflammatory factor driving cachexia in the C26 model^25–27^. Thus, we assessed whether IL6 controls expression of some signature genes (*Mat1a*, *Mat2a*, *Nnmt*, *Gnmt*, *Kmt2a*, *Kmt2b*, *Sat1*, *Gpx3* and *Gsta4*) in C26 tumour-bearing mice in which the *Il6* gene was deleted from tumour cells (Extended Data Fig. 5k–m) or animals treated with an IL6-neutralising antibody. In both experiments, IL6 inhibition did not reduce tumour size (Extended Data Fig. 5n,o) but improved cachexia-associated weight loss (Extended Data Fig. 5p,q)^25,27^. Note that C26-IL6-knockout (KO) tumours grew slower than C26-scr tumours but eventually reached a comparable size at endpoint without inducing cachexia (Extended Data Fig. 5n–q). We confirmed the substantial regulation of genes related to one-carbon metabolism by IL6 in C26 cachexia in liver, GC muscle and adipose tissue in these two independent cohorts (Fig. 4h,i and Extended Data Fig. 5r–u). For instance, *Nnmt* induction in liver by C26 tumours was repressed by 77% with IL6-neutralising antibody, and by 89% upon IL6 KO in C26 cells. Metabolomics confirmed the significant increase of multiple products of the one-carbon metabolism in C26 cachectic liver (for example, #10 MNAM and #18 thymidine) and GC muscle (for example, #11 di- and #12 tri-methyllysine) and the near-complete absence of their enrichment upon IL6 KO (Fig. 4j,k).

Overall, our analysis suggests a coordinated remodelling of the tissue transcriptomes and metabolomes in cachexia, in part driven by the pro-inflammatory status of cachectic animals, that activates one-carbon metabolism in a universal manner to sustain the remodelling of protein metabolism inherent to cachexia.

### Overactivation of the methionine cycle drives atrophy and metabolic dysfunction in myotubes

**Fig. 5:**
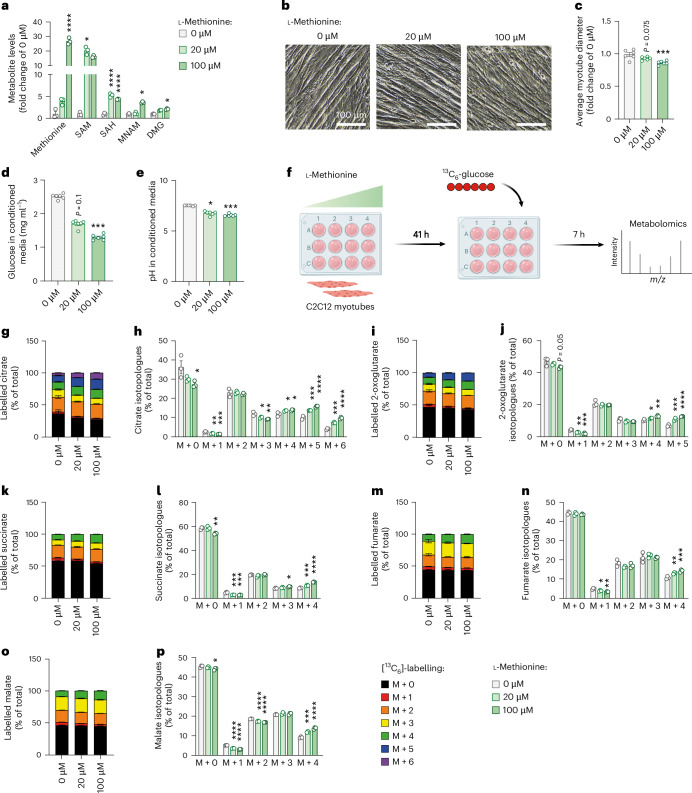
Overactivation of the methionine cycle drives atrophy and metabolic dysfunction in myotubes. C2C12 myotubes were treated with different doses of L-methionine (0 µM, 20 µM and 100 µM) for 48 h. **a**, Relative levels of substrates and products of one-carbon metabolism, presented as fold change of 0 µM condition (*n* = 3 replicates per group). Statistical analysis was performed on MS signal intensities. **b**,**c**, Representative images (**b**) and quantification of myotube diameters (**c**) (*n* = 6 replicates per group). **d**,**e**, Glucose levels (**d**) and pH of culture media (**e**) (*n* = 6 replicates per group). **f**–**p**, Metabolic tracing experiment in C2C12 cells: after 41 h of treatment with L-methionine, glucose from media was replaced by [^13^C_6_]-glucose supplemented with different doses of L-methionine, and cells were treated for another 7 h, and then samples were submitted to tracer metabolomics (**f**); incorporation of labelled carbons from [^13^C_6_]-glucose into metabolites of the TCA cycle (*n* = 3 replicates per group) (**g**–**p**). Unlabelled metabolites are referred as M + 0 and isotopically labelled metabolites as M + *X*, where *X* represents the number of labelled carbon atoms. Data are presented as the percentage of total metabolite levels. Stacked bar graphs (**g**, **i**, **k**, **m** and **o**) show the overall isotopologue levels; bar graphs (**h**, **j**, **l**, **n** and **p**) show the levels of each individual isotopologue (citrate (**g** and **h**), 2-oxoglutarate (**i** and **j**), succinate (**k** and **l**), fumarate (**m** and **n**) and malate (**o** and **p**)). MS signal intensity (arbitrary units, AU) can be found in Extended Data Fig. 6. Data are the mean ± s.e.m. Statistical analysis: one-way ANOVA with Dunnett’s post-hoc tests or Kruskal–Wallis with Dunn’s post-hoc tests versus the 0 µM condition. *P < 0.05, **P < 0.01, ***P < 0.001, ****P < 0.0001. Panel **f** created with BioRender.com. Source data

**Extended Data Fig. 6:**
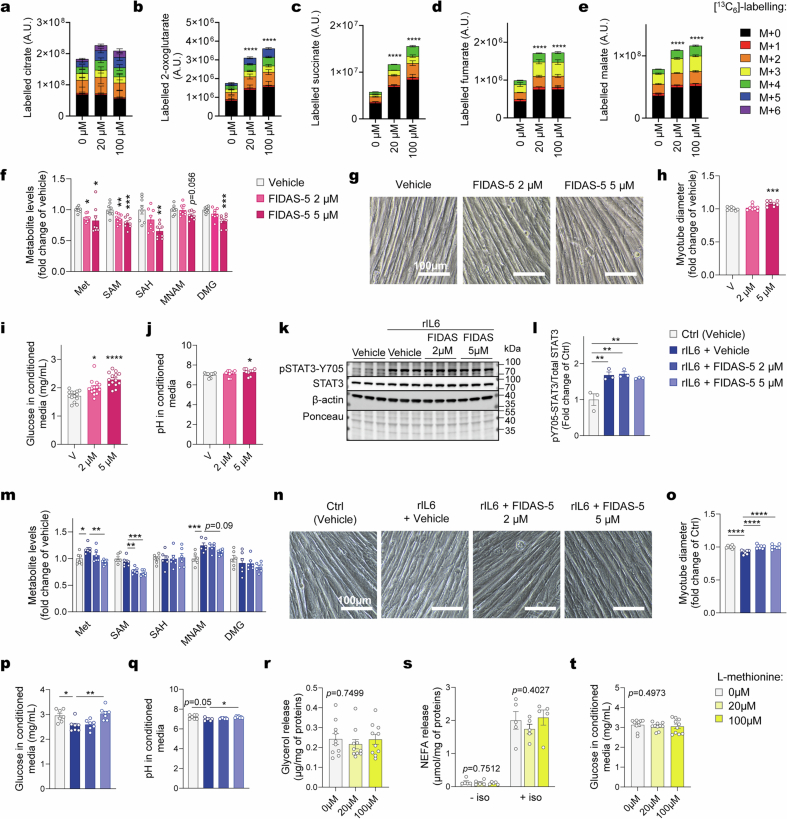
Atrophy and metabolic dysfunction in myotubes. (**a-e**) C2C12 myotubes were treated with different doses of L-methionine (0 µM, 20 µM, 100 µM) for 48 h. See also Fig. 5. (**a-e**) Incorporation of labelled carbons from [^13^C_6_]-glucose into metabolites of the TCA cycle (*n* = 3 replicates per group). Unlabelled metabolites are referred as M + 0, isotopically-labelled metabolites as M + X. Data are presented as MS signal intensities (arbitrary units A.U.). (**f-j**) C2C12 myotubes were treated with different doses of FIDAS-5 (methionine adenosyltransferase inhibitor) for 48 h. (**f**) Relative levels of substrates and products of one-carbon metabolism, presented as fold change of vehicle condition (*n* = 8 replicates per group). (**g-h**) Representative images (**g**) and quantification of myotube diameters (**h**) (*n* = 8 replicates per group). (**i-j**) Glucose levels (*n* = 14 replicates per group) (**i**) and pH of culture media (**j**) (*n* = 9 replicates per group). (**k-q**) C2C12 myotubes were treated with 100 ng/mL of recombinant IL6 and different doses of FIDAS-5 for 48 h. (**k-l**) Validation of the activation of rIL6 signalling cascade *via* pY705-STAT3 protein levels (*n* = 3 replicates per group, representative experiment out of 3 independent experiments). (**m**) Relative levels of substrates and products of one-carbon metabolism, presented as fold change of vehicle condition (*n* = 6 replicates per group). (**n-o**) Representative images (**n**) and quantification of myotube diameters (**o**) (*n* = 7 replicates per group). (**p-q**) Glucose levels (**i**) and pH of culture media (**j**) (*n* = 7 replicates per group). (**r-t**) 3T3-L1 adipocytes were treated with different doses of L-methionine (0 µM, 20 µM, 100 µM) for 24-48 h. (**r-s**) Glycerol (*n* = 10 replicates per group) (**s**) and NEFA (**t**) release after 24 h incubation with L-methionine (readouts for lipolytic activity). NEFA release was measured in the presence or absence of isoproterenol to assess stimulated and basal lipolysis, respectively (*n* = 5 replicates per group). (**t**) Glucose levels of culture media after 48 h of incubation (n = 10 replicates per group). Data are mean ± s.e.m. Statistical analysis: unpaired two-way ANOVA with Dunnett’s post-hoc tests (**a**-**e**, *vs*. 0 µM), unpaired one-way ANOVA with Dunnett’s post-hoc tests or Kruskal-Wallis with Dunn’s post-hoc tests (**f**-**j**, *vs*. 0 µM; l, *vs*. Ctrl vehicle; **m**-**q**, *vs*. rIL6 vehicle). *p < 0.05, **p < 0.01, ***p < 0.001, ****p < 0.0001 *vs*. 0 µM or vehicle (**a**-**j**, **r**-**t**), Ctrl + vehicle (**l**) or *vs*. rIL6 + vehicle (**m**-**q**). Source data

To functionally connect the activation of one-carbon metabolism with cachexia phenotypes, we treated C2C12 myotubes with different doses of L-methionine to experimentally induce one-carbon metabolism, confirmed by increased levels of associated metabolites (Fig. 5a). Interestingly, L-methionine induced myotube atrophy, a classical cachexia feature in this cell culture setting^28^, in a dose-dependent manner (Fig. 5b,c). Furthermore, L-methionine altered myotube metabolism towards higher glucose consumption, as indicated by lower glucose and lower pH in the cell medium (Fig. 5d,e). We thus applied the non-radioactive [^13^C_6_]-glucose tracer to L-methionine-treated C2C12 myotubes (Fig. 5f). Activation of one-carbon metabolism in myotubes induced a substantial glucose hypermetabolism (that is, condition defined by an accelerated metabolism), as indicated by the dose-dependent increase in total and labelled metabolites of the TCA cycle (Extended Data Fig. 6a–e) and the particularly higher proportion of labelled isotopologues (M + 4 and higher where applicable) (Fig. 5g–p).

To further support the link between one-carbon metabolism, myotube diameter and glucose consumption, we next applied a specific inhibitor of the methionine cycle (FIDAS-5, inhibitor of methionine adenosyltransferase) to C2C12 cells. As expected, FIDAS-5 significantly reduced one-carbon metabolite levels (Extended Data Fig. 6f) and led to a phenotype opposite to L-methionine-treated cells in a dose-dependent manner, characterised by myotube hypertrophy associated with reduced glucose consumption, suggesting hypometabolism (Extended Data Fig. 6g–j).

As we have shown in vivo the importance of IL6 in inducing one-carbon metabolism in cachexia, we next assessed the capacity of FIDAS-5 to counteract cachexia features in C2C12 cells treated with recombinant IL6. We confirmed that the induction of IL6 signalling (Extended Data Fig. 6k,l) was associated with an induction of one-carbon metabolites (Extended Data Fig. 6m) and myotube atrophy (Extended Data Fig. 6n,o), which was rescued upon FIDAS-5 treatment. FIDAS-5 also significantly reduced glucose consumption in IL6-treated cells (Extended Data Fig. 6p,q).

Finally, we assessed whether this link between one-carbon metabolism and cachexia features was restricted to muscle cells or also applied to other cell types, that is, adipocytes. Treatment of 3T3-L1 adipocytes with different doses of L-methionine, analogous to the experiment in C2C12 cells (Fig. 5), did not affect lipolysis or glucose consumption (Extended Data Fig. 6r–t), suggesting that the connection is indeed cell type specific.

Altogether, our data suggest that activation of one-carbon metabolism is an energy-consuming process that could contribute to glucose hypermetabolism and muscle atrophy in cachexia.

### Cachexia causes rewiring of glucose flux in skeletal and cardiac muscle

**Extended Data Fig. 7:**
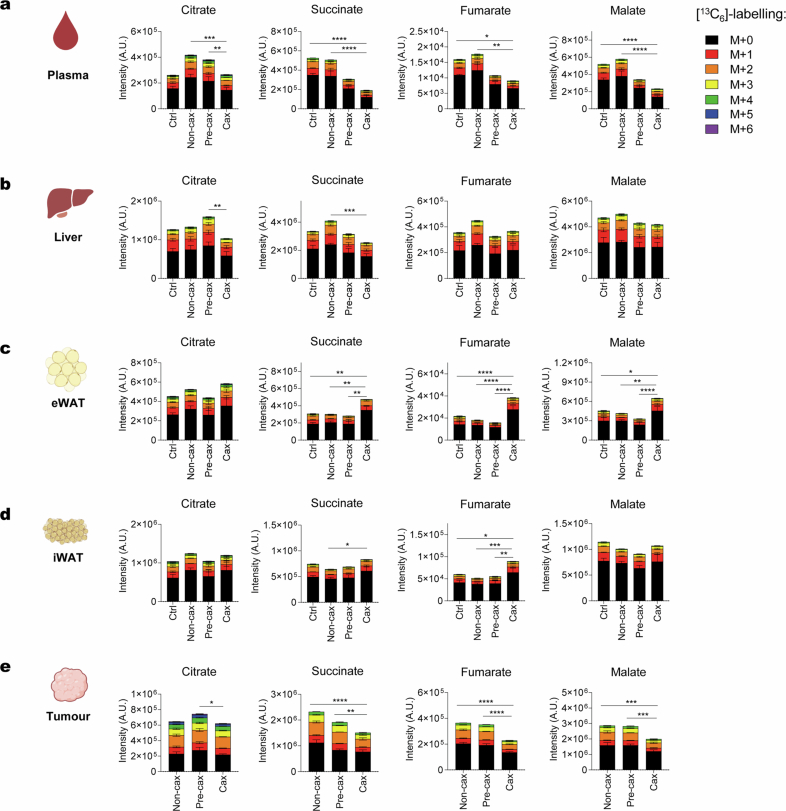
Glucose utilisation in cachexia target tissues. (**a-e**) Tracing experiment using isotopically labelled glucose ([^13^C_6_]-glucose) in healthy controls (Ctrl), non-cachectic (Non-cax), pre-cachectic (Pre-cax) and cachectic (Cax) mice with tumours. *n* = 4 animals per group. See also Fig. 1a for experimental setup. Incorporation of labelled carbons from [^13^C_6_]-glucose into metabolites of the TCA cycle in plasma (**a**), liver (**b**), eWAT (**c**), iWAT (**d**), and tumour (**e**). Unlabelled metabolites are referred as M + 0, isotopically labelled metabolites as M + X, where X represent the number of labelled carbon atoms. Data are presented as MS signal intensity (arbitrary units A.U.). Data are mean ± s.e.m. Statistical analysis: two-way ANOVA with Dunnett’s post-hoc tests *vs*. Cax. *p < 0.05, **p < 0.01, ***p < 0.001, ****p < 0.0001 compared to Cax. Source data

We next assessed alteration in glucose flux in the different tissues of cachectic mice, taking advantage of the injection of [^13^C_6_]-glucose (Fig. 1a). After 1 h, mainly intermediates of glycolysis and the TCA cycle were labelled. We observed very different responses to glucose between cachexia target tissues. In plasma and liver, total levels of TCA metabolites and labelled isotopologues were reduced, supporting previous reports showing mitochondrial dysfunction in livers of cachectic animals^29,30^ (Extended Data Fig. 7a,b). In WAT depots, total and labelled citrate levels were unchanged in C26-TB mice compared with controls, whereas unlabelled succinate and fumarate were increased. This indicates that alternative carbon sources (for example, from β-oxidation or amino acid degradation) feed the TCA cycle. However, because these cannot replenish the truly anaplerotic substrates, alternative carbon sources cannot keep TCA running alone, ultimately causing a block in the TCA cycle (Extended Data Fig. 7c,d). The low labelling of TCA intermediates also suggested the use of carbon units for alternative pathways such as de novo lipogenesis, a process increased in cachexia and contributing to whole-body wasting^31^. Cachectic C26 tumours utilised glucose for mitochondrial respiration to a lower extent compared with non-cachectic NC26 tumours (Extended Data Fig. 7e).

**Fig. 6:**
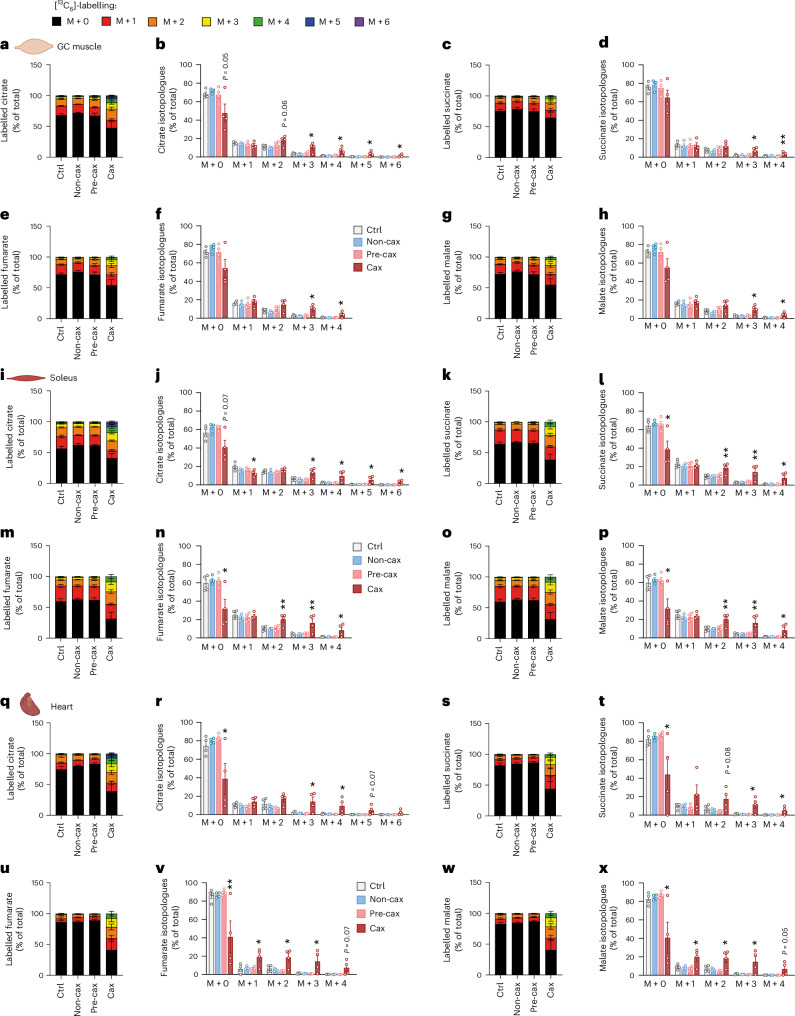
Cachexia causes rewiring of glucose flux in skeletal and cardiac muscle. Tracing experiment using isotopically labelled glucose ([^13^C_6_]-glucose) in Ctrl, Non-cax, Pre-cax and Cax mice with tumours. *n* = 4 animals per group. See also Fig. 1a for the experimental set-up, and Extended Data Figs. 7 and 8. **a**–**x**, Incorporation of labelled carbons from [^13^C_6_]-glucose into metabolites of the TCA cycle in GC muscles (**a**–**h**), soleus (**i**–**p**) and hearts (**q**–**x**). Unlabelled metabolites are referred as M + 0 and isotopically labelled metabolites as M + *X*, where *X* represents the number of labelled carbon atoms. Data are presented as the percentage of total metabolite levels. Metabolites shown are citrate (**a** and **b**, **i** and **j**, and **q** and **r**), succinate (**c** and **d**, **k** and **l**, and **s** and **t**), fumarate (**e** and **f**, **m** and **n**, and **u** and **v**) and malate (**g** and **h**, **o** and **p**, and **w** and **x**). Stacked bar graphs show the overall isotopologue levels; bar graphs show the levels of each individual isotopologue. MS signal intensity (arbitrary units, AU) can be found in Extended Data Fig. 8. Data are the mean ± s.e.m. Statistical analysis: one-way ANOVA with Dunnett’s post-hoc tests versus Ctrl. *P < 0.05, **P < 0.01 compared with Ctrl. Source data

**Extended Data Fig. 8:**
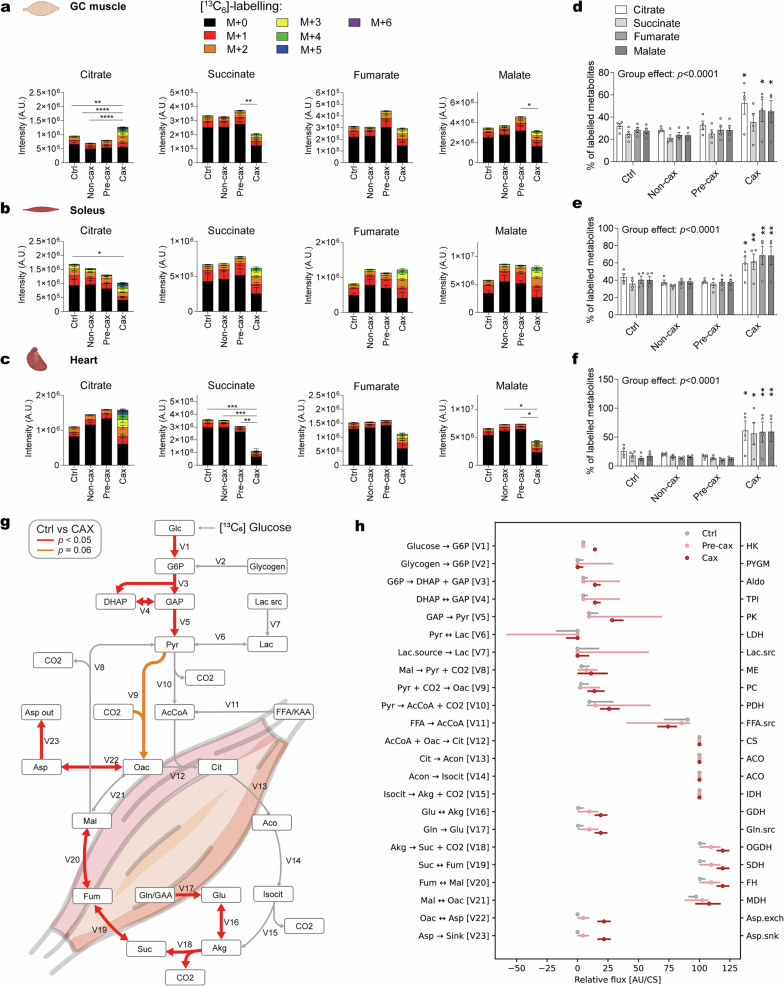
Glucose utilisation in cachectic skeletal and cardiac muscle. (**a-h**) Tracing experiment using isotopically labelled glucose ([^13^C_6_]-glucose) in healthy controls (Ctrl), non-cachectic (Non-cax), pre-cachectic (Pre-cax) and cachectic (Cax) mice with tumours. *n* = 4 animals per group. See also Fig. 1a for experimental setup and Fig. 6. (**a, b, c**) Incorporation of labelled carbons from [^13^C_6_]-glucose into metabolites of the TCA cycle in GC muscles (**a**), soleus (**b**) and hearts (**c**). Unlabelled metabolites are referred as M + 0, isotopically labelled metabolites as M + X, where X represent the number of labelled carbon atoms. Data are presented as MS signal intensity (arbitrary units A.U.). (**d, e, f**) Percentage of labelled metabolites (sum of M + X) out of total metabolites (sum of M + 0 and M + X) detected in GC muscles (**d**), soleus (**e**) and hearts (**f**). Data are mean ± s.e.m. Statistical analysis: unpaired two-way ANOVA with Dunnett’s post-hoc tests *vs*. Cax. *p < 0.05, **p < 0.01, ***p < 0.001, ****p < 0.0001 compared to Cax (a, b, c), Ctrl or Non-cax (d, e, f). (**g-h**) Modelling of the fluxes of glucose feeding the TCA cycle in GC muscles Ctrl (grey lines) and Cax mice (statistically significant red lines). For clarity, flux values are plotted separately. Significantly different fluxes are highlighted in red. (**h**) Flux relative to citrate synthase are presented as interval plots (means and 95% confidence intervals) for Ctrl (grey), Pre-cax (light red) and Cax (dark red) mice. For mathematical modelling and numerical values: please refer to Methods and Supplemental Data Table 2. Source data

Curiously, while unlabelled metabolites (representing a basal state) were unchanged or rather decreased in skeletal and cardiac muscles of Cax mice (Fig. 6, Extended Data Fig. 8a–c), probably consecutive to anorexia-induced hypoglycemia, we observed an unexpected increase in the labelling of TCA metabolites in GC muscle (Fig. 6a–h), soleus muscle (Fig. 6i–p) and heart (Fig. 6q–x) upon glucose injection. As was the case for L-methionine-treated C2C12 myotubes, the percentage of label incorporation (Fig. 6 and Extended Data Fig. 8d–f), but also the labelling of higher isotopologues (M + 3 and more) of TCA cycle metabolites suggested a rewiring of glucose flux and hypermetabolism, precisely an acceleration of glucose metabolism feeding into the TCA cycle, specifically in muscle tissues in cachexia.

Interestingly, M + 3-labelled citrate, succinate, fumarate and malate stem from the action of pyruvate carboxylase (PC). Thus, the significant increase in M + 3 labelling of these metabolites in muscles of Cax mice indicates that PC is active, providing intermediates to the TCA cycle. [1-^13^C]-pyruvate tracing in C2C12 myotubes (pyruvate labelled only on the first carbon) showed M + 1 labelling of TCA intermediates, confirming that PC is active in muscle cells (Supplementary Fig. 4). Increased M + 0 citrate and malate (derived from the non-labelled carbons of [1-^13^C] pyruvate) upon methionine treatment also indicated a hypermetabolic state in myotubes in which one-carbon metabolism was overactivated. Of note, the elevated M + 0 TCA intermediates without a proportional M + 1 increase in [1-¹³C]pyruvate-traced, methionine-treated C2C12 myotubes reflect the pyruvate dehydrogenase (PDH)-dominant flux pattern amplified by hypermetabolic demands, isotope dilution from unlabelled anaplerotic sources, and metabolic rewiring induced by one-carbon metabolism activation. Methionine treatment induced coordinated metabolic reprogramming that increased anaplerotic flux from multiple unlabelled sources to maintain TCA cycle function despite ongoing catabolism.

We next performed metabolic modelling of [^13^C]-glucose labelling data to assess the flux of different metabolite sources feeding the TCA cycle in the GC muscle of Ctrl, Pre-cax and Cax mice (Extended Data Fig. 8g,h and Supplementary Table 2). We normalised the fluxes for each group to citrate synthase activity (*V12*). Relative flux through PC and PDH was increased in GC muscle (*V9* and *V10*), indicating that more glucose enters the TCA cycle. By contrast, the relative flux filling the acetyl-CoA pool from fatty acid oxidation and ketogenic amino acids (*V11*) was decreased. We observed a significantly increased flux through 2-oxoglutarate dehydrogenase (*V18*) in cachectic muscle, and trends towards increased succinate dehydrogenase, fumarate hydratase and malate dehydrogenase (*V19–21*) activities, supporting the increase in flux at almost all reactions of the cycle. This occurs despite normalization to citrate synthase activity, which is already elevated in Cax muscle, as shown in Fig. 6a,b. Lastly, our flux analysis highlighted the significantly increased usage of glutamine (including also glucogenic amino acid backbones) as a substrate for the TCA cycle (*V16* and *V17*). The Pre-cax state showed trends towards increased flux in all above-mentioned reactions despite unchanged body weight and composition, indicating that glucose hypermetabolism in muscle may be an early event contributing to cachexia (Extended Data Fig. 8h).

In summary, our data revealed clear tissue-specific alterations in glucose usage in cachexia, especially an unexpected hypermetabolism of skeletal and cardiac muscles upon nutrient availability, which could contribute to energy loss in cachexia.

### Activation of one-carbon metabolism is a unifying feature of cancer cachexia in mice

**Fig. 7:**
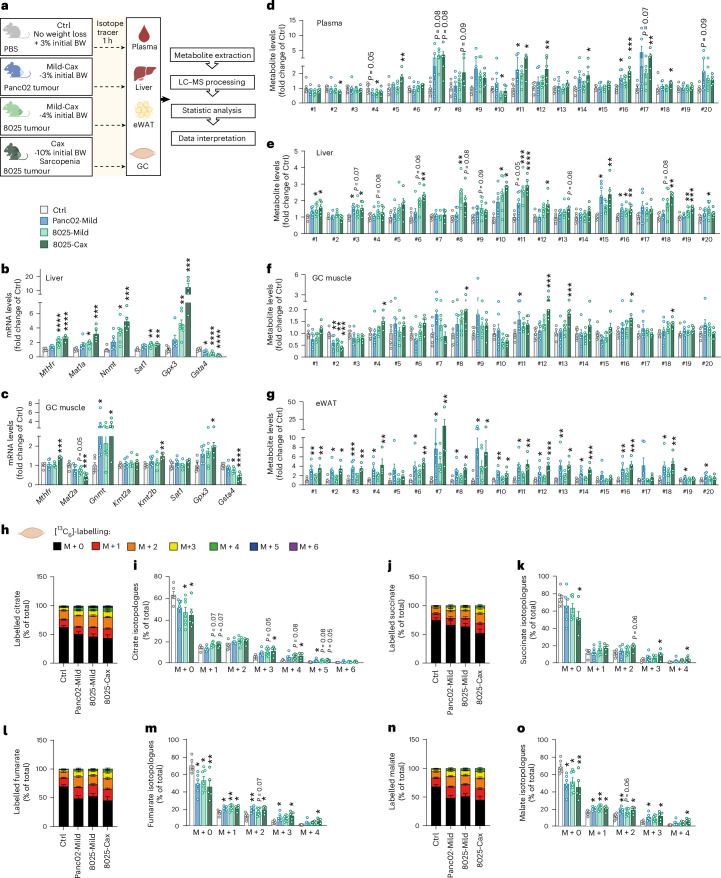
Activation of one-carbon metabolism is a unifying feature of cancer cachexia in mice. **a**, Experimental set-up. Mice were divided into four groups: mice injected with PBS, healthy controls, no tumour (Ctrl, grey, no weight loss, *n* = 6 animals per group); mice orthotopically injected with Panc02 pancreatic cancer cells, mild cachexia (Panc02-Mild, blue, mean BW loss of 3%, *n* = 6 animals per group); mice orthotopically injected with 8025 pancreatic cancer cells divided into two groups, mild cachexia (8025-Mild, light green, mean body weight loss of 4%, *n* = 7 animals per group) and cachexia (8025-Cax, dark green, mean body weight loss of 10%, sarcopenia, *n* = 5 animals per group). On the day of euthanasia, mice were fasted for 6 h and injected with an isotopic tracer ([^13^C_6_]-glucose). Tissues (plasma, liver, eWAT and GC muscle) were collected exactly 1 h later. Tissues were then processed for tracer metabolomics. See also Extended Data Fig. 9. **b**,**c**, Relative mRNA expression levels of key enzymes of one-carbon metabolism and related pathways in liver (**b**) and GC muscle (**c**). **d**–**g**, Metabolite levels of one-carbon metabolism and related pathways in plasma (**d**), liver (**e**), GC muscle (**f**) and eWAT (**g**). Metabolite IDs as in the list in Fig. 3b. Statistical analysis based on raw data (2^−ΔCt^ values and MS signal intensities, arbitrary units (AU)). **h**–**o**, Incorporation of labelled carbons from [^13^C_6_]-glucose into metabolites of the TCA cycle in GC muscles. Unlabelled metabolites are referred as M + 0 and isotopically labelled metabolites as M + *X*, where *X* represents the number of labelled carbon atoms. Data are presented as the percentage of total metabolite levels. Stacked bar graphs (**h**, **j**, **l** and **n**) show the overall isotopologue levels; bar graphs (**i**, **k**, **m** and **o**) show the levels of each individual isotopologue (citrate (**h** and **i**), succinate (**j** and **k**), fumarate (**l** and **m**) and malate (**n** and **o**)). Data are the mean ± s.e.m. Statistical analysis: one-way ANOVA with Dunnett’s post-hoc tests versus Ctrl or Kruskal–Wallis with Dunn’s post-hoc tests versus Ctrl. *P < 0.05, **P < 0.01, ***P < 0.001, ****P < 0.0001 compared with Ctrl. Source data

**Extended Data Fig. 9:**
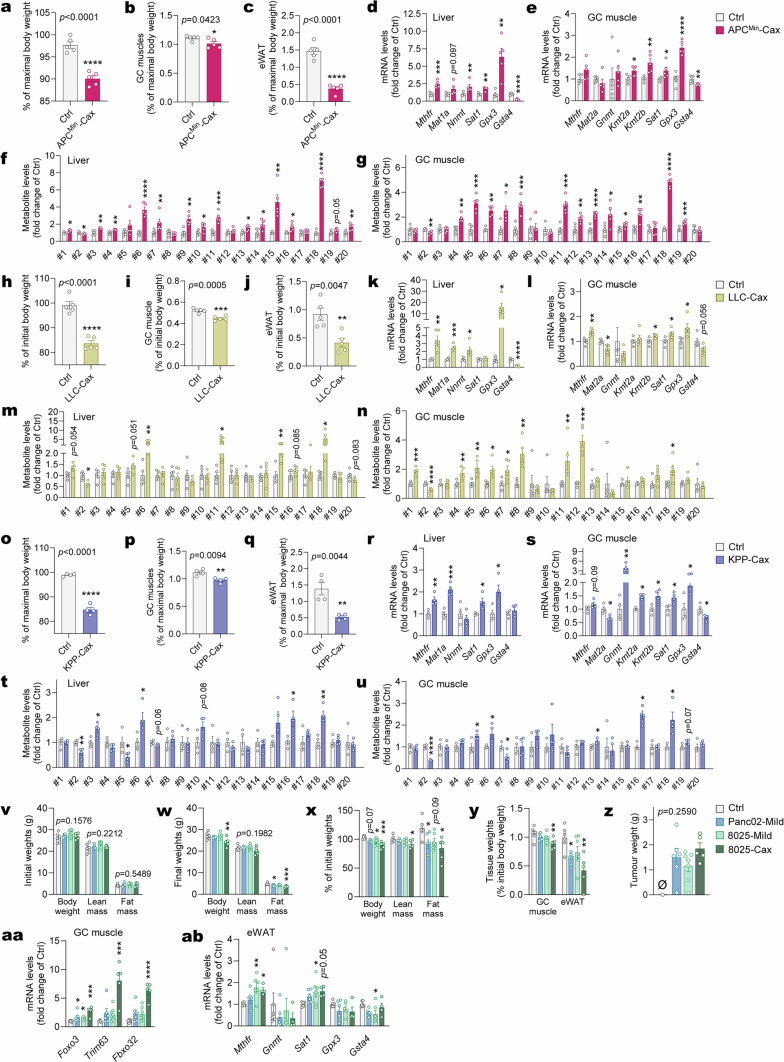
One-carbon metabolism activation across 5 different cancer cachexia mouse models. (**a-g**) Healthy controls (wildtype, Ctrl, grey) and cachectic APC^Min^ mutant mice (intestinal cancer, pink). Mice were fasted for 6 h before tissue sampling. *n* = *5* animals per group. (**h-n**) Healthy controls (PBS-injected, Ctrl, grey) and cachectic LLC tumour-bearing mice (lung carcinoma, green). Tissue sampling in random-fed animals. *n* = *5* animals per group. (**o-u**) Healthy controls (wildtype, Ctrl, grey) and cachectic KPP mutant mice (pancreatic ductal adenocarcinoma, violet). Tissue sampling in random-fed animals. *n* = *4* animals per group. (**a-c, h-j, o-q**) Body weight loss expressed as percentage of initial or maximal body weight (**a, h, o**); final tissue weights: GC muscle(s) (**b, i, p**) and eWAT (**c, j, q**). (**d-e, k-l, r-s**) Relative mRNA expression level of key enzymes of one-carbon metabolism and related pathways in liver (**d, k, r**) and GC muscle (**e, l, s**). (**f-g, m-n, t-u**) Metabolite levels of one-carbon metabolism and related pathways in liver (**f, m, t**) and GC muscle (**g, n, u**). Metabolites are similar to the list presented in Fig. 3b. (**v-ab**) See also Fig. 7a for experimental setup. Mice were divided into 4 groups: mice injected with PBS, healthy controls, no tumour (Ctrl, grey, *n* = 6 animals per group); mice orthotopically injected with Panc02 pancreatic cancer cells, mild cachexia (Panc02-Mild, blue, *n* = 6 animals per group); mice orthotopically injected with 8025 pancreatic cancer cells divided into two groups, mild cachexia (8025-Mild, light green, *n* = 7 animals per group) and cachexia (8025-Cax, dark green, *n* = 5 animals per group). (**v-w**) Initial (**v**) and final (**w**) body, lean and fat mass. (**x**) Loss of body, lean and fat mass expressed as percentage of initial mass. (**y**) Final tissue weights (GC muscle, eWAT). (**z**) Final tumour weights. (**aa**) Relative mRNA expression of atrogens in GC muscle. (**ab**) Relative mRNA expression of key enzymes of one-carbon metabolism and related pathways in eWAT. Data expressed as fold change of Ctrl. Statistical analysis on raw data (2^−ΔCt^ values and MS signal intensities, arbitrary units A.U.). Data are mean ± s.e.m. Statistical analysis: two-tailed, non-adjusted, Student’s *t* test or Mann and Whitney’s test (**a**-**u**), unpaired one-way ANOVA with Dunnett’s post-hoc tests or Kruskal Wallis with Dunn’s post-hoc tests *vs*. Ctrl (**v**-**ab**). *p < 0.05, **p < 0.01, ***p < 0.001, ****p < 0.0001 compared to Ctrl animals. Source data

To assess whether the described multi-organ changes in one-carbon metabolism are specific to the C26 mouse model or a more general feature of cancer cachexia, we assessed the gene expression and metabolite profiles of liver and muscle in five additional mouse models: Apc^Min^ (ref. ^8^) (genetic model developing intestinal polyps and cachexia over a few months; Extended Data Fig. 9a–g), Lewis lung carcinoma^8^ (LLC, heterotopic implantation, cachexia within a few weeks; Extended Data Fig. 9h–n), KPP^32^ (genetic model with inducible pancreatic cancer, cachexia development over a few months; Extended Data Fig. 9o–u), Panc02 (ref. ^33^) (pancreatic ductal adenocarcinoma (PDAC), orthotopic implantation, mild cachexia within a few weeks; Fig. 7 and Extended Data Fig. 9v–ab) and 8025 (ref. ^3^) (PDAC, orthotopic implantation, cachexia within a few weeks; Fig. 7 and Extended Data Fig. 9v–ab). Body weights and tissue masses corresponding to these five models are shown in Extended Data Fig. 9a–c,h–j,o–q,v–z. Panc02 cells induced mild cachexia, reflected in 3% body weight loss and a mild induction in gene expression of muscle atrophy markers (Fig. 7a and Extended Data Fig. 9x,y,aa). The 8025 cells induced more severe cachexia, and we investigated mice at two timepoints to represent mild cachexia (4% weight loss, mild induction of muscle atrophy markers) and cachexia (10% weight loss, sarcopenia), respectively (Fig. 7a and Extended Data Fig. 9x,y,aa).

Expression of genes encoding for enzymes related to one-carbon metabolism, representing different arms of the pathway (Figs. 3a and 4c–f), were uniformly altered in cachexia target tissues (liver, muscle and/or adipose tissue) of the different models (Fig. 7b,c and Extended Data Fig. 9d,e,k,l,r,s,ab) and were comparable to the gene expression analysis shown in Fig. 4. These include genes encoding for key methyltransferases such as *Nnmt*, glycine *N*-methyltransferase (*Gnmt*) (conversion of glycine into sarcosine), *Kmt2a*, *Kmt2b*, methylenetetrahydrofolate reductase (*Mthfr*) (key step of folate cycle, conversion of 5,10-methyleneTHF to 5-methylTHF), methionine adenosyltransferases (*Mat1a* and *Mat12a*) (conversion of methionine to SAM), *Sat1*, *Gpx3* and *Gsta4*. Once again, such analyses confirmed tissues’ specificity regarding key methyltransferases in cachexia (for example, *Nnmt* in liver and *Kmt*(*s*) in muscle).

As in the C26 model, we also observed an accumulation of metabolites associated with elevated one-carbon metabolism (Fig. 7d–g and Extended Data Fig. 9) (for example, #9 sarcosine, #10 MNAM, #11 di- and #12 tri-methyllysines and #18 thymidine) in the liver and muscle of cachectic animals compared with their respective controls (Fig. 7e,f and Extended Data Fig. 9f,g,m,n,t,u). In PDAC models, we also observed similar alterations in one-carbon-related metabolites in plasma and adipose tissue (Fig. 7d,g). Induction of one-carbon metabolism in the PDAC experiment was gradual with cachexia severity, with Panc02-Mild and 8025-Mild groups exhibiting gene and metabolite patterns in-between healthy controls and 8025-Cax (Fig. 7b–g and Extended Data Fig. 9ab). Of note, differences in the amplitude of the regulation of individual metabolites and genes between the various mouse experiments may be explained by several factors such as tumour entity, mouse strain, feeding status or experimental set-up. While C26, Panc02, 8025 and APC^Min^ cohorts were fasted for 6 h, optimised for metabolic studies, LLC and KPP experiments were not specifically designed for metabolic assays and were necropsied in random-fed conditions. Nevertheless, the conserved alteration in gene expression and metabolites in six independent mouse models highlights the tissue-overarching activation of one-carbon metabolism as a hallmark of cachexia, associated with different tumour entities and cachexia trajectories.

Linking the activation of one-carbon metabolism with muscle glucose hypermetabolism, we also investigated [^13^C_6_]-glucose tracing in GC muscle of Panc02 and 8025 tumour bearing mice (Fig. 7a,h–o). ^13^C labelling showed an overall enrichment of labelled TCA cycle metabolites in PDAC mice compared with Ctrl, increasing gradually with cachexia severity. Tracing isotopologues also confirmed the enrichment of highly labelled isotopologues (M + 4 or higher), particularly in 8025-Cax mice, supporting the notion of increased glucose flux through the TCA cycle in cachectic muscle.

In conclusion, the PDAC experiments (representing a different genetic background, tumour entity and laboratory environment) recapitulated our main findings from the C26 model regarding one-carbon metabolism and glucose hypermetabolism.

### Activation of one-carbon metabolism associated with muscle hypermetabolism is also a feature of cachexia in the humanised SW480-tumour mouse model

**Fig. 8:**
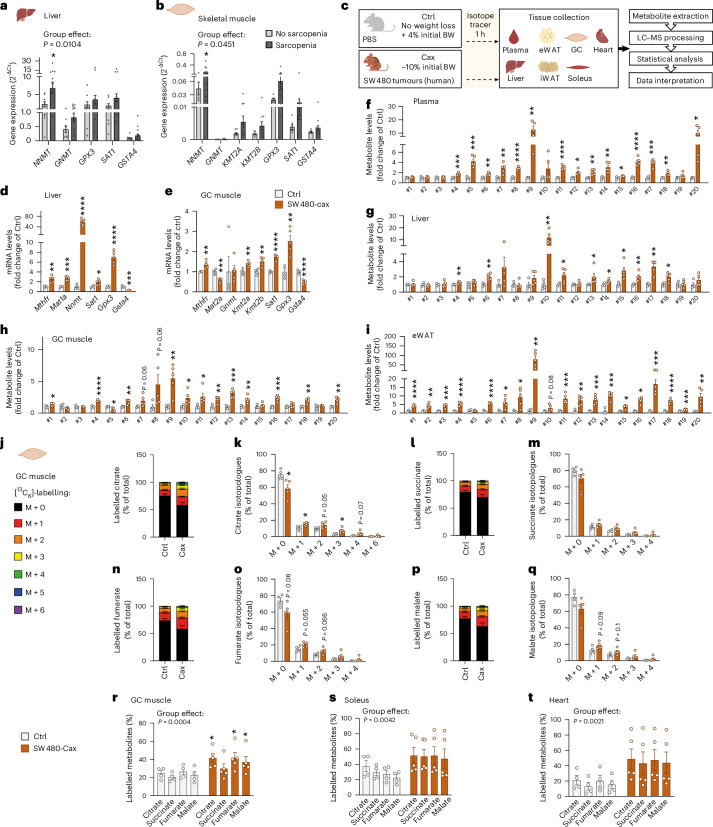
Activation of one-carbon metabolism associated with muscle hypermetabolism is also a feature of cachexia in the humanised SW480-tumour mouse model. **a**,**b**, Relative mRNA expression level of key enzymes of one-carbon metabolism and related pathways in liver (**a**) and skeletal muscle (**b**) of patients with cancer with or without sarcopenia (no sarcopenia: *n* = 9 liver samples, 7 muscle samples; sarcopenia, *n* = 19 liver samples, 17 muscle samples). See also Supplementary Table 3 for patients’ clinical data. **c**, Experimental set-up. Mice were injected subcutaneously either with PBS (healthy control, no tumour, Ctrl, grey, *n* = 4 animals) or with the cachexia-inducing SW480 cancer cells (human colon carcinoma, cachexia, Cax, dark orange, mean body weight loss of 10%, *n* = 5 animals). On the day of euthanasia, mice were fasted for 6 h and injected with the isotopic tracer [^13^C_6_]-glucose. Tissues (plasma, liver, eWAT, iWAT, heart, GC and soleus muscles) were collected exactly 1 h later. Tissues were then processed for tracer metabolomics. See also Extended Data Fig. 10. **d**,**e**, Relative mRNA expression level of key enzymes of one-carbon metabolism and related pathways in liver (**d**) and GC muscle (**e**). **f**–**i**, Metabolite levels of one-carbon metabolism and related pathways in plasma (**f**), liver (**g**), GC muscle (**h**) and eWAT (**i**). Metabolite IDs as in the list in Fig. 3b. Statistical analysis on raw data (2^−ΔCt^ values and MS signal intensities, arbitrary units (AU)). **j**–**q**, Incorporation of labelled carbons from [^13^C_6_]-glucose into metabolites of the TCA cycle in GC muscles. Unlabelled metabolites are referred as M + 0 and isotopically labelled metabolites as M + *X*, where *X* represents the number of labelled carbon atoms. Data are presented as the percentage of total metabolite levels. Stacked bar graphs (**j**, **l**, **n** and **p**) show the overall isotopologue levels; bar graphs (**k**, **m**, **o** and **q**) show the levels of each individual isotopologue (citrate (**j** and **k**), succinate (**l** and **m**), fumarate (**n** and **o**) and malate (**p** and **q**)). **r**–**t**, Distribution of all labelled TCA metabolite isotopologues detected in GC muscle (**r**), soleus (**s**) and heart (**t**). Data are the mean ± s.e.m. Statistical analysis: two-tailed, non-adjusted, Student’s *t*-test or Mann–Whitney test (**d**–**i**, **k**, **m**, **o** and **q**), two-way ANOVA with Šidák’s (**a**, **b** and **r**–**t**) post-hoc tests. *P < 0.05, **P < 0.01, ***P < 0.001, ****P < 0.0001 versus Ctrl or no sarcopenia groups. Source data

To verify the relevance of our findings for patients, we first checked the expression of key enzymes related to one-carbon metabolism in liver and skeletal muscle from patients with cancer with or without sarcopenia (Fig. 8a,b and Supplementary Table 3). Sarcopenia was associated with increased gene expression of *NNMT* and increased expression of the signature gene set overall, in accordance with our previous observation linking this pathway to muscle wasting in different mouse models.

**Extended Data Fig. 10:**
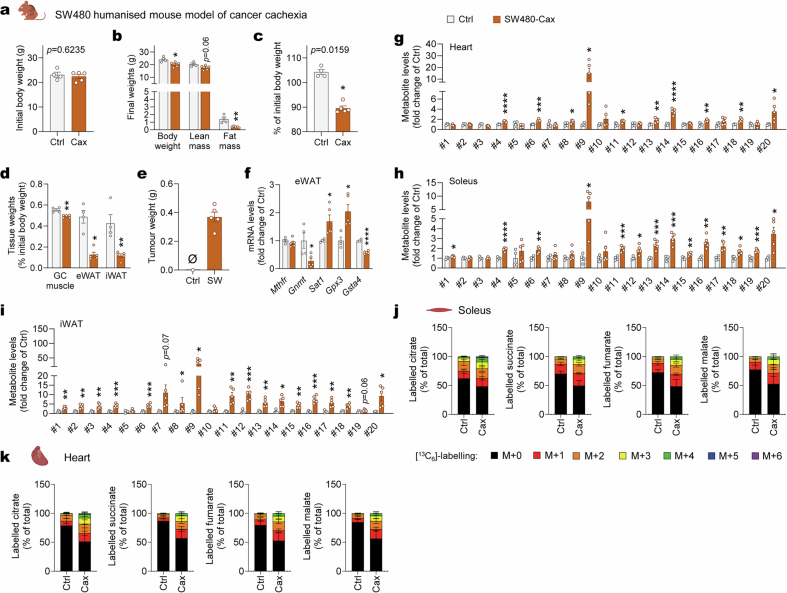
One-carbon metabolism activation in the humanised SW480 model. (**a-h**) Tracing experiment using isotopically labelled glucose ([^13^C_6_]-glucose) in healthy controls (Ctrl, grey, *n* = 4 animals) or cachectic SW480 tumour mice (human colon carcinoma, Cax, dark orange, *n* = 5 animals). See also Fig. 8c for experimental setup. (**a**) Initial body weight. (**b**) Final body, lean and fat mass. (**c**) Body weight loss expressed as percentage of initial body weight. (**d**) Final tissue weights (GC muscle, eWAT, iWAT). (**e**) Final tumour weight. (**f**) Relative mRNA expression level of key enzymes of one-carbon metabolism and related pathways in eWAT from Ctrl and Cax mice. Data expressed as fold change of Ctrl. (**g-i**) Metabolite levels of one-carbon metabolism and related pathways in heart (**g**), soleus (**h**) and iWAT (**i**). Metabolites are similar to the list presented in Fig. 3b. Statistical analysis on raw data (2^−ΔCt^ values and MS signal intensities, arbitrary units A.U.). (**j-k**) Incorporation of labelled carbons from [^13^C_6_]-glucose into metabolites of the TCA cycle in soleus muscles (**j**) and heart (**k**). Unlabelled metabolites are referred as M + 0, isotopically labelled metabolites as M + X, where X represents the number of labelled carbon atoms. Data are presented as % of total metabolite levels. Data are mean ± s.e.m. Statistical analysis: two-tailed, non-adjusted, Student’s *t* test or Mann and Whitney’s test (**a**-**i**). *p < 0.05, **p < 0.01, ***p < 0.001, ****p < 0.0001. Source data

We next performed metabolomics and [^13^C_6_]-glucose tracing in a humanised cachexia model (human SW480 colon carcinoma) (Fig. 8c). Tumour-bearing mice developed a clear cachectic phenotype, with a significant reduction in final body weight and tissue mass (Extended Data Fig. 10a–e). Consistent with findings from other mouse models of cachexia and from patients, gene expression of enzymes related to one-carbon metabolism was significantly changed in liver, muscle and adipose tissue of cachectic SW480 animals compared with Ctrl (Fig. 8d,e and Extended Data Fig. 10f). Furthermore, we observed a highly significant induction of metabolites related to one-carbon metabolism in all tissues from these animals, including plasma, liver, heart, skeletal muscles and adipose tissues (Fig. 8f–i and Extended Data Fig. 10g–i).

Likewise, while unlabelled TCA cycle metabolite levels in cardiac and skeletal muscles were mostly decreased or unchanged in the SW480 cachectic condition, labelled TCA cycle metabolites were enriched (Fig. 8j–t and Extended Data Fig. 10j,k), indicating higher glucose flux through the TCA cycle, comparable to the C26 and PDAC models.

Overall, our data highlight the existence of a spatio-temporally coordinated response across multiple cachexia target tissues, characterised by the activation of the one-carbon metabolism, which is conserved between multiple different models of murine and human cancer. Activation of one-carbon metabolism is associated with glucose hypermetabolism in cachectic muscle in independent models and potentially contributes to muscle wasting in cachexia.

## Discussion

Cachexia is a wasting disorder characterised by a progressive metabolic reprogramming in multiple tissues, yet only limited data on multi-tissue responses to cachexia during disease progression have been available so far. Our study provides a comprehensive map of the temporal distribution of metabolites during disease progression across eight tissues, representing a valuable resource for future studies investigating coordinated metabolic responses to cancer. As proof of principle for the application of multi-omics data integration, we uncover and describe a coordinated response of host tissues to the presence of a cachexia-inducing tumour. In accordance with previous studies on serum and plasma metabolomics from cachectic mice^5,10–13,15,16^ and patients^6,34^ with cancer, we observed reduced levels of amino acids and energy metabolites as typical features of cachexia, in line with a catabolic condition^1^.

By combining data from metabolomics and transcriptomics from multiple cachexia target tissues, we identified one-carbon metabolism as a tissue-overarching metabolic pathway associated with wasting across all host tissues we assessed, in both murine and humanised settings, confirming the relevance for patients with cancer. Of note, while several previous studies have identified individual hits from our study both on metabolomics and transcriptomics level (see comparative analyses with references in Supplementary Tables 4 and 5), only the integration of multiple -omics datasets ultimately allowed the conclusion of a coordinated response. While we have used exclusively male mice, previous studies on female mice (C26 and 4T1 tumour models^10,24^) have reported common regulation of some one-carbon metabolites and genes, such as sarcosine, dimethylglycine and *Nnmt*.

One-carbon metabolism is essential for a multitude of cellular processes, including nucleic acid and protein metabolism, methylation, redox balance, energy metabolism and growth^22^. Methylation reactions, particularly DNA, RNA and histone methylation^35^, are directly linked to the regulation of gene expression and, hence, can elicit longer-lasting transcriptional adaptations to metabolic demands, as in the wide-reaching transcriptional reprogramming of cachexia target tissues described herein. Furthermore, methylated products of the one-carbon metabolism (for example, 1-methyladenosine) have previously been linked to the modulation of glucose metabolism in the context of cancer^36^ (metabolite #13 in Figs. 3, 7 and 8 and Extended Data Fig. 4).

Due to the multitude of tasks, it is difficult to speculate on the cause or consequence of the tissue-overarching activation of one-carbon metabolism in cachexia, and further studies will need to address these specifically. One possible explanation lies in tumour-mediated reprogramming: the decrease in essential metabolites required for tumour growth, coupled with a simultaneous increase in multiple one-carbon metabolism intermediates in host tissues, may serve to supply the tumour with necessary building blocks. The tumour’s dependency on components produced by one-carbon metabolism is highlighted by the frequent use of chemotherapeutics targeting this pathway, such as methotrexate, raltitrexed or 5-fluorouracil^37^. Of note, these treatments aggravate cachexia^38^. The liver is a primary site of one-carbon metabolism and is responsible for amino acid catabolism and phosphatidylcholine synthesis^39^. In light of enhanced protein degradation in cachexia^40^, both products of protein catabolism and the hypermetabolic state producing many one-carbon metabolic intermediates may enter liver one-carbon metabolism for detoxification through methylation reactions (for example, MNAM and phosphatidylcholines). Linking one-carbon cycle and systemic energy homeostasis, creatine serves as an essential energy and pH buffer, and its synthesis accounts for approximately 50% of methylation flux in the liver^41^. Creatine levels are increased threefold in livers of cachectic mice (Supplementary Table 1). Adipose tissues primarily use one-carbon metabolism for (phospho-)lipid remodelling^42^, whereas the pathway’s specific role in muscle is less clear so far^22^. A recent study has shown that the methionine cycle controls DNA methylation in muscle of cachectic mice, but to which extent this contributes to the total methylation flux in this tissue is unclear as other methylated metabolites are increased in Cax mice^43^. Our in-depth metabolomics and gene expression profiling suggest that one-carbon metabolites are used for purposes other than DNA methylation in cachectic settings, that is, for redox processes, folate cycle, pyrimidine synthesis or epigenetic modification at the RNA level. A redirection of one-carbon metabolites occurs, potentially driving metabolites away from a more ‘long-term’ regulation, such as DNA epigenetics, to more immediately urgent processes, such as redox regulation or nucleotide synthesis, in times of enormous stress to the cells.

A recent study investigated individual key nodes of one-carbon metabolism in the cancer setting^44^, showing the potential of targeting this pathway for cachexia. Methionine is an essential cog of one-carbon metabolism, and dietary methionine restriction is effective in extending lifespan^45,46^ and improving adiposity, insulin signalling and energy expenditure^45^. This may also be relevant for cachexia, as an early study in a small patient cohort reported an increased demand for active one-carbon units, which was positively correlated with resting energy expenditure and weight loss before chemotherapy and cancer diagnosis^47^. Meanwhile, methionine or SAM supplementation has been shown recently to partially rescue muscle loss in cachectic mice with cancer^43^. This highlights a complex interaction between methionine demand and energy homeostasis in physiological and pathological conditions.

An upregulation of the one-carbon metabolism has previously been described as an early response to mitochondrial dysfunction^48,49^. Vice versa, one-carbon cycle activity influences mitochondrial metabolism^50^. In light of this, the observed glucose hypermetabolism in myotubes following methionine supplementation (and its reversal by FIDAS-5) aligns with known metabolic disruptions in cachexia. Our studies further show that hypermetabolism was linked to myotube atrophy both in vitro and in vivo in cachectic muscle. It is conceivable that muscle hypermetabolism contributes to systemic wasting, considering that essential processes to sustain cellular and organ function determine resting energy expenditure. While alterations in total levels of TCA metabolites in cachectic muscle have previously been noted^14,17^, glucose tracing has allowed us to accurately quantify the contributions of various metabolites to the TCA cycle, with muscle utilising both glucose and glutamine as substrates. As glucose became overly abundant upon injection 1 h before necropsy in our experimental setting, we can speculate that amino acid feeding of the TCA cycle for energy production is a contributor to muscle wasting in the basal state under hypoglycaemia. Of note, hypermetabolism in cachectic muscle has previously been described in the context of burn-induced cachexia^51,52^.

In summary, we describe a spatio-temporal metabolite network specific to cachexia. Integrating data from multi-omics and metabolite tracing identified key regulatory nodes linked to metabolic wasting, in both preclinical and clinical settings, and highlighted a coordinated response to wasting across tissues. This distinctive, systems-level perspective on the organization of tissue metabolism during disease progression could uncover overlooked metabolic connections, offering possibilities for therapeutic developments fighting cachexia. Our data represent an important resource for future studies investigating systemic metabolic reprogramming in cancer.

## Methods

Our research complies with all relevant ethical regulations as outlined in the ‘Animal experiments’ and ‘Clinical study’ sections.

### Animal experiments

Mice were maintained on a 12-h light–dark cycle at 22 °C and 40–50% humidity unless stated otherwise. Mice had ad libitum access to water and rodent chow as specified for the individual experiments. Humane endpoints for animal studies were tumour >1.5 cm or tumour ulceration. Maximal burden was never exceeded.

### Tracer experiments

#### C26 and SW480 models

In vivo experiments were carried out in 9.5- to 11-week-old male BALB/c or FoxChase SCID mice (Charles River Laboratories) under specific pathogen-free conditions. Rodent chow was Kliba Nafag #3437, Promivi Kliba AG. In each experiment, mice were assigned to groups so that body weight, lean and fat mass were similar between the groups as confirmed by non-significant statistical analysis. Studies were performed in accordance with the institutional animal welfare officer and licences from the state ethics committee and government of Upper Bavaria (ROB-55.2-2532.Vet_02-18-93 and ROB-55.2-2532.Vet_02-22-47).

Mice were injected subcutaneously into the right flank with 1 million NC26 (mouse colon carcinoma, gift from M. Schweiger, Graz), 1 million C26 (mouse colon carcinoma, Deutsches Krebsforschungszentrum (DKFZ) Tumorbank) or 5 million SW480 cells (human colon carcinoma, ATCC #CCL-228, DKFZ Tumorbank) resuspended in Dulbecco’s PBS (Thermo Fisher #14190250). Non-tumour-bearing control mice were injected with PBS. Mice were monitored for 2–4 weeks after cell implantation with daily assessment of tumour size, body weight and body condition score. Mice were euthanised once they developed cachexia (body weight loss of 5–10% from initial body weight) or reached a humane endpoint. Non-cachectic NC26 or pre-cachectic C26 tumour mice with no weight loss were matched with C26 mice for tumour sizes. Body composition was assessed by EchoMRI on the day of necropsy. Mice were fasted for 6 h before being injected with a bolus of 2 g kg^−1^ of the isotopic [^13^C_6_]-glucose tracer (that is, all carbons labelled, Cambridge Isotopes #CLM-1396). Fifteen minutes after the first dose, mice were injected with a second bolus of [^13^C_6_]-glucose. These two-step glucose injections allowed sufficient metabolite enrichment and prevented system saturation by providing tissues with enough time to initiate glucose clearance before the second dose. Mice were necropsied 1 h after the first glucose injection by cervical dislocation, and the following tissues were collected in this order: blood, liver, eWAT, heart, GC muscle, soleus muscle, iWAT and tumour. Tissues for metabolomics were immediately snap-frozen in liquid nitrogen. One fat pad/depot and one GC were weighed before being frozen and used for mRNA analysis. Blood was transferred into EDTA-coated tubes, centrifuged at 2,000*g*, 4 °C for 10 min, aliquoted and snap-frozen. Samples were stored at −80 °C until further processing.

#### Panc02 and 8025 models

In vivo experiments were carried out in 11-week-old male C57BL/6J single-housed mice (Charles River Laboratories). Rodent chow was ssniff #V1536. Group assignment was performed as described above. Experiments were in accordance with experimental project AVCR 4914/2023 SOV II and approved by the Animal Care and Use Committee of the Institute of Physiology of the Czech Academy of Sciences and the Resort Professional Commission of the CAS for Approval of Projects of Experiments on Animals.

Mice were orthotopically implanted with two different pancreatic cancer cell lines: 8025 (ref. ^3^) (gift from D. Saur, Munich) and Panc02 (ref. ^33^) (gift from J. Zenka, University of South Bohemia). In brief, 3 × 10^3^ 8025 cells and 2 × 10^5^ Panc02 cells were injected in PBS:Matrigel 1:1 or warm Dulbecco’s modified Eagle medium (DMEM), respectively, into the pancreas using 30 G needles under anaesthesia. Control mice were injected with a similar amount of PBS:Matrigel. Mouse monitoring, euthanasia, glucose tracing and necropsy were performed as described above, except that body composition was assessed by dual-energy X-ray absorptiometry. Panc02 and 8025 tumour mice with mild cachexia were matched to have similar tumour sizes as cachectic 8025 tumour mice.

### APC^Min^, LLC and KPP experiments

Experiments were conducted in the same housing conditions and under the same legal regulations as described for the C26 experiment (approved in licences ROB-55.2-2532.Vet_02-18-93 and ROB-55.2-2532.Vet_02-22-47) and included only male mice.

APC^Min^/J mice, as originally described by Moser et al.^53^ and later established as a model of cachexia^54^, were purchased from Jackson Laboratory (JAX #002020) and bred on a C57BL/6J background. Mice were fasted for 6 h before necropsy and euthanised by ketamine–xylazine overdose. Samples used for metabolomics in this study were part of an experiment previously published^8^.

Twenty-week-old C57BL6/J male mice (Charles River Laboratories) were injected with 1.5 million LLC cells (ATCC #CRL-1642) in PBS, subcutaneously into the right flanks. Control mice were injected with PBS. Mouse monitoring and endpoints were as described above. Animals were necropsied by cervical dislocation in a random-fed state.

KPP founder mice KrasLSL-G12D/+, Pten flox/flox and Ptf1a Cre-ER/+, Pten flox/flox were a gift from Denis C. Guttridge^32^. Mice for experiments were bred in-house at Helmholtz Munich in accordance with all the legal authorities previously mentioned and under the licence ROB-55.2-2532.Vet_02-22-47. Mice were genotyped following the Jackson Laboratory protocol (strain 033964). Mice with genotype KrasLSL-G12D/+, Ptenflox/flox, Ptf1aCre-ER/+ were added to the experiment, and littermate control mice genotype included Kras+/+, Ptenflox/flox, Ptf1a+/+; KrasLSL-G12D/+, Ptenflox/flox, Ptf1a+/+; Kras+/+, Ptenflox/flox, Ptf1aCre-ER/+. At 26 weeks of age, KPP and control mice were injected intraperitoneally once daily for five consecutive days with 2 mg tamoxifen in 100 µl of MIGLYOL 812 Hüls Neutralöl. Mice were weighed weekly and observed more frequently when they started to lose weight. Once mice developed cachexia (weight loss >15% of their maximal body weight, humane endpoint), KPP and control mice were necropsied by cervical dislocation in random-fed conditions.

### C26 experiment with IL6-neutralising antibody

Male CD2F1 mice (7 weeks old, Charles River Laboratories) under specific pathogen-free conditions were fed an irradiated chow diet (AO4-10, Safe). Either a saline solution or C26 cells (1 million cells in 0.1 ml saline) were subcutaneously injected. Food intake and body weight were recorded. Eight mice were randomly assigned to four groups based on their body weight on the day of cell injection: Ctrl (sham-injected), C26 (C26 cancer cells and treated with PBS), C26 + IL6-nAB group (C26 cancer cells and treated with 300 μg monoclonal rat anti-murine IL6 antibody (clone MP5-20F3, BioXCell)) and C26 + IgG group (C26 cancer cells and treated with 300 μg rat IgG1 isotype control (cat. no. BE0088, BioXCell)). Treatments consisted of subcutaneous injections on days 7 and 9 after cancer cell administration, as previously described^25^. Experiment were approved by and performed in accordance with the guidelines of the local ethics committee from the UCLouvain, Belgium. Housing conditions were as specified by the Belgian Law of 29 May 2013, regarding the protection of laboratory animals.

### C26 experiment with *Il6* deletion in tumours

For CRISPR–Cas9-mediated *Il6* gene deletion in C26 cells, the following guide (g)RNAs were used: Fw: 5′CACCGAATGATGGATGCTACCAAAC3′; Rev: 5′AAACGTTTGGTAGCATCCATCATTC3′. After annealing, gRNAs were ligated with the vector pSpCas9(BB)-2A-Puro (PX459) V2.0 (Addgene #62988) using T4 ligase (NEB #M0202L). The construct was amplified in *Escherichia coli* XL10 competent cells (NEB #C2987), and plasmid isolation was performed using the NucleoBond Xtra plasmid purification kit (Marchery Nagel #740410.50) according to the manufacturer’s protocol. C26 cells were transfected with recombinant vectors and a control vector comprising scrambled RNA using Lipofectamine 3000 (Invitrogen #L3000008). Transfected cells were selected using 6 µg ml^−1^ puromycin (Sigma-Aldrich #P8833), 100 cells per 10-cm dish were seeded and single cell colonies were picked for further analysis. For verification of gene editing, cellular DNA was obtained by incubating the cells in lysis buffer (Qiagen #102-T) containing 1.5 µl Proteinase K (Roth #7528.2) at 56 °C and 1,500 rpm for 1 h, Proteinase K was subsequently denatured at 85 °C for 45 min. The suspension was centrifuged at 16,000*g* for 10 min, and the supernatant was collected for PCR. PCR was performed using Q5 high-fidelity DNA polymerase (NEB #M0491) and the following primer pairs: Fw: 5′TGGTAATCTTTTGCAGTTGTGC3′; Rev: 5′AGCTACTGCAGGCCAGTTACA3′ The amplified DNA was sent to Microsynth AG for Sanger sequencing.

IL6 deletion from cancer cells was verified using an enzyme-linked immunosorbent assay from C26 cell culture supernatants upon treatment with 100 ng ml^−1^ lipopolysaccharide for 24 h. To assess IL6 protein levels in tumour tissues, powdered tissues were lysed in 150 µl PBS containing protease inhibitor (pepstatin, Roth, #2936.2; antipain, Roth #2933.2; leupeptin, Roth, #CN33.4), followed by homogenization using an Ultra-Turrax Homogenizer (IKA) and centrifugation for 10 min at 1,000*g*, and the protein concentration was determined using a bicinchoninic acid assay (Thermo Fisher Scientific Reagent A #23228, Reagent B #23224). Cell culture supernatant, tumour lysates and plasma of tumour-bearing mice were analysed using the mouse IL6 Uncoated ELISA Kit (Invitrogen #88706422).

Balb/c mice, bred at the Institute of Molecular Biosciences, Graz, Austria, were maintained in a 14-h light/10-h dark cycle and fed a standard chow diet (ssniff #V1534-000) with ad libitum access to food and water. Then, 12–16-week-old male Balb/c mice were injected with 1 million C26 cells or PBS (100 µl) into the musculus GC of the right hind leg. At study endpoints, blood was drawn via the retro-orbital plexus, mice were euthanised by cervical dislocation, and tissues were excised and snap-frozen in liquid nitrogen. Onset of cachexia was defined by a body weight loss of ≥10% within 2–3 consecutive days. For animal welfare, experiments were discontinued before mice experienced clinically evident functional disorders. If mice underwent rapid weight loss of ≥20 % within a short period, the experiment was terminated and mice were euthanised. Animal handling and experimentation were performed in accordance with the institutional animal welfare officer, and the necessary licences were obtained from by the Austrian Federal Ministry for Science, Research, and Economy (BMBWF-66.007/0005-V/3b/2019).

### Clinical study

The study was approved by the Ethics Committee of the Medical Faculty of the Technical University of Munich (project number 409/16 S) and is registered under *Deutsches Register Klinischer Studien* (DRKS00017285). All patients provided written informed consent before participation in this study, and all procedures were conducted in accordance with the Declaration of Helsinki. Participants were not financially compensated and did not receive any material incentives. Samples of liver (from liver segments III, IVb or V where macroscopically no liver lesions were seen) and musculus rectus abdominis were collected from 28 patients with malignant diseases of the gastrointestinal tract during surgical procedures at the Department of Surgery, *Klinikum rechts der Isar* (TUM University Hospital). Potential biases in patient recruitment may stem from bias in surgical cohort selection (selection of patients considered operable and fit for surgery), consent/self-selection, single-centre design and indication mix (different malignant diseases of the GI tract were included). Sex was considered in the study design as a biological variable because muscle mass, sarcopenia thresholds and metabolic parameters are known to differ between males and females. The sex of participants was recorded as male/female as documented in the medical record (assigned sex at birth based on clinical documentation and surgical records). No gender identity information was collected, and no self-report beyond the medical record was obtained. Given the small cohort (*n* = 28) and the exploratory nature of the study, formal sex-stratified analyses were not performed because of insufficient statistical power. However, we report the sex distribution to enable future meta-analyses.

Tissues were snap-frozen and stored at −80 °C. Upon recruitment before surgery, patients’ clinical characteristics were collected through standardised questionnaires, anthropometry, routine clinical chemistry and medication (MUCABI: Munich Cachexia Biomarkers Working Group). Skeletal muscle area index was evaluated in all patients as described previously^55,56^. Sarcopenia was defined as skeletal muscle area index <52.4 cm^2^ m^−2^ for men and <38 cm^2^ m^−2^ for women^57^. Classification of adenocarcinoma of the oesophagogastric junction (AEG) tumours followed the Siewert system: AEG types I–II were categorised as oesophageal adenocarcinoma, while AEG type III was categorised as gastric adenocarcinoma. Squamous cell carcinoma of the esophagus was grouped under oesophageal carcinoma.

### Real-time quantitative PCR analysis of mouse and human tissues

Pieces of mouse frozen tissues were homogenised in TRIzol (Life Technologies #15596018). RNA was then isolated by adding chloroform. After centrifugation at 12,000*g* for 20 min, the upper phase containing RNA was collected and a volume of 0.6 absolute ethanol was added (for example, 240 µl ethanol for 400 µl of collected upper phase). RNA samples were then loaded onto an Econospin column (Econospin #1920-250), washed three times with RPE buffer (Qiagen #1018013), dried and eluted by adding RNA/DNAse-free water. RNA concentration was determined using Thermo Fisher #Nanodrop 2000. RNA from patients’ tissue samples was isolated using TRIzol reagent and the MirVana miRNA Kit according to the manufacturer’s instructions (Thermo Fisher Scientific). The amount and integrity of isolated RNA was assessed using a bioanalyser (Agilent Technologies). RNA was treated with DNase I (Thermo Fisher Scientific #18068-015) and reverse transcribed into cDNA (Life Technologies #4368814) according to the manufacturers’ instructions.

Real-time quantitative polymerase chain reaction (PCR) was performed using the Applied Biosystems QuantStudio 6 Flex Real-Time PCR System (Applied Biosystems, #4485691). Mouse and Human Taqman gene expression assays are listed in Supplementary Table 6. mRNA levels were determined using TakyonTM Low ROX Probe 2X MasterMix dTTP blue (Eurogentec #UF-LPMT-B0710). Gene expression was normalised to *Tbp* (mouse liver and adipose tissue), *Hprt* (mouse GC muscle) and *GAPDH* (human tissues) mRNA levels. Gene expression 2^−ΔCt^ values were used for statistical analysis.

### Cell culture experiments

All cell lines were tested for mycoplasma contamination by PCR according to the manufacturer’s instructions (Promokine, #PK-CA91–1048).

### C2C12 cell culture

C2C12 cells (ATCC #CRL-1772) were seeded at a density of 5,000 cells cm^−^^2^ and grown in culture medium (high-glucose DMEM with pyruvate and L-glutamine (Thermo Fisher Scientific #41966052) supplemented with 10% heat-inactivated foetal bovine serum (Sigma-Aldrich #F7524) and 1% penicillin–streptomycin (Thermo Fisher Scientific #15140122)) until they reached 80–100% confluence. To induce differentiation, media were switched to 2% foetal bovine serum for 5 days. Long contractile myotubes were used for experiments. Experiments were repeated using different independent cultures of myotubes.

### 3T3-L1 cell culture

3T3-L1 cells (ATCC #CL-173) were seeded at a density of 6,250 cells cm^−^^2^ and grown in culture medium (specified above) for 4 days until they reached 100% confluence. Differentiation was initiated by adding 1 μg ml^−1^ insulin (Sigma-Aldrich #I2643), 0.25 μM dexamethasone (Sigma-Aldrich #D4902), 0.5 mM 3-isobutyl-1-methylxanthine (Sigma-Aldrich #I5879), 50 µg ml^−1^ L-ascorbate (Sigma-Aldrich #A5960), 1 µM biotin (Sigma-Aldrich #B4639) and 17 µM D-pantothenate (Sigma-Aldrich #P5155) to the media. Dexamethasone and 3-isobutyl-1-methylxanthine were removed from the media after 4 days and insulin, ascorbate, biotin and pantothenate after 6 days of differentiation. The cells were cultured for three additional days in culture media before use. Experiments were repeated using different independent cultures of adipocytes.

### Treatment with L-methionine

L-methionine (Sigma-Aldrich #M5308) and L-cysteine (Sigma-Aldrich #C7352) were dissolved in DMEM high-glucose medium with pyruvate and without L-methionine, L-cysteine and L-glutamine (Sigma-Aldrich #D0422) to prepare stock solutions of 0.1 M. Stock solutions were adjusted to pH 7.4 with NaOH, aliquoted and stored at −20 °C.

On the day of the experiment, solutions with different concentrations of L-methionine were prepared. DMEM high-glucose medium with pyruvate and without L-methionine, L-cysteine and L-glutamine was supplemented with the following: 1% penicillin–streptomycin, 4 mM L-glutamine (Thermo Fisher Scientific #25030081), 0.15 mM L-cysteine, 1% (myotubes) or 5% (adipocytes) dialysed serum (poor in amino acids, Thermo Fisher Scientific #A3382001) and 0–100 µM L-methionine. The concentrations of L-glutamine and L-cysteine were matched to usual concentrations in the classical C2C12 and 3T3-L1 culture media. Dialysed serum was kept to a minimum to limit contaminations by serum L-methionine. Media were sterile filtered, and cells were treated for 24 h (lipolysis assay) to 48 h (myotube atrophy, measurement of glucose in media).

### Treatment with FIDAS-5 and recombinant IL6

FIDAS-5 (MCE, HY-136144) was dissolved in dimethyl sulfoxide (AppliChem #A3672) to prepare a stock solution of 5 mM, aliquoted and stored at −20 °C. On the day of the experiment, cells were treated with either vehicle, 2 µM or 5 µM FIDAS-5 for 48 h. All conditions were treated with equal final amounts of dimethyl sulfoxide. Treatments were refreshed every 24 h.

Recombinant IL6 (Thermo Fischer Scientific #216-16) was dissolved in sterile water:bovine serum albumin (BSA) 0.1% (Sigma-Aldrich #A7030) to prepare a stock solution of 100 ng µl^−1^, aliquoted and stored at −20 °C. Cells were treated with 100 ng ml^−1^ IL6 or equal amounts of water:BSA and different doses of FIDAS-5 or vehicle. Treatments lasted for a total of 48 h and were renewed every 24 h.

### Myotube measurement

After 48 h treatment, a minimum of three pictures of myotubes per well were recorded (24-well plates, ×40 objective, #Nikon Eclipse Ts2). Then, 40–60 myotubes per well were quantified using ImageJ software, and the average was considered *n* = 1 replicate. The experiment was repeated using different independent cultures of myotubes.

### Lipolysis assay

After 24 h treatment, glycerol released into the medium was measured using a commercially available kit (Sigma-Aldrich #F6428). Data were normalised to the initial glycerol content in the media and to the total protein content to account for cell number.

For measurement of non-esterified fatty acid release, after 24 h of treatment with L-methionine, cells were starved for 1 h in Krebs Ringer (KR) buffer pH 7.4 (115 mM NaCl, 5.9 mM KCl, 1.2 mM NaH_2_PO_4_, 1.2 mM MgCl_2_, 1.2 mM MgSO_4_, 2.5 mM CaCl_2_ and 25 mM NaHCO_3_) without glucose. Cells were then incubated for 3 h in KR supplemented with 3% BSA, 25 mM HEPES and 5 mM glucose with or without 10 µM isoproterenol to assess stimulated and basal lipolysis, respectively. Non-esterified fatty acid levels in media were measured using a commercially available kit (WAKO #434-91795, #436-91995). Results were normalised to cell protein content.

### Glucose and pH measurement

Conditioned media of C2C12 cells were collected after 48 h treatment. Media pH was estimated using pH test strips (Supelco #1.09543.0001). The rest of the media was frozen at −20 °C until further analysis. Glucose concentration in media was measured using a colorimetric assay (Beckman Coulter glucose reagents #OSR6121). In brief, 1.5 µl of media was loaded into a 96-well plate and mixed with 160 µl of reagent R1 previously diluted 4× in distilled water. After 4-min incubation at 37 °C, absorbance at 340 nm was measured (Abs 1). Then, 40 µl of reagent R2 previously diluted 2× in distilled water was added per well, the plate was incubated at 37 °C for 5 min, and absorbance was measured at 340 nm (Abs 2). Glucose levels were determined by subtracting Abs 1 from Abs 2 (Abs 2 − Abs 1). Absorbance from the blank was then subtracted from all samples. Lastly, glucose concentration for each well was extrapolated from absorbance values of standards with known concentrations (Beckman Coulter glucose standard #66300).

### Western blot analysis

Cells were lysed in ice-cold RIPA (Sigma-Aldrich #R0278) supplemented with 1× cOmplete protease inhibitor cocktail and 1× PhosSTOP cocktail (Sigma-Aldrich (Roche) #11836170001, #4906837001). Protein extracts were separated on 4–20% Tris-glycine gels (Invitrogen #XP04205BOX) and blotted onto nitrocellulose membranes (Cytiva #10600002) using the Trans-Blot Turbo Transfer System (Bio-Rad). After Ponceau staining (Sigma-Aldrich #P7170) and blocking in 5% milk for 1 h, membranes were incubated overnight with the following primary antibodies: p-STAT3(Y705) (Santa Cruz Biotechnology #sc-7993, 1:2,500), total STAT3 (Cell Signaling Technology #12640 clone D3Z2G, 1:5,000), β-actin (Cell Signaling Technology #4967, 1:5,000). Anti-rabbit (Bio-Rad Laboratories #1705046, 1:10,000) coupled to horseradish peroxidase was used as secondary antibodies, and immunoreactive proteins were determined by chemiluminescence using Pierce ECL Western Blotting Substrate (ThermoFisher #32209) or SuperSignal West Femto Maximum Sensitivity Substrate (ThermoFisher #34095) and the ChemiDoc MP System (Bio-Rad). Uncropped original blots from Extended Data Fig. 6k can be found in Source Data Extended Data Fig. 6k.

### Tracer experiments

After 41-h treatment in media supplemented with different doses of L-methionine, cells were treated for 7 h with [^13^C_6_]-glucose tracer. As medium depleted in both glucose and amino acids was not commercially available, we prepared physiological KR buffer pH 7.4 supplemented with the following to match usual C2C12 culture conditions: 4.5 g l^−1^ [^13^C_6_]-glucose, 1 mM pyruvate, 4 mM glutamine, 0.15 mM L-cysteine, 1 mM HEPES, 1% dialysed serum and 0–100 µM L-methionine. After 7-h incubation, cells were washed warm PBS, and plates were immediately frozen at −80 °C until further processing.

After 48-h treatment in media supplemented with different doses of L-methionine, cells were treated with [1-^13^C]-pyruvate tracer (Sigma-Aldrich #490709). KR buffer pH 7.4 was supplemented with 1 mM [1-^13^C]-pyruvate, 1 mM HEPES, 1% dialysed serum and 0 or 100 µM L-methionine. After 0, 5, 10 and 60 min incubation time, cells were washed in warm PBS, and plates were immediately frozen at −80 °C until further processing.

### Data and statistical analysis

Data collection and analysis were not performed blind to the conditions of the experiments. Statistical analysis for the above-mentioned experiments (except -omics) was performed using GraphPad Prism 10. Normality was tested using the Shapiro–Wilk normality test. Equal variances were not formally tested. Statistical tests were two-sided, non-adjusted. Unpaired Student’s *t*-test and Mann–Whitney test were performed to compare two conditions. Unpaired one-way analysis of variance (ANOVA) with Tukey’s or Dunnett’s post-hoc tests, or Kruskal–Wallis with Dunn’s post-hoc tests, were applied to compare more than two groups. Paired or unpaired two-way ANOVA with Tukey’s, Dunnett’s or Šidák’s post-hoc tests were used to compare two variables. Tracing data were analysed using unpaired, non-adjusted, Student’s *t*-test or ANOVA with Dunnett’s post-hoc tests. No statistical methods were used to predetermine sample sizes, but our sample sizes are similar to previous publications^28,58^.

### Metabolomics

#### Tracer metabolomics

Samples were processed via the liquid chromatography (LC)–mass spectrometry (MS) workflow LIMeX (LIpids, Metabolites and eXposome compounds) for simultaneous extraction of complex lipids, polar metabolites and exposome compounds that combines LC–MS untargeted and targeted analysis. Extraction of metabolites was carried out using a biphasic solvent system of cold methanol, methyl *tert*-butyl ether and 10% methanol^19^. For plasma, cells and non-fat tissues, an aliquot of the upper organic phase was collected, evaporated, resuspended in methanol with [12-[(cyclohexylamino) carbonyl]amino]-dodecanoic acid (CUDA) internal standard and analysed using lipidomics platforms. For adipose tissues, one aliquot of the upper organic phase was resuspended in methanol with CUDA internal standard for the analysis of abundant TG species, while the other aliquot was resuspended in 80% methanol with CUDA internal standard to cover minor polar lipid species. For all matrices, an aliquot of the bottom aqueous phase was collected, evaporated, resuspended in an acetonitrile/water (4:1, v/v) mixture with CUDA and Val–Tyr–Val internal standards, and analysed using a HILIC metabolomics platform. Another aliquot of the bottom aqueous phase was treated with an acetonitrile–isopropanol mixture (1:1, v/v), evaporated, resuspended in 5% methanol with 0.2% formic acid with CUDA and Val–Tyr–Val internal standards, and analysed using an HSS T3 metabolomics platform.

#### LC–MS analysis and data processing

Five different LC–MS platforms (LIMeX-5D) in positive and negative ionization mode were used^19^. Experimental samples were randomised within the organ and measured together with quality control and blank samples. Iterative exclusion MS/MS^59^ was performed on quality control samples and reinjection of non-labelled (time 0 min) samples to increase number of annotated metabolites. The LC–MS system consisted of a Vanquish UHPLC System (Thermo Fisher Scientific) coupled to a Q Exactive Plus mass spectrometer (Thermo Fisher Scientific). Polar metabolites were separated on an Acquity UPLC BEH Amide column (50 × 2.1 mm; 1.7 mm) and detected in positive and negative electrospray ionization (ESI) mode, and on an Acquity UPLC HSS T3 column (50 × 2.1 mm; 1.8 mm) (Waters) and detected in negative ESI mode. Non-polar metabolites were separated on an Acquity UPLC BEHC18 column (50 × 2.1 mm; 1.7 mm) and detected in positive and negative ESI mode as before^60^. For ^13^C-metabolomics profiling, the instrument acquired MS1 data only at a mass resolving power of 140,000 full width at half maximum (FWHM; *m*/*z* 200). For metabolite annotation, MS1 data were acquired at a mass resolving power of 35,000 FWHM along with data-dependent MS/MS scans at 17,500 FWHM.

Raw data were processed through the software MS-DIAL 4.94 (ref. ^61^) using an in-house retention time–*m*/*z* library and using MS/MS libraries available from public and commercial sources (MassBank, MoNA, NIST20). *m*/*z* values for all theoretical ^13^C isotopologues were calculated for annotated metabolites in Python and raw data processed through MS-DIAL 4.94. All analytes in MS-DIAL were manually curated. Peak areas were normalised according to sample weight, volume or cell count. They were also adjusted for the natural abundance of elements and the purity of the tracer using IsoCor 2.2 (ref. ^62^). Metabolites were annotated according to the Human Metabolome Database classification systems.

#### Bioinformatics

Metabolite values were log-transformed before further processing. Absolute metabolite values greater than the mean plus four times the standard deviation were replaced with missing values. Metabolites with more than 25% missing values across all conditions were excluded from further analysis, followed by a relative standard deviation filter that removed metabolites with a relative standard deviation above the 80th percentile in any condition. Remaining missing values were imputed where necessary using the knn imputation.

Differentially regulated metabolites between conditions were identified performing one-way ANOVA following post-hoc correction based on Tukey’s honestly significant difference procedure. Statistical analysis of metabolomics data was done using MATLAB R2024A. Heatmaps were created using MATLAB’s bioinfomatics toolbox using clustergram function.

Metabolites were classified on the basis of the database from Metabolon and self-assignment. Energy-related metabolites included glycolysis, TCA metabolites, ketones and derivatives (Supplementary Table 1y).

Intersections of detected and statistically significant metabolites were produced using the R package dplyr v1.1.4 (https://CRAN.R-project.org/package=dplyr) and visualised in the form of Upset plots using the R package UpsetR v4.3.2 (https://CRAN.R-project.org/package=UpSetR) in R version 4.3.1 (https://www.R-project.org/).

For easy data visualization, visit our WebApp (https://m3cav.metabolomics.fgu.cas.cz/).

#### PLSDAs and 3D PCAs

PLSDAs and 3D PCAs were performed using MetaboAnalyst 6.0 based on log-transformed, imputed and scaled data of metabolites (for the sum of unlabelled and all isotopologues, see Supplementary Table 1). Using the ‘Statistical analysis (one factor)’ module from the software, the following parameters were set for the analysis: interquantile range (percentage to filter out 10%, default parameter), mean intensity value, no sample normalization, no data transformation, no data scaling. Ellipses represent 95% confidence intervals.

#### Pathway analysis

Pathway analysis of metabolites commonly regulated in at least two cachexia target tissues was performed using MetaboAnalyst 6.0. Using the ‘Pathway analysis’ module, the Human Metabolome Database lists of metabolites either up- or downregulated in cachexia target tissues were loaded into the system. Pathway analysis was then performed on the metabolite set library pathway based on ‘*Mus musculus* KEGG’.

#### Cluster analysis

Variance-sensitive fuzzy clustering was used to explore cachexia progression in time^63^. Metabolomics datasets per all organs and plasma were sorted into eight groups by the variance-based clustering method, Xie–Beni index (three groups: Ctrl, Pre-cax and Cax; ordered, and two conditions: up or down; eight combinations), describing the trend in time (Non-cax instead of Ctrl used for tumour). Only the subset of analytes assigned with cluster memberships and time profiles was used for further processing. A Sankey plot was used to illustrate the contribution of metabolite profiles among organs to clusters and the class membership of the metabolites. Top ten metabolites or organs per cluster based on membership values were plotted together with the time profiles for illustration.

#### Metabolic flux analysis

^13^C-metabolic flux analysis was implemented using the Isotopomer Network Compartmental Analysis (INCA) 2.3 software^64^ and IsoCor^65^. INCA was used to determine all fluxes through least-squares regression. First, we defined the network reactions involved in the studied pathways, including their corresponding atom transitions. Metabolic fluxes were then estimated by minimising the sum of squared residuals between the simulated and experimentally measured mass isotopomer distribution vectors. [^13^C_6_]-glucose was used as the primary tracer, and all details regarding the model network are included in Supplementary Table 2. A *χ*^2^ test was used to assess goodness of fit and to calculate 95% confidence intervals of estimated fluxes using Monte Carlo simulations. Therefore, when two 95% intervals do not overlap, the distributions are significantly different at *P* < 0.05 (interval plots). Citrate synthase reaction (V12) was set as a reference flux (value 100). Due to non-stationary conditions and metabolic disturbances triggered by the cancer or cachexia, the mass distribution vector standard error was set to 3%.

### Transcriptomics

#### RNA extraction and sequencing

This analysis was performed at Novogene. RNA was extracted as described for qPCR. A total amount of 1 μg RNA per sample was used as input material. Sequencing libraries were generated using NEBNext UltraTM RNA Library Prep Kit for Illumina (NEB), and index codes were added to attribute sequences to each sample (‘Novogene NGS RNA Library Prep Set PT042’). mRNA was purified using poly-T oligo-attached magnetic beads. Fragmentation was carried out using divalent cations under elevated temperature in NEBNext First Strand Synthesis Reaction Buffer (5×). First-strand cDNA was synthesised using random hexamer primer and M-MuLV Reverse Transcriptase (RNase H-). Second-strand cDNA synthesis was subsequently performed using DNA Polymerase I and RNase H. Overhangs were converted into blunt ends via exonuclease and polymerase activities. After adenylation of 3′ ends of DNA fragments, NEBNext Adaptor with hairpin loop structure was ligated to prepare for hybridization. Library fragments were purified with AMPure XP system (Beckman Coulter). Three microlitres USER Enzyme (NEB) with size-selected, adaptor-ligated cDNA was used at 37 °C for 15 min, followed by 5 min at 95 °C. PCR was then performed with Phusion High-Fidelity DNA polymerase, Universal PCR primers and Index (X) Primer. PCR products were purified (AMPure XP system), and library quality was assessed with the Agilent Bioanalyser 2100. Clustering of the index-coded samples was performed on a cBot Cluster Generation System using PE Cluster Kit cBot-HS (Illumina). After cluster generation, the library preparations were sequenced on the Illumina NovaSeq platform, and 150-base paired-end reads were generated. Raw data are available under GEO accession number GSE290937.

#### Bioinformatics

This analysis was performed by Novogene. Raw data (FASTQ files) were processed using in-house Perl scripts. Clean reads were obtained by removing reads containing adapters, reads containing poly-N and low-quality reads. The Q20, Q30 and GC content of the clean data was calculated. Downstream analyses were based on the clean high-quality data. Reference genome (GRCm39) and gene model annotation files were from the genome website directly. The index of the reference genome was built using Hisat2 v2.0.5. The FPKM (expected number of fragments per kilobase of transcript sequence per millions base pairs sequenced) of each gene was calculated based on the length of the gene and reads count mapped to this gene. Differential expression analysis of two groups (four animals per group) was performed using the DESeq2Rpackage (1.20.0). The resulting *P* values were adjusted using the Benjamini–Hochberg approach for controlling the false discovery rate. Genes with an adjusted *P* value (*P*_adj_) <0.05 found by DESeq2 were assigned as differentially expressed. Before differential gene expression analysis, for each sequenced library, the read counts were adjusted by the edgeR program package through one scaling normalised factor. Intersections of significant genes (with an adjusted *P* value <0.05) among the different groups were visualised in Venn diagrams using VennDiagramRpackage v1.7.3.

#### IPA

Comparative pathway analysis of tissue transcriptomics and metabolomics was performed with Ingenuity Pathway Analysis (IPA, Qiagen). For each cachexia target tissue, tables containing gene names and log_2_ fold change (Cax/Ctrl) of gene expression or metabolite levels significantly altered (*P*_adj_ < 0.05 for transcriptomics, *P* < 0.05 for all metabolomics except muscle with *P* <0.1) in cachexia were loaded into the software. Using these tables, we performed a comparative analysis of tissue transcriptomes and transcriptomes × metabolomes. Comparative analysis provided canonical pathways jointly altered in the different cachexia target tissues as well as potential upstream regulators.

### Reporting summary

Further information on research design is available in the Nature Portfolio Reporting Summary linked to this article.

## Supplementary information

Supplementary Information Supplementary Figs. 1–4.

Reporting Summary

Supplementary Data 1 Supplementary Fig. 4. **a**,**b**, C2C12 myotubes were treated with 0 µM or 100 µM of L-methionine for 48 h before being exposed to 1 mM [1-^13^C] pyruvate for 0, 5, 10 and 60 min (*n* = 3 replicates per group). Incorporation of labelled carbons from [1-^13^C] pyruvate into metabolites of the TCA cycle: citrate (**a**) and malate (**b**). Unlabelled metabolites are referred to as M + 0 and isotopically labelled metabolites as M + *X*, where *X* represents the number of labelled carbon atoms. Data are presented as MS signal intensity (arbitrary units, AU). Data are the mean ± s.e.m. Statistical analysis: two-way ANOVA with Tuckey’s post-hoc tests. **P < 0.01, ***P < 0.001, ****P < 0.0001.

Supplementary Table 1 Morigny et al. Supplementary Table 1.

Supplementary Table 2 Morigny et al. Supplementary Table 2.

Supplementary Table 3 Morigny et al. Supplementary Table 3.

Supplementary Table 4 Morigny et al. Supplementary Table 4.

Supplementary Table 5 Morigny et al. Supplementary Table 5.

Supplementary Table 6 Morigny et al. Supplementary Table 6.

## Source data

Source Data Fig. 1 Statistical source data.

Source Data Fig. 3 Statistical source data.

Source Data Fig. 4 Statistical source data.

Source Data Fig. 5 Statistical source data.

Source Data Fig. 6 Statistical source data.

Source Data Fig. 7 Statistical source data.

Source Data Fig. 8 Statistical source data.

Source Data Extended Data Fig. 2 Statistical source data.

Source Data Extended Data Fig. 4 Statistical source data.

Source Data Extended Data Fig. 5 Statistical source data.

Source Data Extended Data Fig. 6 Statistical source data.

Source Data Extended Data Fig. 6k Uncropped western blot.

Source Data Extended Data Fig. 7 Statistical source data.

Source Data Extended Data Fig. 8 Statistical source data.

Source Data Extended Data Fig. 9 Statistical source data.

Source Data Extended Data Fig. 10 Statistical source data.

## Data Availability

RNA sequencing raw data are available under GEO accession number GSE290937. C26 metabolomics data are available via our WebApp at https://m3cav.metabolomics.fgu.cas.cz/. Additional metabolomics and tracing data are available in the Source data. Myotube pictures are available upon request. Source data are provided with this paper.

## References

[1] Fearon, K. et al. Definition and classification of cancer cachexia: an international consensus. *Lancet Oncol.* **12**, 489–495 (2011). 21296615 10.1016/S1470-2045(10)70218-7

[2] Berriel Diaz, M., Rohm, M. & Herzig, S. Cancer cachexia: multilevel metabolic dysfunction. *Nat. Metab.* **6**, 2222–2245 (2024). 39578650 10.1038/s42255-024-01167-9

[3] Kaltenecker, D. et al. Functional liver genomics identifies hepatokines promoting wasting in cancer cachexia. *Cell* **188**, 4549–4566.e22 (2025). 40695279 10.1016/j.cell.2025.06.039

[4] Rupert, J. E. et al. Tumor-derived IL-6 and trans-signaling among tumor, fat, and muscle mediate pancreatic cancer cachexia. *J. Exp. Med.* **218**, e20190450 (2021). 33851955 10.1084/jem.20190450 PMC8185651

[5] O’Connell, T. M. et al. Metabolic biomarkers for the early detection of cancer cachexia. *Front. Cell Dev. Biol.* **9**, 720096 (2021). 34621740 10.3389/fcell.2021.720096 PMC8490779

[6] Cala, M. P. et al. Multiplatform plasma fingerprinting in cancer cachexia: a pilot observational and translational study. *J. Cachexia Sarcopenia Muscle* **9**, 348–357 (2018). 29464940 10.1002/jcsm.12270 PMC5879957

[7] Miller, J. et al. Plasma metabolomics identifies lipid and amino acid markers of weight loss in patients with upper gastrointestinal cancer. *Cancers* **11**, 1594 (2019). 31635032 10.3390/cancers11101594 PMC6826420

[8] Morigny, P. et al. High levels of modified ceramides are a defining feature of murine and human cancer cachexia. *J. Cachexia Sarcopenia Muscle* **11**, 1459–1475 (2020). 33090732 10.1002/jcsm.12626 PMC7749558

[9] DeBerardinis, R. J. & Thompson, C. B. Cellular metabolism and disease: what do metabolic outliers teach us?. *Cell* **148**, 1132–1144 (2012). 22424225 10.1016/j.cell.2012.02.032 PMC3337773

[10] Ballaro, R. et al. Targeting mitochondria by SS-31 ameliorates the whole body energy status in cancer- and chemotherapy-induced cachexia. *Cancers* **13**, 850 (2021). 33670497 10.3390/cancers13040850 PMC7923037

[11] Cui, P. et al. Metabolic profiling of tumors, sera, and skeletal muscles from an orthotopic murine model of gastric cancer associated-cachexia. *J. Proteome Res.* **18**, 1880–1892 (2019). 30888184 10.1021/acs.jproteome.9b00088

[12] Der-Torossian, H. Cancer cachexia’s metabolic signature in a murine model confirms a distinct entity. *Metabolomics* **9**, 730–739 (2013).

[13] Lautaoja, J. H. et al. Muscle and serum metabolomes are dysregulated in colon-26 tumor-bearing mice despite amelioration of cachexia with activin receptor type 2B ligand blockade. *Am. J. Physiol. Endocrinol. Metab.* **316**, E852–E865 (2019). 30860875 10.1152/ajpendo.00526.2018

[14] Pin, F., Barreto, R., Couch, M. E., Bonetto, A. & O’Connell, T. M. Cachexia induced by cancer and chemotherapy yield distinct perturbations to energy metabolism. *J. Cachexia Sarcopenia Muscle* **10**, 140–154 (2019). 30680954 10.1002/jcsm.12360 PMC6438345

[15] Potgens, S. A. et al. Multi-compartment metabolomics and metagenomics reveal major hepatic and intestinal disturbances in cancer cachectic mice. *J. Cachexia Sarcopenia Muscle* **12**, 456–475 (2021). 33599103 10.1002/jcsm.12684 PMC8061360

[16] QuanJun, Y. Integrated analysis of serum and intact muscle metabonomics identify metabolic profiles of cancer cachexia in a dynamic mouse model. *RSC Adv.* **5**, 92438–92448 (2015).

[17] Sun, N. et al. Inter-organ cross-talk in human cancer cachexia revealed by spatial metabolomics. *Metabolism* **161**, 156034 (2024). 39299512 10.1016/j.metabol.2024.156034

[18] Dyar, K. A. et al. Atlas of circadian metabolism reveals system-wide coordination and communication between clocks. *Cell* **174**, 1571–1585.e11 (2018). 30193114 10.1016/j.cell.2018.08.042 PMC6501776

[19] Lopes, M. et al. Metabolomics atlas of oral ^13^C-glucose tolerance test in mice. *Cell Rep.* **37**, 109833 (2021). 34644567 10.1016/j.celrep.2021.109833

[20] Tanaka, Y. et al. Experimental cancer cachexia induced by transplantable Colon 26 adenocarcinoma in mice. *Cancer Res.* **50**, 2290–2295 (1990). 2317817

[21] Reddel, C. J. et al. Increased thrombin generation in a mouse model of cancer cachexia is partially interleukin-6 dependent. *J. Thromb. Haemost.* **15**, 477–486 (2017). 28058802 10.1111/jth.13612

[22] Ducker, G. S. & Rabinowitz, J. D. One-carbon metabolism in health and disease. *Cell Metab.* **25**, 27–42 (2017). 27641100 10.1016/j.cmet.2016.08.009 PMC5353360

[23] Sanderson, S. M., Gao, X., Dai, Z. & Locasale, J. W. Methionine metabolism in health and cancer: a nexus of diet and precision medicine. *Nat. Rev. Cancer* **19**, 625–637 (2019). 31515518 10.1038/s41568-019-0187-8

[24] Mizuno, R. et al. Remote solid cancers rewire hepatic nitrogen metabolism via host nicotinamide-*N*-methyltransferase. *Nat. Commun.* **13**, 3346 (2022). 35705545 10.1038/s41467-022-30926-z PMC9200709

[25] Bindels, L. B. et al. Increased gut permeability in cancer cachexia: mechanisms and clinical relevance. *Oncotarget* **9**, 18224–18238 (2018). 29719601 10.18632/oncotarget.24804 PMC5915068

[26] Chrysostomou, S. E. et al. R-ketorolac ameliorates cancer-associated cachexia and prolongs survival of tumour-bearing mice. *J. Cachexia Sarcopenia Muscle* **15**, 562–574 (2024). 38302863 10.1002/jcsm.13422 PMC10995265

[27] Thibaut, M. M. et al. Inflammation-induced cholestasis in cancer cachexia. *J. Cachexia Sarcopenia Muscle* **12**, 70–90 (2021). 33350058 10.1002/jcsm.12652 PMC7890151

[28] Morigny, P. et al. Association of circulating PLA2G7 levels with cancer cachexia and assessment of darapladib as a therapy. *J. Cachexia Sarcopenia Muscle* **12**, 1333–1351 (2021). 34427055 10.1002/jcsm.12758 PMC8517355

[29] Dumas, J. F. et al. Efficiency of oxidative phosphorylation in liver mitochondria is decreased in a rat model of peritoneal carcinosis. *J. Hepatol.* **54**, 320–327 (2011). 21094554 10.1016/j.jhep.2010.08.012

[30] Visavadiya, N. P., Pena, G. S. & Khamoui, A. V. Mitochondrial dynamics and quality control are altered in a hepatic cell culture model of cancer cachexia. *Mol. Cell. Biochem.* **476**, 23–34 (2021). 32797334 10.1007/s11010-020-03882-9

[31] Rohm, M. et al. An AMP-activated protein kinase-stabilizing peptide ameliorates adipose tissue wasting in cancer cachexia in mice. *Nat. Med.* **22**, 1120–1130 (2016). 27571348 10.1038/nm.4171

[32] Talbert, E. E. et al. Modeling human cancer-induced cachexia. *Cell Rep.* **28**, 1612–1622 (2019). 31390573 10.1016/j.celrep.2019.07.016 PMC6733019

[33] Jiang, Y. J. et al. Establishment of an orthotopic pancreatic cancer mouse model: cells suspended and injected in Matrigel. *World J. Gastroenterol.* **20**, 9476–9485 (2014). 25071342 10.3748/wjg.v20.i28.9476 PMC4110579

[34] More, T. H. et al. Metabolomics analysis reveals novel serum metabolite alterations in cancer cachexia. *Front. Oncol.* **14**, 1286896 (2024). 38450189 10.3389/fonc.2024.1286896 PMC10915872

[35] Mentch, S. J. & Locasale, J. W. One-carbon metabolism and epigenetics: understanding the specificity. *Ann. N. Y. Acad. Sci.* **1363**, 91–98 (2016). 26647078 10.1111/nyas.12956 PMC4801744

[36] Wu, Y. et al. RNA m^1^A methylation regulates glycolysis of cancer cells through modulating ATP5D. *Proc. Natl Acad. Sci. USA* **119**, e2119038119 (2022). 35867754 10.1073/pnas.2119038119 PMC9282374

[37] Newman, A. C. & Maddocks, O. D. K. One-carbon metabolism in cancer. *Br. J. Cancer* **116**, 1499–1504 (2017). 28472819 10.1038/bjc.2017.118 PMC5518849

[38] Murphy, K. T. et al. Mechanisms of chemotherapy-induced muscle wasting in mice with cancer cachexia. *JCSM Rapid Commun.* **5**, 102–116 (2021).

[39] da Silva, R. P., Eudy, B. J. & Deminice, R. One-carbon metabolism in fatty liver disease and fibrosis: one-carbon to rule them all. *J. Nutr.* **150**, 994–1003 (2020). 32119738 10.1093/jn/nxaa032

[40] Fearon, K. C., Glass, D. J. & Guttridge, D. C. Cancer cachexia: mediators, signaling, and metabolic pathways. *Cell Metab.* **16**, 153–166 (2012). 22795476 10.1016/j.cmet.2012.06.011

[41] Stead, L. M., Brosnan, J. T., Brosnan, M. E., Vance, D. E. & Jacobs, R. L. Is it time to reevaluate methyl balance in humans?. *Am. J. Clin. Nutr.* **83**, 5–10 (2006). 16400042 10.1093/ajcn/83.1.5

[42] Liu, K. D. et al. Consequences of lipid remodeling of adipocyte membranes being functionally distinct from lipid storage in obesity. *J. Proteome Res.* **19**, 3919–3935 (2020). 32646215 10.1021/acs.jproteome.9b00894

[43] Lin, K. et al. Disrupted methionine cycle triggers muscle atrophy in cancer cachexia through epigenetic regulation of REDD1. *Cell Metab.* **37**, 460–476.e8 (2024). 39729999 10.1016/j.cmet.2024.10.017

[44] Kojima, Y. et al. Decreased liver B vitamin-related enzymes as a metabolic hallmark of cancer cachexia. *Nat. Commun.* **14**, 6246 (2023). 37803016 10.1038/s41467-023-41952-w PMC10558488

[45] Annibal, A. et al. Regulation of the one carbon folate cycle as a shared metabolic signature of longevity. *Nat. Commun.* **12**, 3486 (2021). 34108489 10.1038/s41467-021-23856-9 PMC8190293

[46] Kosakamoto, H. et al. Early-adult methionine restriction reduces methionine sulfoxide and extends lifespan in *Drosophila*. *Nat. Commun.* **14**, 7832 (2023). 38052797 10.1038/s41467-023-43550-2 PMC10698029

[47] Sengelov, H. et al. Inter-relationships between single carbon units’ metabolism and resting energy expenditure in weight-losing patients with small cell lung cancer. Effects of methionine supply and chemotherapy. *Eur. J. Cancer* **30A**, 1616–1620 (1994). 7833132 10.1016/0959-8049(94)e0148-w

[48] Bao, X. R. et al. Mitochondrial dysfunction remodels one-carbon metabolism in human cells. *eLife* **5**, e10575 (2016). 27307216 10.7554/eLife.10575 PMC4911214

[49] Nikkanen, J. et al. Mitochondrial DNA replication defects disturb cellular dNTP pools and remodel one-carbon metabolism. *Cell Metab.* **23**, 635–648 (2016). 26924217 10.1016/j.cmet.2016.01.019

[50] Rosenberger, F. A. et al. The one-carbon pool controls mitochondrial energy metabolism via complex I and iron–sulfur clusters. *Sci. Adv.* **7**, eabf0717 (2021). 33608280 10.1126/sciadv.abf0717 PMC7895438

[51] Ogunbileje, J. O. et al. Hypermetabolism and hypercatabolism of skeletal muscle accompany mitochondrial stress following severe burn trauma. *Am. J. Physiol. Endocrinol. Metab.* **311**, E436–E448 (2016). 27382037 10.1152/ajpendo.00535.2015 PMC5005969

[52] Porter, C., Herndon, D. N., Sidossis, L. S. & Borsheim, E. The impact of severe burns on skeletal muscle mitochondrial function. *Burns* **39**, 1039–1047 (2013). 23664225 10.1016/j.burns.2013.03.018 PMC3729601

[53] Moser, A. R., Pitot, H. C. & Dove, W. F. A dominant mutation that predisposes to multiple intestinal neoplasia in the mouse. *Science* **247**, 322–324 (1990). 2296722 10.1126/science.2296722

[54] Puppa, M. J. et al. Gut barrier dysfunction in the Apc(Min/+) mouse model of colon cancer cachexia. *Biochim. Biophys. Acta* **1812**, 1601–1606 (2011). 21914473 10.1016/j.bbadis.2011.08.010 PMC3205242

[55] Chovsepian, A. et al. Diabetes increases mortality in patients with pancreatic and colorectal cancer by promoting cachexia and its associated inflammatory status. *Mol. Metab.* **73**, 101729 (2023). 37094629 10.1016/j.molmet.2023.101729 PMC10192649

[56] Prokopchuk, O. et al. A novel tissue inhibitor of metalloproteinases-1/liver/cachexia score predicts prognosis of gastrointestinal cancer patients. *J. Cachexia Sarcopenia Muscle* **12**, 378–392 (2021). 33590974 10.1002/jcsm.12680 PMC8061407

[57] Prado, C. M. et al. Prevalence and clinical implications of sarcopenic obesity in patients with solid tumours of the respiratory and gastrointestinal tracts: a population-based study. *Lancet Oncol.* **9**, 629–635 (2008). 18539529 10.1016/S1470-2045(08)70153-0

[58] Ji, H. et al. Development of a peptide drug restoring AMPK and adipose tissue functionality in cancer cachexia. *Mol. Ther.* **31**, 2408–2421 (2023). 37408309 10.1016/j.ymthe.2023.06.020 PMC10422018

[59] Koelmel, J. P. et al. Expanding lipidome coverage using LC–MS/MS data-dependent acquisition with automated exclusion list generation. *J. Am. Soc. Mass Spectrom.* **28**, 908–917 (2017). 28265968 10.1007/s13361-017-1608-0 PMC5408749

[60] Paluchova, V. et al. Lipokine 5-PAHSA is regulated by adipose triglyceride lipase and primes adipocytes for de novo lipogenesis in mice. *Diabetes* **69**, 300–312 (2020). 31806624 10.2337/db19-0494 PMC7118252

[61] Tsugawa, H. et al. MS-DIAL: data-independent MS/MS deconvolution for comprehensive metabolome analysis. *Nat. Methods* **12**, 523–526 (2015). 25938372 10.1038/nmeth.3393 PMC4449330

[62] Millard, P., Letisse, F., Sokol, S. & Portais, J. C. IsoCor: correcting MS data in isotope labeling experiments. *Bioinformatics* **28**, 1294–1296 (2012). 22419781 10.1093/bioinformatics/bts127

[63] Schwammle, V. & Jensen, O. N. VSClust: feature-based variance-sensitive clustering of omics data. *Bioinformatics* **34**, 2965–2972 (2018). 29635359 10.1093/bioinformatics/bty224

[64] Rahim, M. et al. INCA 2.0: a tool for integrated, dynamic modeling of NMR- and MS-based isotopomer measurements and rigorous metabolic flux analysis. *Metab. Eng.* **69**, 275–285 (2022). 34965470 10.1016/j.ymben.2021.12.009 PMC8789327

[65] Millard, P. et al. IsoCor: isotope correction for high-resolution MS labeling experiments. *Bioinformatics* **35**, 4484–4487 (2019). 30903185 10.1093/bioinformatics/btz209

