# Supplementary material for: Multi-omics profiling of cachexia-targeted tissues reveals a spatio-temporally coordinated response to cancer
Source: Nat Metab. 2026 Jan 15;8(1):237–59. doi: 10.1038/s42255-025-01434-3 (PMC12855018; doi:10.1038/s42255-025-01434-3)
Supplement: Supplementary file 1 — Supplementary Figs. 1–4. [file 42255_2025_1434_MOESM1_ESM.pdf]

nature metabolism

Supplementary information

https://doi.org/10.1038/s42255-025-01434-3

Multi-omics profiling of cachexia-targeted tissues reveals a spatio-temporally coordinated response to cancer

In the format provided by the authors and unedited

a

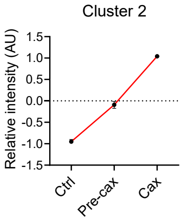

tumour	Aminoadipic acid
tumour	N-Formyl-methionine
iWAT	CDP-Ethanolamine
GC	5'-Methylthioadenosine
liver	Pyridoxamine
iWAT	Citicoline
soleus	Thymine
liver	Thiamine
GC	Methionine sulfoxide
soleus	Lysine

b

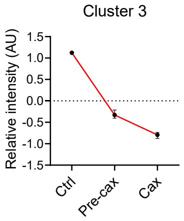

plasma	Carnitine
iWAT	Glyceric acid 1,3-biphosphate
iWAT	Histidinyl-alanine
heart	4-Guanidinobutanoic acid
tumour	Carnitine
plasma	Fumaric acid
tumour	Histidine
heart	Phosphoadenosine phosphosulfate
plasma	Malonic acid
tumour	3-Hydroxyisovalerylcarnitine

c

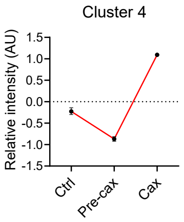

eWAT	Xylulose 5-phosphate
liver	Phenylalanine
plasma	Salicylic acid
plasma	Cysteine-S-sulfate
GC	Citric acid
heart	Niacinamide
iWAT	Glyceric acid
GC	N-Acetylneuraminic acid
liver	Niacinamide
heart	Anserine

d

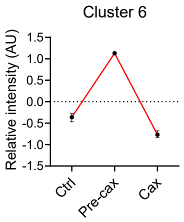

iWAT	gamma-Glutamylcysteine
heart	N-Acetylneuraminic acid
tumour	Citric acid
iWAT	Valyl-arginine
soleus	Glycerol 3-phosphate
tumour	Serylmethionine
tumour	Arginyl-alanine
GC	Malic acid
tumour	trans-Ferulic acid
plasma	2-Amino-3-hydroxypropanoic acid

e

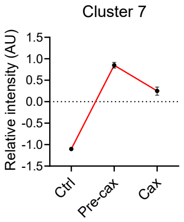

heart	Glutamine
heart	Xylulose 5-phosphate
soleus	Glycyl-valine
tumour	Asparaginyl-serine
GC	N-Acetyl-Glucosamine 6-Phosphate
heart	Arabinose 5-phosphate
plasma	Isocitric acid
tumour	Arabinose 5-phosphate
tumour	Cytidine monophosphate
GC	Isoleucyl-aspartate

f

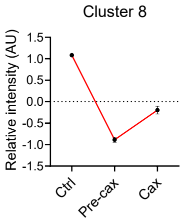

GC	Taurine
GC	Suberic acid
soleus	Capric acid
GC	Quinolinic acid
GC	Spermidine
iWAT	Folic acid
plasma	Glutaric acid
tumour	Ergothioneine
tumour	cyclic ADP-ribose
heart	S-Adenosylhomocysteine

Supplemental Data Fig. 1: See also Fig. 1a for experimental setup. Ctrl: healthy controls, no tumour; Non-cax: non-cachectic tumour mice; Pre-cax: pre-cachectic tumour mice; Cax: cachectic tumour mice. n = 4 animals per group. **(a-f)** Cluster analysis of metabolite trajectories in the time course of cachexia development: Ctrl (cachexia target tissues) or Non-Cax (tumours) à Pre-cax à Cax. Top 10 metabolites of clusters 2 **(a)**, 3 **(b)**, 4 **(c)**, 6 **(d)**, 7 **(e)**, 8 **(f)**. See also **Fig. 2a-c** for cluster overview and detailed clusters 2 and 5. Values represent the medians of all metabolites within the clusters, error bars represent the range. The clustering algorithm scaled relative intensities (A.U.).

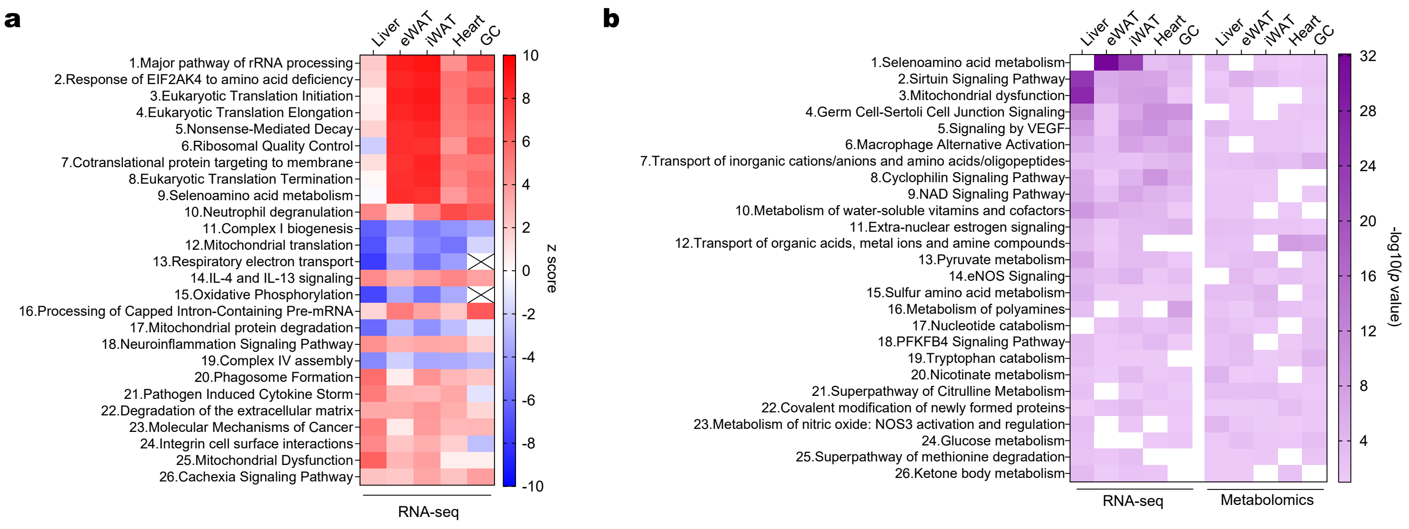

Supplemental Data Fig. 2: Transcriptomic analysis of cachexia target tissues from healthy controls (Ctrl), non-cachectic (Non-cax) and cachectic (Cax) tumour mice. See also Fig. 1a for experimental setup and Fig. 4. n = 4 animals per group. **(a)** Top pathways altered in a similar manner in cachexia target tissues (liver, eWAT, iWAT, heart, GC muscle) from Cax vs. Ctrl mice. Data are represented as top z-scores: pathways predicted to be activated in red and inhibited in blue (Ingenuity Pathway Analysis, IPA, Qiagen). **(b)** Top pathways commonly altered in both transcriptomics and metabolomics datasets based on p value (IPA, Qiagen) in Cax vs. Ctrl mice.

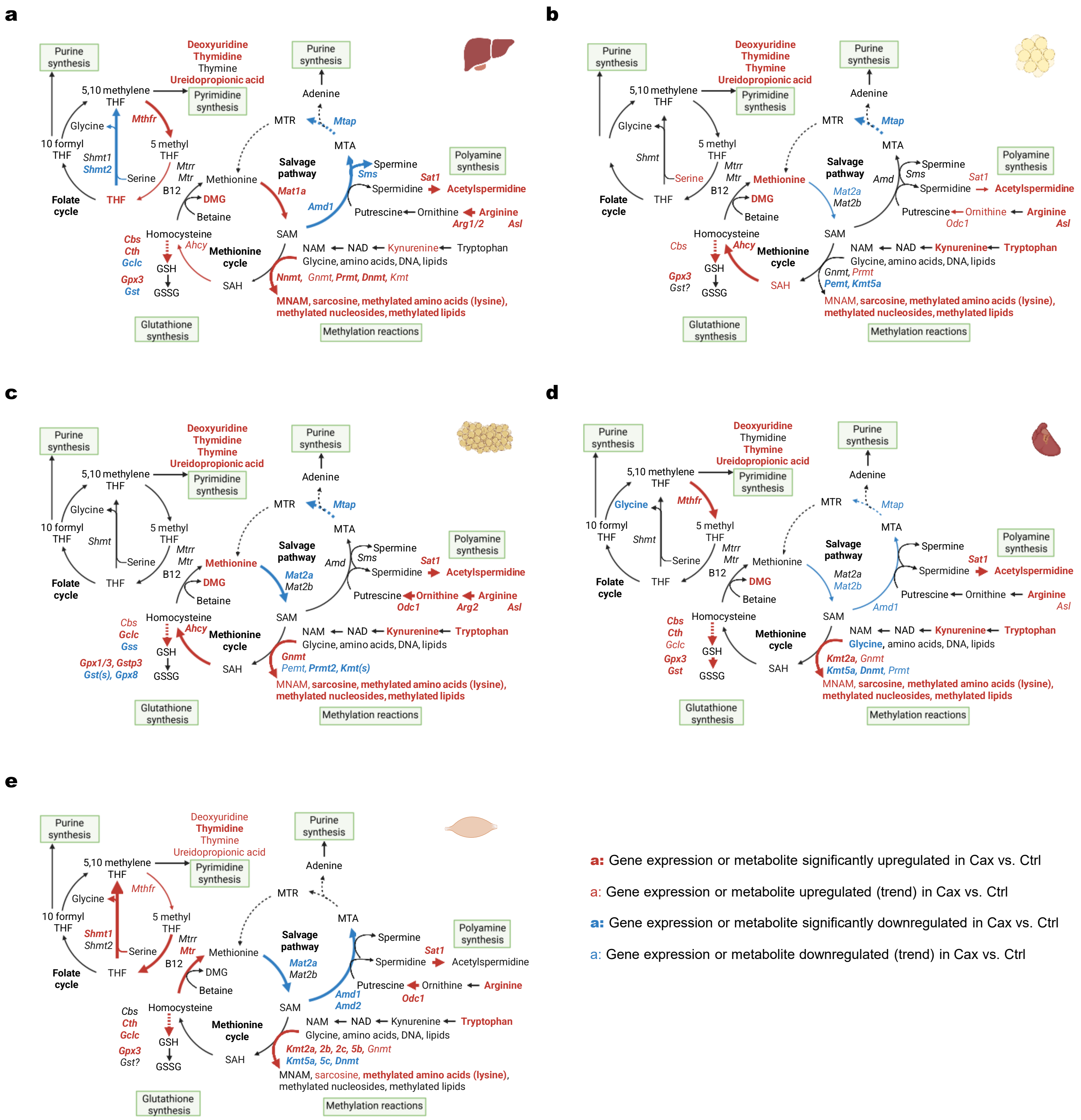

Supplemental Data Fig. 3: (a-e) Visual integration of metabolomics and transcriptomics datasets (data presented in **Fig. 1-4**) showing alterations in one-carbon metabolism in Cax *vs.*Ctrl mice (see **Fig. 1a** for experimental setup). Red, bold font: enzyme or metabolite significantly upregulated in Cax mice. Red, thin font: enzyme or metabolites which trends to be upregulated. Blue, bold font: enzyme or metabolite significantly downregulated. Blue, thin font: enzyme or metabolites which trends to be downregulated. **(a)** Liver, **(b)** eWAT, **(c)** iWAT, **(d)** heart and **(e)** GC muscle. Created in BioRender. Rohm, M. (2025) https://BioRender.com/havo8kv

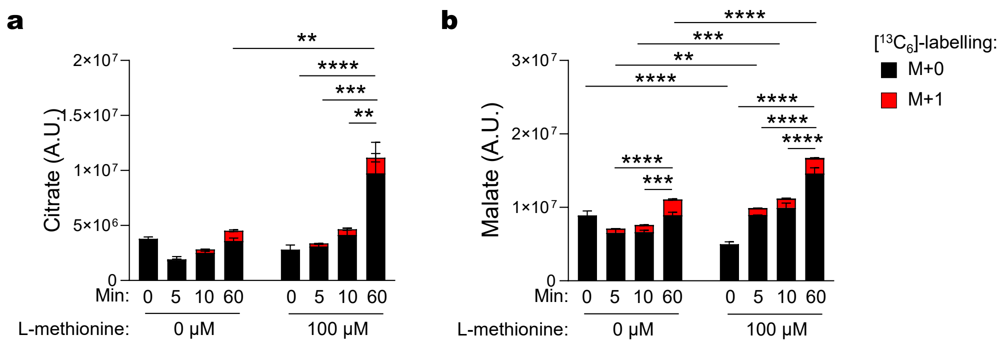

Supplemental Data Fig. 4: (a-b) C2C12 myotubes were treated with 0 µM or 100 µM of L-methionine for 48h before being exposed to 1mM [1-^{13}C] pyruvate for 0, 5, 10 and 60 min (n = 3 replicates per group). Incorporation of labelled carbons from [1-^{13}C] pyruvate into metabolites of the TCA cycle: **(a)** citrate and **(b)** malate. Unlabelled metabolites are referred as M+0, isotopically labelled metabolites as M+X, where X represents the number of labelled carbon atoms. Data are presented as MS signal intensity (arbitrary units A.U.). Data are mean ± s.e.m. Statistical analysis: two-way ANOVA with Tuckey's post-hoc tests. **$p < 0.01$, ***$p < 0.001$, ****$p < 0.0001$.
