# Supplementary material for: Multi-omics profiling of cachexia-targeted tissues reveals a spatio-temporally coordinated response to cancer
Source: Nat Metab. 2026 Jan 15;8(1):237–59. doi: 10.1038/s42255-025-01434-3 (PMC12855018; doi:10.1038/s42255-025-01434-3)
Supplement: Supplementary file 2 — Reporting Summary [file 42255_2025_1434_MOESM2_ESM.pdf]

nature portfolio

Corresponding author(s): Maria Rohm, Ondrej Kuda

Last updated by author(s): Nov 13, 2025

Reporting Summary

Nature Portfolio wishes to improve the reproducibility of the work that we publish. This form provides structure for consistency and transparency in reporting. For further information on Nature Portfolio policies, see our Editorial Policies and the Editorial Policy Checklist.

Statistics

For all statistical analyses, confirm that the following items are present in the figure legend, table legend, main text, or Methods section.

n/a	Confirmed	
☐	☒	The exact sample size (*n*) for each experimental group/condition, given as a discrete number and unit of measurement
☐	☒	A statement on whether measurements were taken from distinct samples or whether the same sample was measured repeatedly
☐	☒	The statistical test(s) used AND whether they are one- or two-sided<br>*Only common tests should be described solely by name; describe more complex techniques in the Methods section.*
☒	☐	A description of all covariates tested
☐	☒	A description of any assumptions or corrections, such as tests of normality and adjustment for multiple comparisons
☐	☒	A full description of the statistical parameters including central tendency (e.g. means) or other basic estimates (e.g. regression coefficient) AND variation (e.g. standard deviation) or associated estimates of uncertainty (e.g. confidence intervals)
☐	☒	For null hypothesis testing, the test statistic (e.g. *F*, *t*, *r*) with confidence intervals, effect sizes, degrees of freedom and *P* value noted<br>*Give P values as exact values whenever suitable.*
☒	☐	For Bayesian analysis, information on the choice of priors and Markov chain Monte Carlo settings
☒	☐	For hierarchical and complex designs, identification of the appropriate level for tests and full reporting of outcomes
☒	☐	Estimates of effect sizes (e.g. Cohen's *d*, Pearson's *r*), indicating how they were calculated

Our web collection on statistics for biologists contains articles on many of the points above.

Software and code

Policy information about availability of computer code

Data collection	No software was used.
Data analysis	Metabolomics data were analysed using MS-DIAL (4.94) software. 13C-metabolic flux analysis was implemented using the INCA 2.2/MATLAB R2024a software and IsoCor (2.2). Partial least-squares discriminant analysis (PLS-DA), principal component analysis (PCA) and pathway analysis were performed using MetaboAnalyst 6.0. Differential gene expression analysis of two groups (four animals per group) was performed using the DESeq2Rpackage (1.20.0). Statistical analysis of data (excluding omics) was performed using GraphPad Prism 10.

For manuscripts utilizing custom algorithms or software that are central to the research but not yet described in published literature, software must be made available to editors and reviewers. We strongly encourage code deposition in a community repository (e.g. GitHub). See the Nature Portfolio guidelines for submitting code & software for further information.

Data

Policy information about availability of data

All manuscripts must include a data availability statement. This statement should provide the following information, where applicable:

- Accession codes, unique identifiers, or web links for publicly available datasets
- A description of any restrictions on data availability
- For clinical datasets or third party data, please ensure that the statement adheres to our policy

All the murine data in this manuscript are original and unpublished. RNAsequencing raw data is available under GEO accession number GSE290937. C26

metabolomics data is available on our WebApp: https://m3cav.metabolomics.fgu.cas.cz/. Additional metabolomics and tracing data is available in the source data files.

Research involving human participants, their data, or biological material

Policy information about studies with human participants or human data. See also policy information about sex, gender (identity/presentation), and sexual orientation and race, ethnicity and racism.

Reporting on sex and gender	Study included patients from both sexes. All patients provided written informed consent before participation in this study and the whole handling complies with the Declaration of Helsinki. Patients' data (number of participants, sex distribution, age, cancer type, cancer stage, skeletal muscle area and index) are summarised in Supplemental Data Table 3.
Reporting on race, ethnicity, or other socially relevant groupings	No categorisation based on race, ethnicity or other socially relevant groups.
Population characteristics	Patients' data (number of participants, sex distribution, age, cancer type, cancer stage, skeletal muscle area and index) are summarised in Supplemental Data Table 3.
Recruitment	Samples of liver (from liver segments III, IVb or V where macroscopically no liver lesions were seen) and musculus rectus abdominis were collected from a total of 28 patients with malignant diseases of the gastrointestinal tract during surgical procedures at the Department of Surgery, Klinikum rechts der Isar (University Hospital of the TUM).
Ethics oversight	The study was approved by the Ethics Committee of the Medical Faculty of the Technical University of Munich (project number 409/16 S) and is registered under “Deutsches Register Klinischer Studien” (DRKS00017285).

Note that full information on the approval of the study protocol must also be provided in the manuscript.

Field-specific reporting

Please select the one below that is the best fit for your research. If you are not sure, read the appropriate sections before making your selection.

☒ Life sciences ☐ Behavioural & social sciences ☐ Ecological, evolutionary & environmental sciences

For a reference copy of the document with all sections, see nature.com/documents/nr-reporting-summary-flat.pdf

Life sciences study design

All studies must disclose on these points even when the disclosure is negative.

Sample size	The number of replicates (n=4) for the mouse tracer study was set based on previous experience to ensure practical feasibility. This decision took into account the complexity of the experiment, which involved collecting multiple organs from each animal in every group, with each organ divided into several aliquots. These samples were then subjected to metabolomic profiling across 6-7 different analytical platforms. The multiplication of these steps—from sample collection to aliquot preparation and multi-platform analysis—significantly increased the complexity of the entire experiment, pushing it to the limits of what was realistically achievable in the laboratory setting. The chosen sample size of n=4 thus represents a balance between statistical relevance and operational manageability, given the extensive analytical workload involved.
Data exclusions	No data excluded.
Replication	Key results from mouse experiments were confirmed in different independent in vivo experiments performed in different institutes across Europe, and using different mouse models of cachexia. In vitro experiments were replicated using independent cell cultures and cell passages (except for in vitro tracer experiment). All attempts at replication were successful.
Randomization	No randomisation. In each animal experiment, mice were assigned to groups in a manner that initial body weight, lean and fat mass were similar between the groups as confirmed by non-significant statistical analysis.
Blinding	No blinding was done because knowledge of experimental conditions was required during animal handling and data collection.

Behavioural & social sciences study design

All studies must disclose on these points even when the disclosure is negative.

Study description	*Briefly describe the study type including whether data are quantitative, qualitative, or mixed-methods (e.g. qualitative cross-sectional, quantitative experimental, mixed-methods case study).*
Research sample	*State the research sample (e.g. Harvard university undergraduates, villagers in rural India) and provide relevant demographic information (e.g. age, sex) and indicate whether the sample is representative. Provide a rationale for the study sample chosen. For studies involving existing datasets, please describe the dataset and source.*

Sampling strategy	*Describe the sampling procedure (e.g. random, snowball, stratified, convenience). Describe the statistical methods that were used to predetermine sample size OR if no sample-size calculation was performed, describe how sample sizes were chosen and provide a rationale for why these sample sizes are sufficient. For qualitative data, please indicate whether data saturation was considered, and what criteria were used to decide that no further sampling was needed.*
Data collection	*Provide details about the data collection procedure, including the instruments or devices used to record the data (e.g. pen and paper, computer, eye tracker, video or audio equipment) whether anyone was present besides the participant(s) and the researcher, and whether the researcher was blind to experimental condition and/or the study hypothesis during data collection.*
Timing	*Indicate the start and stop dates of data collection. If there is a gap between collection periods, state the dates for each sample cohort.*
Data exclusions	*If no data were excluded from the analyses, state so OR if data were excluded, provide the exact number of exclusions and the rationale behind them, indicating whether exclusion criteria were pre-established.*
Non-participation	*State how many participants dropped out/declined participation and the reason(s) given OR provide response rate OR state that no participants dropped out/declined participation.*
Randomization	*If participants were not allocated into experimental groups, state so OR describe how participants were allocated to groups, and if allocation was not random, describe how covariates were controlled.*

Ecological, evolutionary & environmental sciences study design

All studies must disclose on these points even when the disclosure is negative.

Study description	*Briefly describe the study. For quantitative data include treatment factors and interactions, design structure (e.g. factorial, nested, hierarchical), nature and number of experimental units and replicates.*
Research sample	*Describe the research sample (e.g. a group of tagged Passer domesticus, all Stenocereus thurberi within Organ Pipe Cactus National Monument), and provide a rationale for the sample choice. When relevant, describe the organism taxa, source, sex, age range and any manipulations. State what population the sample is meant to represent when applicable. For studies involving existing datasets, describe the data and its source.*
Sampling strategy	*Note the sampling procedure. Describe the statistical methods that were used to predetermine sample size OR if no sample-size calculation was performed, describe how sample sizes were chosen and provide a rationale for why these sample sizes are sufficient.*
Data collection	*Describe the data collection procedure, including who recorded the data and how.*
Timing and spatial scale	*Indicate the start and stop dates of data collection, noting the frequency and periodicity of sampling and providing a rationale for these choices. If there is a gap between collection periods, state the dates for each sample cohort. Specify the spatial scale from which the data are taken*
Data exclusions	*If no data were excluded from the analyses, state so OR if data were excluded, describe the exclusions and the rationale behind them, indicating whether exclusion criteria were pre-established.*
Reproducibility	*Describe the measures taken to verify the reproducibility of experimental findings. For each experiment, note whether any attempts to repeat the experiment failed OR state that all attempts to repeat the experiment were successful.*
Randomization	*Describe how samples/organisms/participants were allocated into groups. If allocation was not random, describe how covariates were controlled. If this is not relevant to your study, explain why.*
Blinding	*Describe the extent of blinding used during data acquisition and analysis. If blinding was not possible, describe why OR explain why blinding was not relevant to your study.*

Did the study involve field work? ☐ Yes ☐ No

Field work, collection and transport

Field conditions	*Describe the study conditions for field work, providing relevant parameters (e.g. temperature, rainfall).*
Location	*State the location of the sampling or experiment, providing relevant parameters (e.g. latitude and longitude, elevation, water depth).*
Access & import/export	*Describe the efforts you have made to access habitats and to collect and import/export your samples in a responsible manner and in compliance with local, national and international laws, noting any permits that were obtained (give the name of the issuing authority, the date of issue, and any identifying information).*
Disturbance	*Describe any disturbance caused by the study and how it was minimized.*

Reporting for specific materials, systems and methods

We require information from authors about some types of materials, experimental systems and methods used in many studies. Here, indicate whether each material, system or method listed is relevant to your study. If you are not sure if a list item applies to your research, read the appropriate section before selecting a response.

Materials & experimental systems

n/a	Involved in the study
☐	☒ Antibodies
☐	☒ Eukaryotic cell lines
☒	☐ Palaeontology and archaeology
☐	☒ Animals and other organisms
☒	☐ Clinical data
☒	☐ Dual use research of concern
☒	☐ Plants

Methods

n/a	Involved in the study
☒	☐ ChIP-seq
☒	☐ Flow cytometry
☒	☐ MRI-based neuroimaging

Antibodies

Antibodies used

Interleukin 6-neutralising antibody: rat anti-murine IL-6 antibody, clone MP5-20F3, BioXCell, NH, USA.
IgG control antibody for in vivo studies: catalogue # BE0088, BioXCell, NH, USA.

p-STAT3(Y705) (Santa Cruz Biotechnology #sc-7993, 1:2500), total STAT3 (Cell Signalling Technology #12640 clone D3Z2G, 1:5000), β-actin (Cell Signalling Technology #4967, 1:5000) for in vitro studies.
Secondary anti-rabbit (Biorad Laboratories #1705046, 1:10,000) coupled to horseradish peroxidase for in vitro studies.

Validation

Neutralising antibody validation was established by the measurement of plasma IL6 levels in tumour-bearing mice. These levels were reduced in mice treated with the anti-IL6 antibody (data published in Bindels et al, Oncotarget 9, 18224-18238 (2018)). Other antibodies were not validated.

Eukaryotic cell lines

Policy information about cell lines and Sex and Gender in Research

Cell line source(s)

C26 colon carcinoma: mouse, cachexia-inducing cell line (Tanaka et al. Cancer Res 50, 2290-2295 (1990), Morigny et al. J Cachexia Sarcopenia Muscle 11, 1459-1475 (2020)). Cells were obtained from the German Cancer Research Center (Deutsches Krebsforschungszentrum, DKFZ Tumorbank).

NC26 colon carcinoma: mouse, non-cachexia-inducing cell line (Morigny et al. J Cachexia Sarcopenia Muscle 11, 1459-1475 (2020)). Cells were kindly provided by Rudolf Zechner and Martina Schweiger from the Institute of Molecular Biosciences (University of Graz, Austria) and were originally obtained from the Cell Resource Center for Biomedical Research-Cell Bank of the Tohoku University (TKG-0518).

Lewis lung carcinoma: mouse, cachexia-inducing cell line (Morigny et al. J Cachexia Sarcopenia Muscle 11, 1459-1475 (2020)). Cells were obtained from ATCC (#CRL-1642).

Panc02 pancreatic ductal adenocarcinoma: mouse, cachexia-inducing cell line (Jiang et al. World J Gastroenterol 20, 9476-9485 (2014); and this study). Cells were kindly provided by Jan Zenka from the Department of Medical Biology, Faculty of Science, University of South Bohemia (Czech Republic).

8025 pancreatic ductal adenocarcinoma: mouse, cachexia-inducing cell line (Kaltenecker et al. Cell. 2025 Aug 21;188(17):4549-4566.e22). Derived from KPC (KrasLSL-G12D; Trp53LSL-R172H; Pdx1-Cre) mice. Cells were kindly provided by Dieter Saur, TU Munich, Germany.

SW480 colon carcinoma: human, cell line (ATCC #CCL-228), cells isolated from the large intestine of a Dukes C colorectal cancer patient. Cells were obtained from the German Cancer Research Center (Deutsches Krebsforschungszentrum, DKFZ Tumorbank).

C2C12 and 3T3-L1 were obtained from ATCC (#CRL-1772 and #CL-173).

Authentication

The cell lines have not been authenticated.

Mycoplasma contamination

Cell lines have undergone mycoplasma checks and tested negative.

Commonly misidentified lines
(See ICLAC register)

No commonly misidentified cell lines were used.

Palaeontology and Archaeology

Specimen provenance	*Provide provenance information for specimens and describe permits that were obtained for the work (including the name of the issuing authority, the date of issue, and any identifying information). Permits should encompass collection and, where applicable, export.*
Specimen deposition	*Indicate where the specimens have been deposited to permit free access by other researchers.*
Dating methods	*If new dates are provided, describe how they were obtained (e.g. collection, storage, sample pretreatment and measurement), where they were obtained (i.e. lab name), the calibration program and the protocol for quality assurance OR state that no new dates are provided.*

☐ Tick this box to confirm that the raw and calibrated dates are available in the paper or in Supplementary Information.

Ethics oversight	*Identify the organization(s) that approved or provided guidance on the study protocol, OR state that no ethical approval or guidance was required and explain why not.*

Note that full information on the approval of the study protocol must also be provided in the manuscript.

Animals and other research organisms

Policy information about studies involving animals; ARRIVE guidelines recommended for reporting animal research, and Sex and Gender in Research

Laboratory animals	In vivo experiments were carried out in 9.5- to 12-week-old male BALB/c (NC26 and C26 models), C57Bl/6J (Panc02, 8025, LLC models) or FoxChase SCID (immune compromised, SW480 model) mice obtained from Charles River Laboratories (Germany and France). APCMin/J mice, as originally described by Moser et al (Moser, R.R. et al, Science 247, 322-324 (1990)) and later established as model of cachexia (Puppa, M.J. et al, Biochim Biophys Acta 1812, 1601-1606 (2011)), were purchased from the Jackson Laboratory (JAX stock #002020) and bred on a C57BL/6J background. KPP founder mice KrasLSL-G12D/+, Pten flox/flox and Ptf1a Cre-ER/+, Pten flox/flox were a gift from Denis C. Guttridge, Ohio State University, Medical University of South Carolina (Talbert, E.E. et al, Cell Rep 28, 1612-1622 e1614 (2019)).Animals were kept at 12h light/dark cycles with exception of the Il6KO cohort in Graz,which was on a 14/10h light/dark cycle.
Wild animals	Study did not involve wild animals.
Reporting on sex	We used only used male mice in our study to maintain consistency in experimental conditions and minimise variability. This decision is grounded in the recognition of reported distinctions in cancer-associated weight loss between male and female cancer patients, with the former exhibiting a more pronounced susceptibility. In mouse models, sex-specific regulations may influence the response to cachexia-inducing cytokines such as IL-6 during the progression of cachexia. Consequently, employing only male mice enhances the precision of our investigation by aligning with the potential sex-specific nuances in disease phenotypes, thereby contributing to a more focused and insightful analysis.<br><br>Barone, M. et al, J Adv Res (2025)<br>Cabrera, A.R. et al, J Appl Physiol (1985) 135, 655-672 (2023)<br>Hetzler, K.L et al, Biochim Biophys Acta 1852, 816-825 (2015)<br>Zhong, X. & Zimmers, T.A. Curr Osteoporos Rep 18, 646-654 (2020)
Field-collected samples	The study did not involve samples collected from the field.
Ethics oversight	Animal handling and experimentation were approved and performed in accordance with the guidelines from the European Union directives and the state ethics committees and government of Upper Bavaria (Germany), Animal Care and Use Committee of the Institute of Physiology of the Czech Academy of Sciences (Czech Republic), UCLouvain (Belgium), and Austrian Federal Ministry for Science, Research, and Economy (Austria).

Note that full information on the approval of the study protocol must also be provided in the manuscript.

Clinical data

Policy information about clinical studies
All manuscripts should comply with the ICMJE guidelines for publication of clinical research and a completed CONSORT checklist must be included with all submissions.

Clinical trial registration	*Provide the trial registration number from ClinicalTrials.gov or an equivalent agency.*
Study protocol	*Note where the full trial protocol can be accessed OR if not available, explain why.*
Data collection	*Describe the settings and locales of data collection, noting the time periods of recruitment and data collection.*
Outcomes	*Describe how you pre-defined primary and secondary outcome measures and how you assessed these measures.*

Dual use research of concern

Policy information about dual use research of concern

Hazards

Could the accidental, deliberate or reckless misuse of agents or technologies generated in the work, or the application of information presented in the manuscript, pose a threat to:

No	Yes	
☐	☐	Public health
☐	☐	National security
☐	☐	Crops and/or livestock
☐	☐	Ecosystems
☐	☐	Any other significant area

Experiments of concern

Does the work involve any of these experiments of concern:

No	Yes	
☐	☐	Demonstrate how to render a vaccine ineffective
☐	☐	Confer resistance to therapeutically useful antibiotics or antiviral agents
☐	☐	Enhance the virulence of a pathogen or render a nonpathogen virulent
☐	☐	Increase transmissibility of a pathogen
☐	☐	Alter the host range of a pathogen
☐	☐	Enable evasion of diagnostic/detection modalities
☐	☐	Enable the weaponization of a biological agent or toxin
☐	☐	Any other potentially harmful combination of experiments and agents

Plants

Seed stocks	N/A
Novel plant genotypes	N/A
Authentication	N/A

ChIP-seq

Data deposition

☐ Confirm that both raw and final processed data have been deposited in a public database such as GEO.

☐ Confirm that you have deposited or provided access to graph files (e.g. BED files) for the called peaks.

Data access links<br>*May remain private before publication.*	*For "Initial submission" or "Revised version" documents, provide reviewer access links. For your "Final submission" document, provide a link to the deposited data.*
Files in database submission	*Provide a list of all files available in the database submission.*
Genome browser session<br>(e.g. UCSC)	*Provide a link to an anonymized genome browser session for "Initial submission" and "Revised version" documents only, to enable peer review. Write "no longer applicable" for "Final submission" documents.*

Methodology

Replicates

Describe the experimental replicates, specifying number, type and replicate agreement.

Sequencing depth

Describe the sequencing depth for each experiment, providing the total number of reads, uniquely mapped reads, length of reads and whether they were paired- or single-end.

Antibodies

Describe the antibodies used for the ChIP-seq experiments; as applicable, provide supplier name, catalog number, clone name, and lot number.

Peak calling parameters

Specify the command line program and parameters used for read mapping and peak calling, including the ChIP, control and index files used.

Data quality

Describe the methods used to ensure data quality in full detail, including how many peaks are at FDR 5% and above 5-fold enrichment.

Software

Describe the software used to collect and analyze the ChIP-seq data. For custom code that has been deposited into a community repository, provide accession details.

Flow Cytometry

Plots

Confirm that:

- [ ] The axis labels state the marker and fluorochrome used (e.g. CD4-FITC).
- [ ] The axis scales are clearly visible. Include numbers along axes only for bottom left plot of group (a 'group' is an analysis of identical markers).
- [ ] All plots are contour plots with outliers or pseudocolor plots.
- [ ] A numerical value for number of cells or percentage (with statistics) is provided.

Methodology

Sample preparation

Describe the sample preparation, detailing the biological source of the cells and any tissue processing steps used.

Instrument

Identify the instrument used for data collection, specifying make and model number.

Software

Describe the software used to collect and analyze the flow cytometry data. For custom code that has been deposited into a community repository, provide accession details.

Cell population abundance

Describe the abundance of the relevant cell populations within post-sort fractions, providing details on the purity of the samples and how it was determined.

Gating strategy

Describe the gating strategy used for all relevant experiments, specifying the preliminary FSC/SSC gates of the starting cell population, indicating where boundaries between "positive" and "negative" staining cell populations are defined.

- [ ] Tick this box to confirm that a figure exemplifying the gating strategy is provided in the Supplementary Information.

Magnetic resonance imaging

Experimental design

Design type

Indicate task or resting state; event-related or block design.

Design specifications

Specify the number of blocks, trials or experimental units per session and/or subject, and specify the length of each trial or block (if trials are blocked) and interval between trials.

Behavioral performance measures

State number and/or type of variables recorded (e.g. correct button press, response time) and what statistics were used to establish that the subjects were performing the task as expected (e.g. mean, range, and/or standard deviation across subjects).

Acquisition

Imaging type(s) | *Specify: functional, structural, diffusion, perfusion.*

Field strength | *Specify in Tesla*

Sequence & imaging parameters | *Specify the pulse sequence type (gradient echo, spin echo, etc.), imaging type (EPI, spiral, etc.), field of view, matrix size, slice thickness, orientation and TE/TR/flip angle.*

Area of acquisition | *State whether a whole brain scan was used OR define the area of acquisition, describing how the region was determined.*

Diffusion MRI ☐ Used ☐ Not used

Preprocessing

Preprocessing software | *Provide detail on software version and revision number and on specific parameters (model/functions, brain extraction, segmentation, smoothing kernel size, etc.).*

Normalization | *If data were normalized/standardized, describe the approach(es): specify linear or non-linear and define image types used for transformation OR indicate that data were not normalized and explain rationale for lack of normalization.*

Normalization template | *Describe the template used for normalization/transformation, specifying subject space or group standardized space (e.g. original Talairach, MNI305, ICBM152) OR indicate that the data were not normalized.*

Noise and artifact removal | *Describe your procedure(s) for artifact and structured noise removal, specifying motion parameters, tissue signals and physiological signals (heart rate, respiration).*

Volume censoring | *Define your software and/or method and criteria for volume censoring, and state the extent of such censoring.*

Statistical modeling & inference

Model type and settings | *Specify type (mass univariate, multivariate, RSA, predictive, etc.) and describe essential details of the model at the first and second levels (e.g. fixed, random or mixed effects; drift or auto-correlation).*

Effect(s) tested | *Define precise effect in terms of the task or stimulus conditions instead of psychological concepts and indicate whether ANOVA or factorial designs were used.*

Specify type of analysis: ☐ Whole brain ☐ ROI-based ☐ Both

Statistic type for inference | *Specify voxel-wise or cluster-wise and report all relevant parameters for cluster-wise methods.*

(See Eklund et al. 2016)

Correction | *Describe the type of correction and how it is obtained for multiple comparisons (e.g. FWE, FDR, permutation or Monte Carlo).*

Models & analysis

n/a	Involved in the study
☐	☐ Functional and/or effective connectivity
☐	☐ Graph analysis
☐	☐ Multivariate modeling or predictive analysis

Functional and/or effective connectivity | *Report the measures of dependence used and the model details (e.g. Pearson correlation, partial correlation, mutual information).*

Graph analysis | *Report the dependent variable and connectivity measure, specifying weighted graph or binarized graph, subject- or group-level, and the global and/or node summaries used (e.g. clustering coefficient, efficiency, etc.).*

Multivariate modeling and predictive analysis | *Specify independent variables, features extraction and dimension reduction, model, training and evaluation metrics.*
