# Supplementary figures and images for: Multi-omics profiling of cachexia-targeted tissues reveals a spatio-temporally coordinated response to cancer
Source: Nat Metab. 2026 Jan 15;8(1):237–59. doi: 10.1038/s42255-025-01434-3 (PMC12855018; doi:10.1038/s42255-025-01434-3)

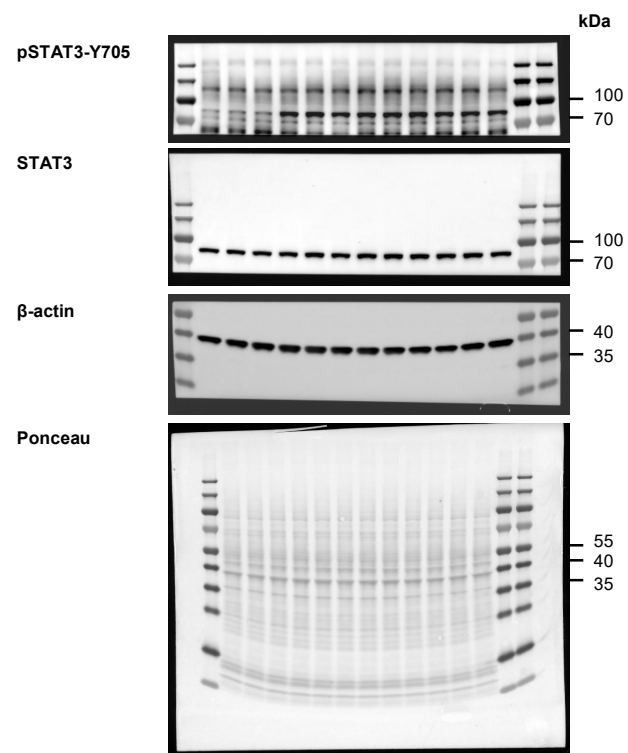

Source Data for Extended Data Fig. 6k

Supplement: Supplementary file 21 — Uncropped western blot. [file 42255_2025_1434_MOESM21_ESM.pdf]
